# High-Throughput Imaging of Blood Flow Reveals Developmental Changes in Distribution Patterns of Hemodynamic Quantities in Developing Zebrafish

**DOI:** 10.3389/fphys.2022.881929

**Published:** 2022-06-20

**Authors:** Swe Soe Maung Ye, Jung Kyung Kim, Nuria Taberner Carretero, Li-Kun Phng

**Affiliations:** ^1^ Laboratory for Vascular Morphogenesis, RIKEN Center for Biosystems Dynamics Research (BDR), Kobe, Japan; ^2^ School of Mechanical Engineering, Kookmin University, Seoul, South Korea

**Keywords:** zebrafish, development, live imaging, blood flow, blood viscosity, wall shear stress

## Abstract

Mechanical forces from blood flow and pressure (hemodynamic forces) contribute to the formation and shaping of the blood vascular network during embryonic development. Previous studies have demonstrated that hemodynamic forces regulate signaling and gene expression in endothelial cells that line the inner surface of vascular tubes, thereby modifying their cellular state and behavior. Given its important role in vascular development, we still know very little about the quantitative aspects of hemodynamics that endothelial cells experience due to the difficulty in measuring forces *in vivo*. In this study, we sought to determine the magnitude of wall shear stress (WSS) exerted on ECs by blood flow in different vessel types and how it evolves during development. Utilizing the zebrafish as a vertebrate model system, we have established a semi-automated high-throughput fluorescent imaging system to capture the flow of red blood cells in an entire zebrafish between 2- and 6-day post-fertilization (dpf). This system is capable of imaging up to 50 zebrafish at a time. A semi-automated analysis method was developed to calculate WSS in zebrafish trunk vessels. This was achieved by measuring red blood cell flow using particle tracking velocimetry analysis, generating a custom-made script to measure lumen diameter, and measuring local tube hematocrit levels to calculate the effective blood viscosity at each developmental stage. With this methodology, we were able to determine WSS magnitude in different vessels at different stages of embryonic and larvae growth and identified developmental changes in WSS, with absolute levels of peak WSS in all vessel types falling to levels below 0.3 Pa at 6 dpf. Additionally, we discovered that zebrafish display an anterior-to-posterior trend in WSS at each developmental stage.

## 1 Introduction

The establishment of a blood circulatory system is essential for the efficient transport of oxygen, metabolites and cells to all tissues of the body. Blood is pumped by the heart under high pressure and distributed through a network of tubular blood vessels comprised of arteries, capillaries and veins. The flow of viscous blood inflicts different mechanical stresses (hemodynamic forces) on endothelial cells (ECs) lining the inner surface of blood vessels. Blood flow imparts fluid shear stress, which is the tangential frictional force per unit area on ECs, while blood pressure exerts a normal force that compresses the EC apical surface (Dessalles et al., 2021). Besides driving the exchange of gases and solutes between the endothelium and surrounding tissues, the mechanical forces of blood have fundamental roles in the initial development of blood vessels and their subsequent remodeling by modulating EC behaviors through the mechanical forces imparted. During angiogenesis, pressurized blood flow is required for the formation of new vascular sprouts in certain vascular beds (Nicoli et al., 2010; Goetz et al., 2014; Ghaffari et al., 2015), the expansion and fusion of apical membranes in vessels undergoing transcellular lumen formation (Lenard et al., 2013; Gebala et al., 2016) and in maintaining vessel diameter (Bussmann et al., 2011; Baeyens et al., 2015; Nakajima et al., 2017). After the formation of a primitive vascular plexus, hemodynamics further regulate vessel pruning (Chen et al., 2012; Kochhan et al., 2013; Lenard et al., 2015) and vessel diameter (Udan et al., 2013; Sugden et al., 2017) to remodel the primitive vascular plexus into a hierarchical network of larger arteries and veins and smaller capillaries with optimal connections. Haemodynamic forces therefore have a major influence on the generation and shaping of the vascular tree (reviewed in Campinho et al., 2020; Phng and Belting, 2021). However, little is known about the magnitude of hemodynamic forces ECs are exposed to and how they vary spatiotemporally within a vascular network during different stages of development and growth.

In this study, we sought to determine how hemodynamics evolve during blood vessel development and remodeling using the zebrafish as a vertebrate model system. The zebrafish offers many advantages for several reasons: many eggs can be obtained from a single female zebrafish, eggs are rapidly and externally fertilized, zebrafish in early developmental stages are optically transparent and transgenic lines with specific fluorescently labelled cellular compartments exist (Streisinger et al., 1981; Weinstein, 2002). These features therefore permit live observation of many zebrafish throughout development. However, in practice, only a small fraction of zebrafish is analyzed due to the time-consuming and laborious nature of handling zebrafish specimens. As such, in previous studies that have investigated blood flow, the sample size of zebrafish analyzed has been small, ranging from 1 to 21 zebrafish and limited to a few vessel types and developmental stages (Malone et al., 2007; Chen et al., 2011; Watkins et al., 2012; Anton et al., 2013; Choi et al., 2017; Sugden et al., 2017; Follain et al., 2018; Santoso et al., 2019). Therefore, to reliably determine hemodynamic quantities over development, we aimed to increase the number of zebrafish that can be imaged and analyzed from 2 to 6 dpf. To achieve this, we established a semi-automated high-throughput imaging system to image red blood cell (RBC) flow in the entire zebrafish and an analysis pipeline to calculate RBC flow speed, lumen diameter, hematocrit, blood viscosity, pseudo shear rate, and wall shear stress in multiple vessel types of the zebrafish trunk.

## 2 Materials and Equipment

### 2.1 Zebrafish Handling

Zebrafish (*Danio rerio*) were raised and staged according to established protocols (Kimmel et al., 1995). Red blood cells were visualized using the transgenic line, *Tg(gata1:dsRed)* ^*sd2*^ (Traver et al., 2003). Endothelial cells were visualized using *Tg(kdrl:EGFP)* ^*s843*^ (Jin et al., 2005). 0–6 days post-fertilization (dpf) zebrafish were maintained at 28°C in E3 medium containing 0.003% phenylthiourea to inhibit melanogenesis. For imaging, 2–6 dpf zebrafish were anaesthetized in E3 medium containing 0.16 mg/ml Tricaine (Sigma-Aldrich). An agarose-based imaging chamber consisting of six grooved-lanes (similar to a microinjection plate) was used to mount dechorionated zebrafish. Zebrafish were placed in the lanes and fixed at their positions by pipetting 1% low-melting agarose (Bio-Rad) containing 0.16 mg/ml Tricaine until zebrafish and grooves were entirely covered and filled, respectively. The zebrafish were quickly aligned and positioned on their sides before the low-melting agarose has set. Once set, the mounting chamber was covered with E3 medium containing 0.16 mg/ml Tricaine.

### 2.2 Imaging System

We built a semi-automated, high-throughput imaging system consisting of an imaging chamber capable of mounting 50 zebrafish at a time, a robotic XY-stage scanning over 50 × 50 mm^2^ area, and a high-performance sCMOS camera (PCO, pco.edge 4.2 CL) mounted on a fluorescent stereomicroscope (Leica, M205FA). The sCMOS camera can capture images at 100 frames per second (fps) at 2048 x 2048 pixels full resolution. A stage-top incubator (Live Cell Instrument, Chamlide) was used to keep 2–6 dpf zebrafish at 27–28°C. We synchronized the robotic stages with the sCMOS camera to image many zebrafish for each stage movement. The positions of the zebrafish in the sample mounting chamber were pre-registered manually before imaging. Two actuators that were connected to each stage and controlled by a multi-dimensional acquisition option at the Micro-Manager were set up to move to the pre-registered positions throughout whole scanning area. The smallest distance that can be moved by the actuator was 0.0476 µm. A TTL (Transistor Transistor Logic) signal triggered the sCMOS camera with a time delay of half a second in response to a typical pulse waveform generated at the movement of the actuator. After the completion of each stage movement, a stack of 1,000 images per zebrafish was acquired at the frame rate of 100–180 fps and stored in an automatically generated folder. The size of one image stack was ∼1.3 GB.

## 3 Methods

### 3.1 Measuring Red Blood Cell Flow

RBC flow was imaged for 1,000 frames at 180 fps for zebrafish at 2 and 3 dpf while lower imaging rates of 100 and 120 fps were employed for zebrafish at 4, 5, and 6 dpf. Lower imaging rates were used for the later stages (4–6 dpf) to achieve longer observation time to capture sufficient network flow while maintaining manageable data volume as the RBCs flowed significantly slower than at 2 and 3 dpf stages.

We used the linear motion tracker in Image J software plug-in TrackMate (Tinevez et al., 2017) for particle tracking velocimetry (PTV) analysis. In order to obtain RBC velocities, the linear motion tracker algorithm performs pairing identification of the same RBC across two or more image frames. Newly appearing RBCs are paired with their previous position by searching for unpaired candidates from the previous image frame within an initial search radius (ISR). Previously tracked RBCs are paired with their new position using the expected position given by the previously predicted velocity, the highest correlated candidate for pairing is searched for within the maximum search radius (MSR) around that expected position. We optimized the tracker with an ISR of 25 µm and a MSR of 10 µm for extracting reliable trajectories from 1,000 images per zebrafish (Supplementary Videos S1–S5).

The TrackMate software for measuring RBC flow speeds was evaluated to be comparable with manual tracking of RBC trajectories. These validation results are shown in Supplementary Figure S1, where a single RBC was followed along its trajectory using both TrackMate and manual tracking (Supplementay Figure S1A). The graph of RBC speed against time obtained via both methods showed good correspondence between the two approaches (Supplementary Figure S1B), thus indicating the robustness of the TrackMate algorithm for RBC speed measurement even under high hematocrit conditions.

### 3.2 Automated Vessel Assignment, Rejection Assessment and Lumen Diameter Measurement

First, all zebrafish images were aligned such that the anterior to posterior (head to tail) axis was horizontally oriented from left to right while the ventral to dorsal axis was vertically oriented from bottom to top of the image. RBC trajectories from TrackMate were separated for vessel type according to the overall track direction (straight line path from the full trajectory of an RBC from start point to end point in the image time series). The dorsal longitudinal anastomotic vessel (DLAV) segments were identified by their dorsal location in the image and the rightward or leftward travelling track directions of RBCs. In the mid dorsal-ventral region of the image, rightwards travelling tracks corresponded to the dorsal aorta/caudal artery (DA/CA) segments while leftwards travelling tracks were assigned as posterior cardinal vein/caudal vein (PCV/CV) segments. Upwards travelling tracks were recognized as arterial intersegmental vessel (aISV) segments and downwards travelling tracks were assigned as venous intersegmental vessel (vISV) segments.

**FIGURE 1:**
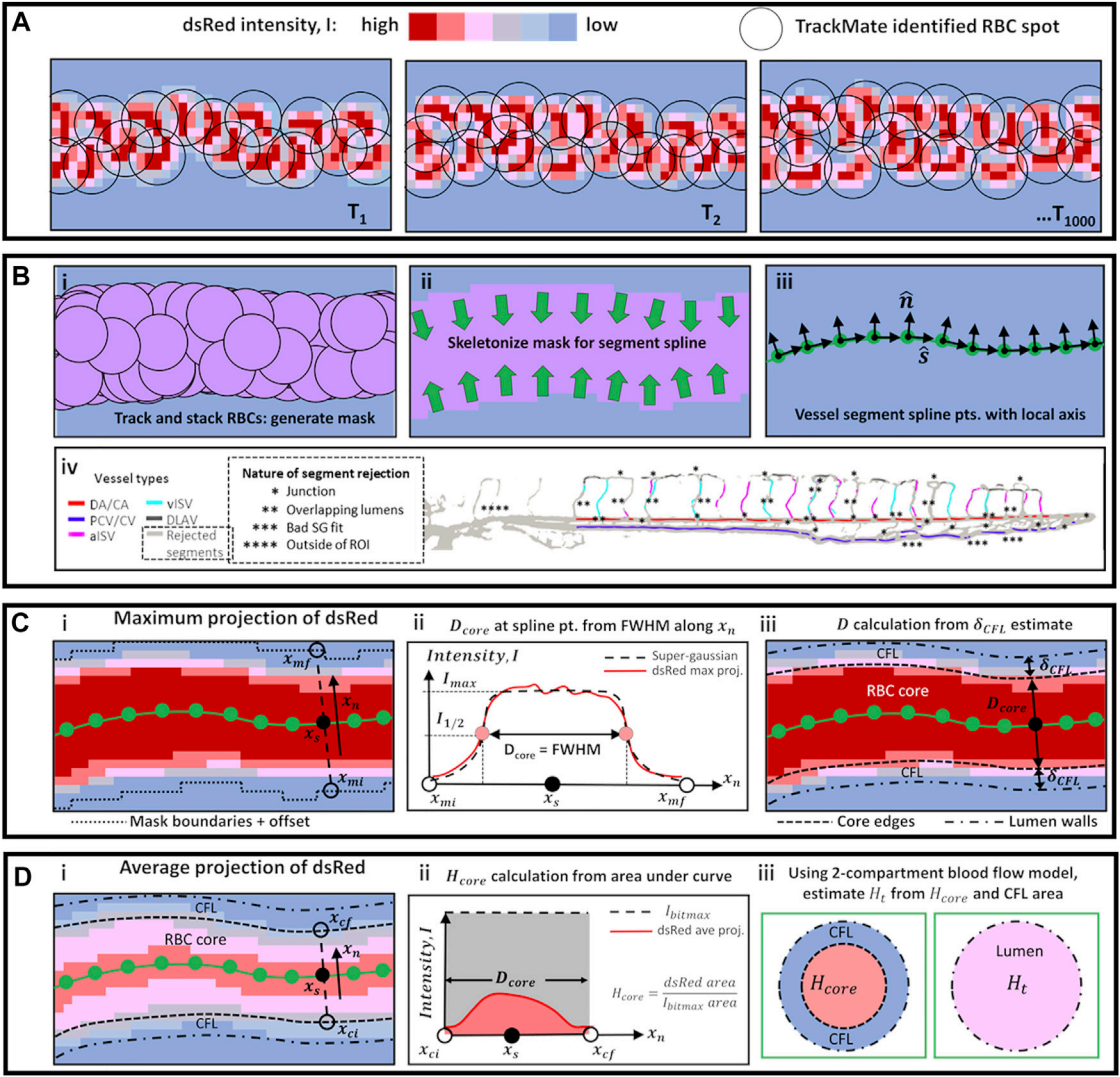
Schematic diagrams of methods used for automated vessel labeling, data filtering, and vessel diameter and hematocrit calculation. **(A)** High speed image sequence of dsRed intensity was the only collected data from experiments. **(B)** Series of steps using TrackMate data **(i–iii)** to obtain vessel spline segment points and directions $\hat{s}$ and $\hat{n}$ , and **(iv)** perform script-automated vessel-type labeling and data filtering from the spline segment skeletons in iii (see text for explanation). **(C)** Sequence of steps to calculate the width of the RBC core ( $D_{core}$ ) at a spline location **(i)** by evaluating the full-width-half-maximum (FWHM) range of the Super-gaussian fit to the maximum projection signal **(ii)**. The lumen diameter D is obtained by iterative evaluation (see text) of the cell-free plasma layer (CFL) thickness ( $\delta_{CFL}$ ). **(D)** Sequence of steps to calculate the core hematocrit ( $H_{core}$ ) at a spline location **(i)** by taking the area fraction between ceiling intensity ( $I_{bitmax}$ ) and the average projection signal within the RBC core width **(ii)**. The tube hematocrit in the lumen ( $H_{t}$ ) is evaluated by considering the effective contribution of RBC-rich core and plasma-rich CFL.

Using this assignment, we generated preliminary masks for each vessel type by stacking the corresponding TrackMate-identified RBC spots across the 1,000 time series images (Figures 1A,Bi). These masks were then skeletonized to provide skeletal connectivity maps of the vessel types (Figures 1Bii,iii). Next, we filtered the vessel-type-assigned data for quality and topological uniqueness. As our imaging technique superimposes data from multiple focal depth into a two-dimensional projection, vessel lumen diameter was impossible to measure when multiple regions merged in the projection to provide a combined luminal width. Hence, we rejected data from vessel locations where the skeletal maps coincided as this indicated topological ambiguity. This rejection criteria applied to regions where the ISVs joined with the DA/CA, PCV/CV or DLAV (* regions in Figure 1Biv) and most often in ISV locations where the imaging captured ISVs located on a higher focal plane running across or positioned very close to ISVs on a lower focal plane (** regions in Figure 1Biv). Finally, the topologically unique vessel segments were fitted to splines and smoothed with a Savitzky-Golay filter to determine spline coordinates ( $x_{s}$ ) with local vessel segment direction ( $\hat{s}$ ) and perpendicular direction ( $\hat{n}$ ) for the local diameter assessment (Figure 1Biii). After taking the maximum intensity projection image of dsRed-positive RBC pixels in the image time series, the fluorescence intensity profile along $\hat{n}$ at each spline point was used to estimate the width of RBC core ( $D_{core}$ ) in the vessel lumen segment (Figure 1Ci). Specifically, intensity profiles between mask edges $x_{mi}$ and $x_{mf}$ along the spline-perpendicular coordinate axis ( $x_{n}$ ) were fitted against a super-Gaussian (SG) function by non-linear least squares method:

$$I_{SG}=I_{max}e^{-{\left[\frac{{\left(x_{n}\;-\;x_{n,peak}\right)}^{2}}{2\sigma^{2}}\right]}^{k}}$$ (1)

where $I_{SG}$ and $I_{max}$ are the intensity and peak intensity of the SG respectively. $x_{n}$ is the position coordinate along the vessel perpendicular and $x_{n,peak}$ is the position coordinate of the SG peak along the $x_{n}$ coordinate axis. $\sigma^{2}$ is the variance of the fitted SG and k is the SG power coefficient, whereby a value of 1 reverts the SG back to a Gaussian. $D_{core}$ was estimated by the full width at half maximum (FWHM) value of the fitted SG profile (Figure 1Cii):

$$D_{core}=FWHM\;=\;2\sigma \sqrt[{2k}]{2ln2}$$ (2)

At vessel segments where the SG fit was poor ( $\chi^{2}$ > 100 from Eq. 3), the data associated with such segments were omitted from further analyses (*** regions in Figure 1).

$$\chi^{2}\;=\;\sum \frac{{\left(I_{mp}-I_{SG}\right)}^{2}}{N}$$ (3)

where $I_{mp}$ is the intensity of the dsRed maximum projection signal and N is the number of pixel points along the spline-perpendicular.

Next, the lumen diameter ( D ) was calculated by the sum of $D_{core}$ and the cell-free plasma layer (CFL) thickness ( $\delta_{CFL}$ ) which itself is a function dependent on D (Kim et al., 2007) (Figure 1Ciii). Since both $\delta_{CFL}$ and D are unknown and not directly measured in our imaging technique, we solved for these two inter-dependent parameters iteratively until the error ( ϵ ) was below 0.1% using the following sequence of calculations in Eqs 4–9 (note that all length variables must be in µm):

$$\delta_{CFL}^{*}=0;D^{*}=D_{core}+2\delta_{CFL}^{*}$$ (4)

where $\delta_{CFL}^{*}$ is given by Eq. 5 (Kim et al., 2007): $D^{*}$ and $\delta_{CFL}^{*}$ are the trial diameter and trial CFL to be optimized by iteration

$$\delta_{CFL}^{*}=\left(1.387ln\left(D^{*}/2\right)-1.463\right)$$ (5)

$$D_{old}^{*}=D^{*}$$ (6)

$$D^{*}=D_{core}+2\delta_{CFL}^{*}$$ (7)

$$\epsilon =\sqrt{{\left(D_{old}^{*}-D^{*}\right)}^{2}}/D_{old}^{*}$$ (8)

$$if\;\left\{\epsilon >0.001\;repeat\;equations\;5\;to\;8\right\}\;\;else\;\left\{D=\;D^{*}\;and\;\delta_{CFL}=\delta_{CFL}^{*}\right\}$$ (9)

### 3.3 Hematocrit, Apparent Blood Viscosity and Wall Shear Stress Calculation

A key consideration in our wall shear stress (WSS) calculation is the RBC phase contribution to apparent blood viscosity ( η ) in the zebrafish trunk vascular network. Namely, the lumen segment tube hematocrit ( $H_{t}$ ), which is the concentration ratio of RBC phase to lumen segment volume is required to represent the RBC influence on η . To do this, we first evaluated the RBC concentration ratio in the RBC core ( $H_{core}$ ) along the spline perpendicular ( $x_{n}$ ) at that spline segment location (Figures 1Di,ii):

$$H_{core}=\frac{1}{D_{core}}\int_{x_{n}=x_{ci}}^{x_{n}=x_{cf}}\left(I_{ap}/I_{bitmax}\right)dx_{n}$$ (10a)

where $I_{ap}$ is the signal intensity of the average projection of time-stacked images taken along $x_{n}$ . $I_{bitmax}$ is the ceiling intensity, for an 8-bit image this has a value of 255. $x_{ci}$ and $x_{cf}$ are the boundaries of the RBC core along $x_{n}$ . Eq. 10a is resolved in discrete form given by Eq. 10b:

$$H_{core}=\frac{1}{N}\overset{N}{\underset{1}{\sum }}I_{ap}/I_{bitmax}$$ (10b)

where N is the total number of discrete points taken along the $x_{n}$ analysis line within the RBC core width, $D_{core}$ at that particular spline segment location. Next, we consider a two-compartment representation of microhemodynamic flow where the cross-sectional perspective of the blood lumen is a dense RBC core that is enveloped by an annular cell-free plasma layer (CFL). Finally, the lumen segment tube hematocrit $H_{t}$ was given by the fraction of the RBC core cross sectional area against the lumen cross sectional area (Figure 1Diii):

$$H_{t}=H_{core}\times {\left(D_{core}/D\right)}^{2}$$ (11)

For calculating WSS we used the Hagen–Poiseuille formulation with the assumption of parabolic blood flow velocity profile in the lumen cross-section. Using the apparent blood viscosity ( η ), bulk flow velocity ( $\bar{U}$ ) and lumen diameter ( D ), WSS was given by Eq. 12a:

$$WSS=8\eta \bar{U}/D\;where\;we\;assume\;\bar{U}=0.5U_{RBC,centerline}$$ (12a)

**FIGURE 2:**
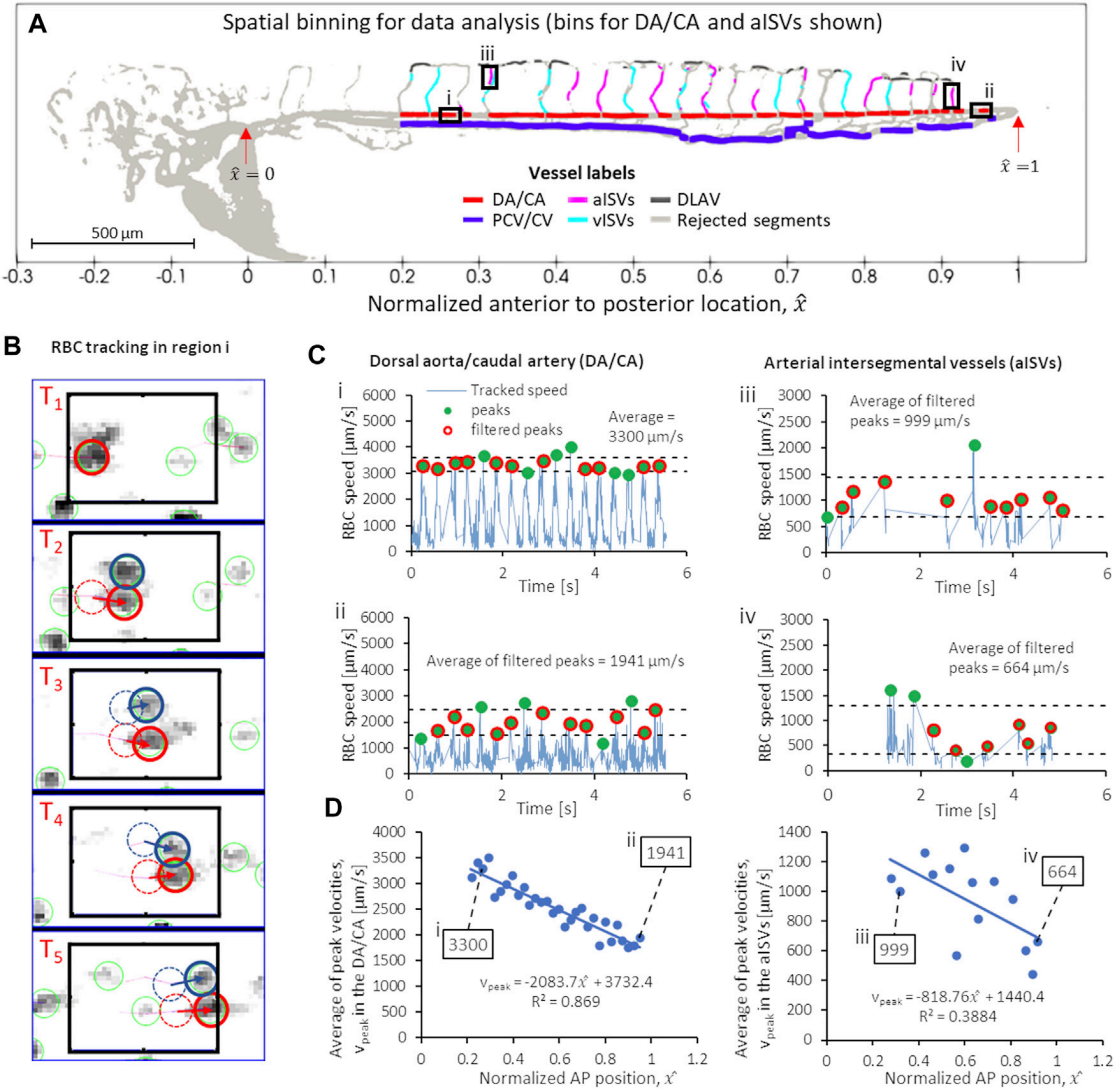
Series of script-automated steps to obtain peak (systolic) RBC velocity from TrackMate data, demonstrated using data from zebrafish 28 of the 2 dpf data set. **(A)** Velocity sampling within spatial bins of controlled intervals in different vessel types: shown in bold line black boxes are the 60-µm intervals for **(i)** a region in the anterior DA/CA, **(ii)** a region in the posterior DA/CA and ventral-to-dorsal intervals of filtered data (see text) for aISVs at the **(iii)** anterior and **(iv)** posterior regions of the trunk vasculature. **(B)** Example of TrackMate particle tracking algorithm performed in the anterior DA/CA region **(i)** shown in **(A)**. **(C)** Contiguous velocity against time curves and the application of peak scanning and outlier filtering to obtain the average peak (systolic) velocities in regions i, ii, iii and iv shown in **(A)**. **(D)** The anterior-to-posterior distribution of average peak velocities in the DA/CA (left) and aISVs (right) with the values for the four locations **(i–iv)** highlighted in **(A,C)** shown in boxes.

From the high-speed image acquisition of RBC flow, we obtained the RBC flow velocity ( $U_{RBC}$ ) at various locations in the network along the vessel axis (Figure 2A) using TrackMate plug-in in ImageJ (Figures 1A, 2B). We filtered for peak velocities ( $V_{peak}$ ) of RBC flow (Figure 2C) associated with the systolic peaks of lumen center-line RBC velocity ( $U_{RBC,centerline}$ ) (explained in greater detail in Section 3.4), thus the WSS calculated is given in the form of the systolic peak WSS ( $WSS_{peak}$ ):

$$WSS_{peak}=4\eta V_{peak}/D$$ (12b)

It is important to note that the apparent viscosity of blood ( η ) is not entirely an intrinsic property that arises from blood composition alone but also one that depends on extrinsic factors such as vessel diameter. Importantly, we implemented a model that describes the Fåhræus–Lindqvist (FL) effect observed in micro-vessels of 6–300 µm in diameter (Supplementary Figure S2), where flow resistance drops as vessel diameters reduce (Fåhræus–Lindqvist, 1931). For calculating η, the discharge hematocrit ( $H_{d}$ ) and curve-fitting coefficients ( γ ,  α , $\;\eta_{rel0.45}$ ) were calculated from empirical formulations reported by Fåhræus–Lindqvist, 1931 and Pries et al. (1992) in Eqs 13–17:

$$H_{d}=-\frac{\gamma }{2-2\gamma }+{\left[{\left(\frac{\gamma }{2-2\gamma }\right)}^{2}+\frac{H_{t}}{1-\gamma }\right]}^{0.5}$$ (13)

$$\;\;where\;\gamma =1+1.7e^{-0.35D}-0.6e^{-0.01D}$$ (14)

$$\eta =0.0012\left[1+\frac{\left(\eta_{rel0.45}-1\right)\left({\left(1-H_{d}\right)}^{\alpha }-1\right)}{{\left(1-0.45\right)}^{\alpha }-1}\right]\;$$ (15)

$$\;where\;\eta_{rel0.45}=220e^{-1.3D}+3.2-2.44e^{-0.06D^{0.645}}$$ (16)

$$\;\&\;\alpha =\left(0.8+e^{-0.075D}\right)\left(-1+\frac{1}{1+10^{-11}D^{12}}\right)+\frac{1}{1+10^{-11}D^{12}}$$ (17)

In addition to the WSS, we also calculated the peak pseudo shear rate PSR

$$PSR=V_{peak}/2D\;$$ (18)

Unlike the WSS, the PSR calculation is dependent only on the flow velocity and lumen diameter and is independent of the viscosity value which we could not directly measure. Thus, the PSR can be interpreted as a viscosity-independent indicator of the near wall shearing condition imparted by blood flow and can be useful in cases where viscosity is known to be relatively constant or uniform.

### 3.4 Spatial Binning of Tracked RBCs, Identification and Averaging of Temporal Peaks

In order to standardize the anterior to posterior (AP) trend comparison between zebrafish of varying sizes in our experiment, the AP axis was normalized for all our observations. The normalized AP coordinate, $\hat{x}$ is 0 at the junction where the common cardinal vein, anterior cardinal vein and posterior cardinal vein meet and $\hat{x}$ is 1 at the tail junction where the caudal artery joins with the caudal vein (Figure 2A). There was an overlapping of RBC flow in the 2D imaging between DA and PCV above the gut in all zebrafish across 2–6 dpf which corresponded to regions below $\hat{x}=0.2$ . These AP regions were deemed outside of the region of interest (ROI) and data there was discarded from subsequent analysis for all vessel types (Figures 1Biv, 2A).

To measure the temporal fluctuation of RBC flow velocity (and its WSS derivative) at various positions in the vascular network, we had to optimize a spatial sampling window that was large enough to contain a sufficient number of tracked RBCs in all image frame sequences. When the spatial sampling window was too narrow then a contiguous velocity fluctuation against time signal could not be constructed for that particular spatial position. We found that for DA/CA and PCV/CV, sampling windows of 60 µm intervals provided good velocity against time signals for analysis. Figures 2Ai,ii show two such intervals in the DA/CA taken at anterior and posterior locations and the resulting velocity against time signals are shown in Figure 2Ci,ii. In the DLAV, each interval was the available segment (after rejection discussed in Section 3.2) between two co-parallel ISVs. In aISVs and vISVs, we took available ISV segments (after rejection for poor imaging quality and topological non-uniqueness discussed in Section 3.2) separated by the DLAV segments as the sampling window. Figures 2Aiii,2Aiv show two such intervals for the aISVs and the resulting velocity against time signals are shown in Figures 2Ciii,iv. The aISVs show less regularity in the RBC velocity pulsation as compared to the DA/CA due to the sporadic nature of RBC flow into these vessels, unlike the DA/CA which is the major conduit for the RBC flow. Due to the regularity of the velocity pulsation in the anterior region of the DA/CA (Figure 2Ci), the velocity pulsation frequency was used to calculate the heartbeat in the zebrafish (heartbeats shown in Supplementary Figure S3). We validated this assumption in validation experiments detailed in methods Section 3.5.

From the temporal signals of velocity fluctuations at each spatial window, we identified the signal peaks in velocities (green circles in Figure 2C) and removed outlier peaks (peaks larger or smaller than the average peak by one standard deviation, shown by the dashed lines in Figure 2C) assuming that they were noise-contributed errors in the particle tracking algorithm. Finally, we applied ensemble averaging of the filtered peak velocities (red circles in Figure 2C) and took that value to be the representative peak velocity of RBC flow at that particular AP location. Figure 2D shows graphs of the average peak velocity against $\hat{x}$ for the DA/CA and aISVs, where the point annotated by box i corresponds to the averaging performed for filtered velocity peaks from Figure 2Ci, box ii corresponds to Figure 2Cii, box iii to Figure 2Ciii and box iv to Figure 2Civ.

### 3.5 Validation of Hematocrit, Diameter and Heartbeat Assessment

We performed an additional set of experiments using the double transgenic zebrafish line, *Tg(gata1:dsRed);Tg(kdrl:EGFP)*, where RBCs and ECs, respectively, can be simultaneously visualized. For this, we examined seven zebrafish at 2 dpf with varying degrees of hematocrit by injecting 1 nl of 0.1 mM Gata1 morpholino (Galloway et al., 2005). As the endothelial EGFP signal was not sharp under whole-zebrafish imaging conditions, we had to focus on the mid-trunk to tail region to increase the pixel resolution of the resulting image in the validation experiment (× 80 magnification) as compared to the main experiment (× 40). As presented in Supplementary Figure S4, two zebrafish injected with control morpholino (fish C1 and C2 in Supplementary Figures S4A,B and Supplementary Videos S6, S7) displayed typical levels of hematocrit. Among the Gata1 morphants, 3 zebrafish had moderate levels of hematocrit reduction (fish M1, M2, M3 in Supplementary Figures S4C–E and Supplementary Videos S8–S10) and 2 zebrafish had almost vanishing hematocrit levels (fish M4 and M5 in Supplementary Figures S4F,G and Supplementary Videos S11, S12). Compared against manual counting of RBCs, our method of using the average intensity correlation corroborated the trend of decreasing hematocrit in the DA/CA, PCV/CV and ISV (Supplementary Figures S4H–J) across the three hematocrit group ranges. Both approaches showed qualitative trend of decreasing hematocrit.

Using the same seven fish, we examined lumen diameters using the method in Section 3.2 and compared its results against a peak-to-peak distance approach using endothelial EGFP signal. Compared to diameters measured using the endothelial marker, diameters obtained using the method in 3.2 had average discrepancy levels of ± 17% in the CA, ± 11% in the CV and ± 22% in the smaller vessels like the ISVs (Supplementary Figure S5). We noted that the discrepancy tended to increase for smaller vessels and for vessels with very low local hematocrit (<0.01). Graphs of the dsRed maximum projection and super-gaussian fitting performed for this data set can be seen in Supplementary Figures S6–S8.

Validation of the heartbeat measurement was performed using zebrafish C2. We compared the velocity pulsation frequency at the anterior region of the DA (Supplementary Figures S9Ai,ii) against the heart wall displacement cycle (Supplementary Figures S9Bi,ii). Both methods indicated the same 180 bpm for zebrafish C2, thus indicating the direct correlation between velocity pulsation frequency in the DA and the zebrafish heartbeat.

## 4 Results

### 4.1 Microhemodynamics in the Zebrafish at Early Development

WSS is linearly proportional to blood velocity and viscosity but inversely proportional to the lumen diameter (Eq. 12). Using this relation, we sought to acquire coherent appreciation of the microhemodynamics at play and the developmental trends of these related quantities were analyzed in tandem for each vessel type.

**FIGURE 3:**
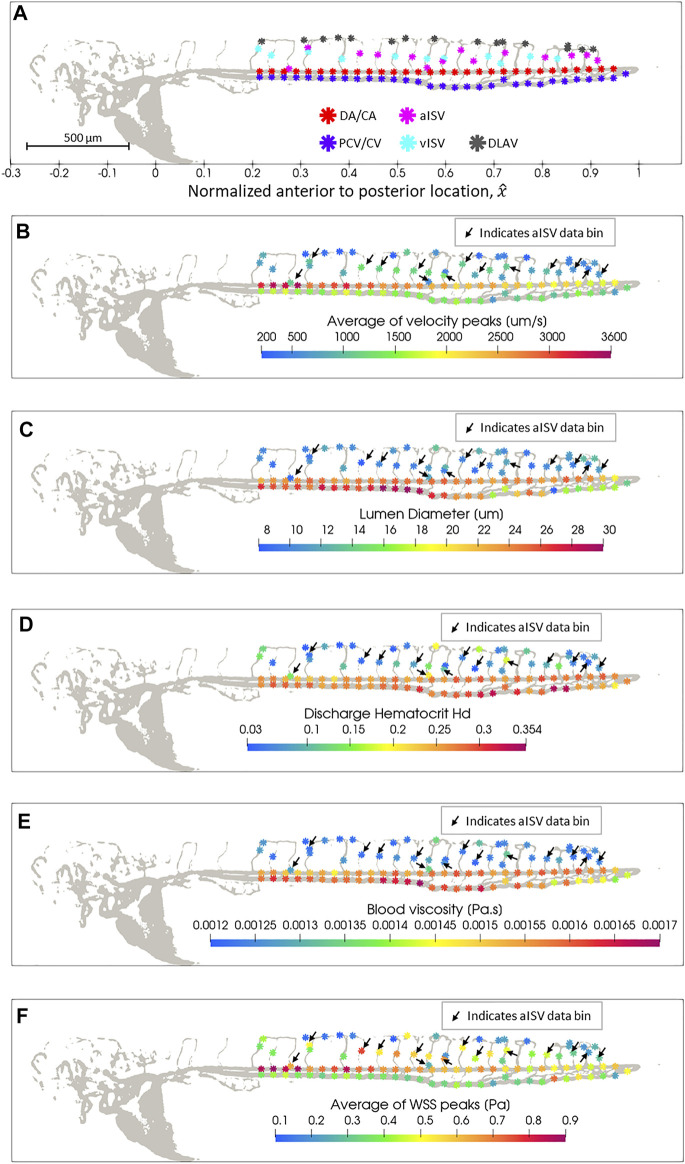
Spatial distribution map of data in zebrafish 28 of the 2 dpf data set after automated spatial bin averaging: **(A)** vessel type, **(B)** average velocity peak ( $V_{peak}$ ), **(C)** lumen diameter ( D ), **(D)** discharge hematocrit ( $H_{d}$ ), **(E)** apparent blood viscosity ( η ) and **(F)** average peak WSS ( $WSS_{peak}$ ). Arrows in Panels **(B–F)** indicate the aISV data. See Supplementary Video S13 for maps of hemodynamic quantities for all zebrafish across all the developmental stages analyzed.

We plotted the colorized magnitudes of hemodynamic quantities with respect to the spatial bins (defined in Section 3.4) for each vessel type in zebrafish 28 from the 2 dpf data set (Figure 3A) to provide a spatial distribution map of quantity levels in the zebrafish trunk network. There was a reduction in peak blood velocity towards the tail of the zebrafish in the main vessels (DA/CA and PCV/CV) and in the aISVs closer towards the tail (Figure 3B). PCV/CV showed reduction in diameters towards the tail while the DA/CA showed a similar but milder trend (Figure 3C). ISVs and DLAV segments were significantly smaller in diameter than the main vessels (Figure 3C) and a lower discharge hematocrit (RBC flow concentration) was observed in these smaller vessel types (Figure 3D). As a result of their lower discharge hematocrit, local viscosity of blood in the ISVs and DLAV was lower than the blood viscosity in main axial vessels (Figure 3E). The peak WSS trend in the DA/CA and aISVs showed a reduction in levels for vessel segments closer to the tail (Figure 3F), which closely correlated with the peak velocity trends for the two respective vessel types (Figure 3B). Interestingly, the peak WSS for PCV/CV showed a slight increase in levels accompanied by increasing spatial fluctuation in levels towards the tail (Figure 3F) despite falling peak velocities tailward in this vessel type (Figure 3B)—this was likely due to the significant diameter reduction tailward and the increasing spatial fluctuations of diameter in the CVP region (Figure 3C).

The trends in zebrafish 28 show the causal relationships between lumen diameter and discharge hematocrit; discharge hematocrit and blood viscosity; blood flow velocity, lumen diameter and WSS. However, since biological variations among zebrafish can be large (as shown in the individual zebrafish trends for hemodynamic quantities in Supplementary Figures S10–S34), the trends described for a single zebrafish may not be representative for an entire population. Hence, in order to study the developmental and spatial trends in the early development of zebrafish, we pooled the data from zebrafish at each stage of development. At 2, 3, 4, 5, and 6 dpf, we pooled data from 30, 32, 38, 35, and 29 zebrafish, respectively, for the spatial analysis of vessel morphology and hemodynamic trends (Supplementary Figure S35). Different zebrafish were used at each developmental stage.

**FIGURE 4:**
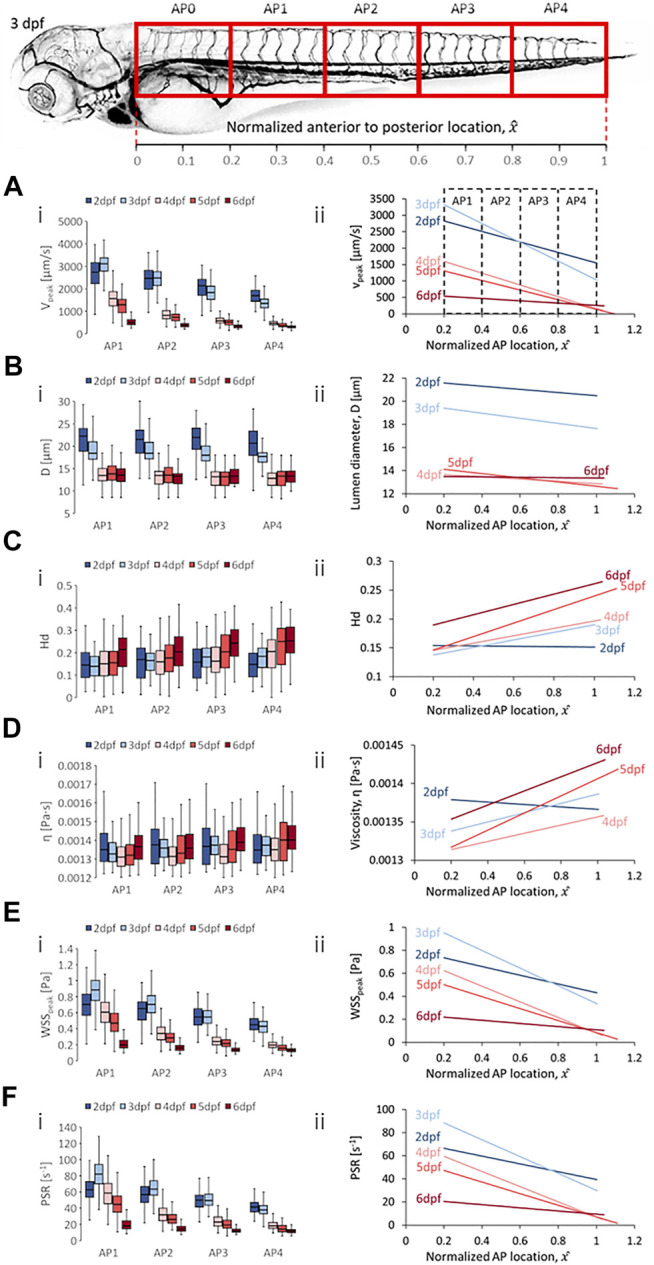
Developmental trends of morphological and hemodynamic quantities in the dorsal aorta/caudal artery (DA/CA). **(A)** The changes in $V_{peak}$ at four regions along the anterior-to-posterior (AP) axis **(i)** and the AP trend for $V_{peak}$ **(ii)**. **(B)** The changes in D at four AP regions **(i)** and the AP trend for D **(ii)**. **(C)** The changes in $H_{d}$ at four AP regions **(i)** and the AP trend for $H_{d}$ **(ii)**. **(D)** The changes in η at four AP regions **(i)** and the AP trend for η **(ii)**. **(E)** The changes in $WSS_{peak}$ at four AP regions **(i)** and the AP trend for $WSS_{peak}$ **(ii)**. **(F)** The changes in PSR at four AP regions **(i)** and the AP trend for PSR **(ii)**. Box plots in **(i)** of Panels **(A–D)** show the median and the first (Q1) and third (Q3) quartile levels of the hemodynamic quantity for the pooled zebrafish data. The whisker bars represent the maximum and minimum ranges of the data that lie within 1.5 times of the interquartile range (IQR = Q3 − Q1) beyond Q1 and Q3.

Two trend categories were summarized for all hemodynamic quantities analyzed. The first was the changes in magnitudes of quantities in each vessel type for zebrafish across developmental stages at four anterior-to-posterior (AP) group locations (Figures 4Ai–8Ai,Bi,Ci,Di,Ei,Fi) where the data was pooled along the AP axis: AP1 (pooling within 0.2 ≤ $\hat{x}$ < 0.4), AP2 (pooling within 0.4 ≤ $\hat{x}$ < 0.6), AP3 (pooling within 0.6 ≤ $\hat{x}$ < 0.8) and AP4 (pooling within 0.8 ≤ $\hat{x}$ < 1). The second trend was the spatial distribution of quantities in each vessel type along the AP axis of the fish—this was given by applying a linear regression model fit for the pooled data at each developmental stage (Figures 4Aii–8Aii,Bii,Cii,Dii,Eii,Fii) and discussing 1) the percentage change in quantity between $\hat{x}$ positions 0.2 and 1 given by the linear model and 2) the statistical significance of the regression slopes.

**FIGURE 5:**
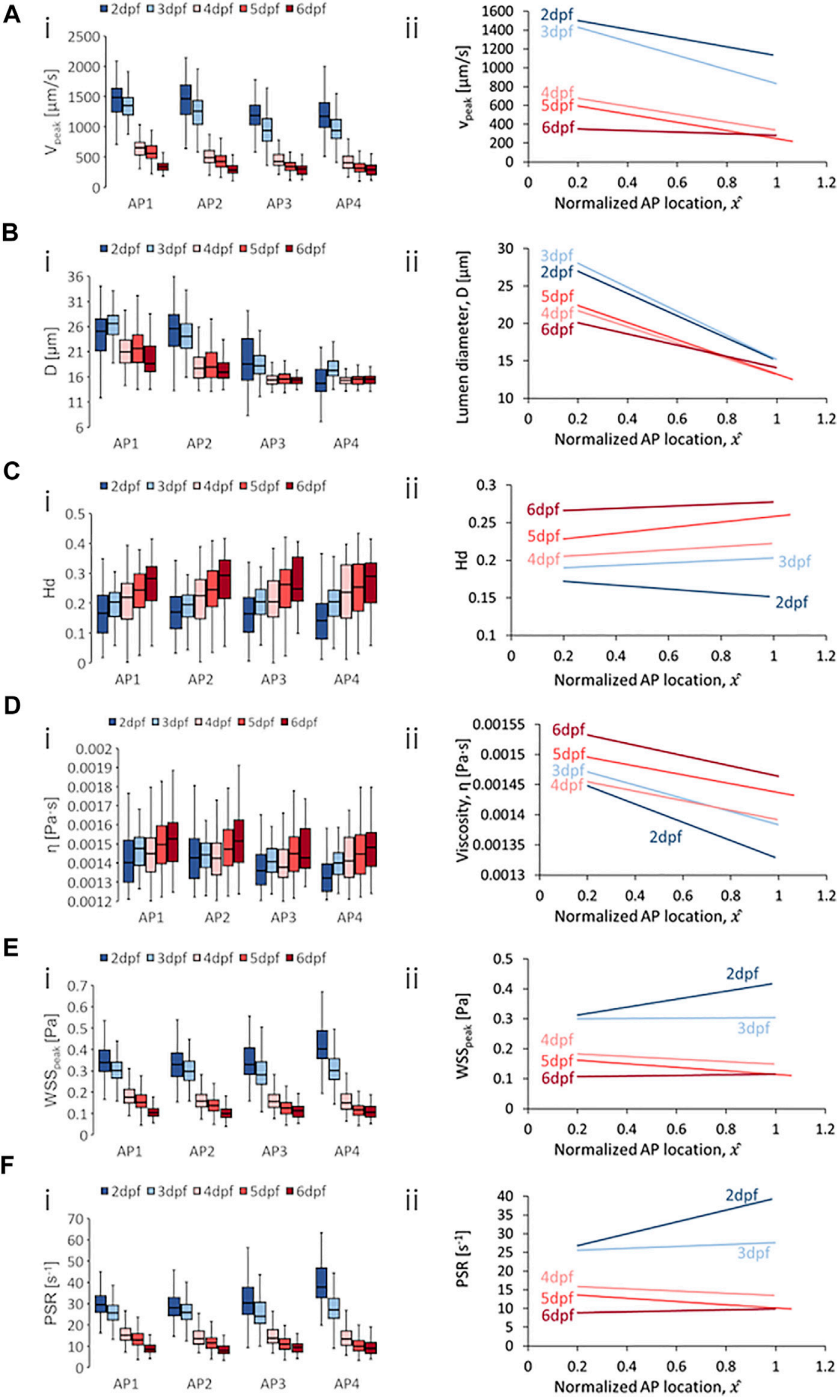
Developmental trends of morphological and hemodynamic quantities in the posterior cardinal vein/caudal vein plexus (PCV/CVP). **(A)** The changes in $V_{peak}$ at four regions along the anterior-to-posterior (AP) axis **(i)** and the AP trend for $V_{peak}$ **(ii)**. **(B)** The changes in D at four AP regions **(i)** and the AP trend for D (ii). **(C)** The changes in $H_{d}$ at four AP regions **(i)** and the AP trend for $H_{d}$ **(ii)**. **(D)** The changes in η at four AP regions **(i)** and the AP trend for η **(ii)**. **(E)** The changes in $WSS_{peak}$ at four AP regions **(i)** and the AP trend for $WSS_{peak}$ **(ii)**. **(F)** The changes in PSR at four AP regions **(i)** and the AP trend for PSR **(ii)**. Box plots in **(i)** of Panels **(A–D)** show the median and the first (Q1) and third (Q3) quartile levels of the hemodynamic quantity for the pooled zebrafish data. The whisker bars represent the maximum and minimum ranges of the data that lie within 1.5 times of the interquartile range (IQR = Q3 − Q1) beyond Q1 and Q3.

**FIGURE 6:**
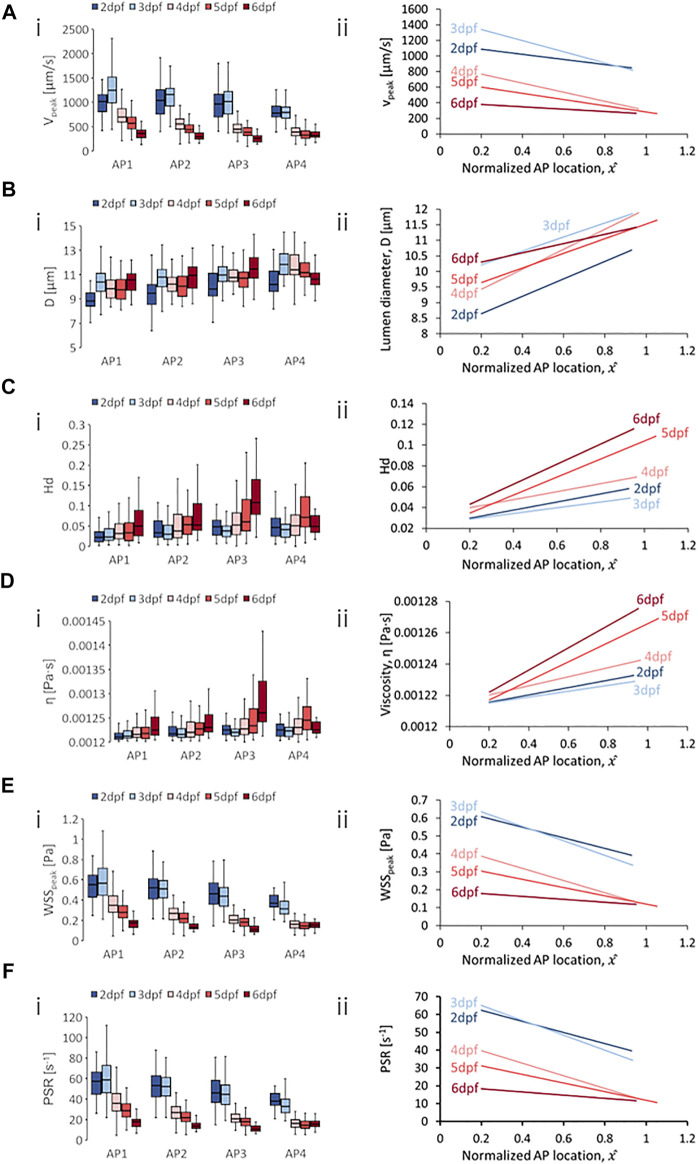
Developmental trends of morphological and hemodynamic quantities in the arterial intersegmental vessels (aISVs). **(A)** The changes in $V_{peak}$ at four regions along the anterior-to-posterior (AP) axis **(i)** and the AP trend for $V_{peak}$ **(ii)**. **(B)** The changes in D at four AP regions **(i)** and the AP trend for D **(ii)**. **(C)** The changes in $H_{d}$ at four AP regions **(i)** and the AP trend for $H_{d}$ **(ii)**. **(D)** The changes in η at four AP regions **(i)** and the AP trend for η **(ii)**. **(E)** The changes in $WSS_{peak}$ at four AP regions **(i)** and the AP trend for $WSS_{peak}$ **(ii)**. **(F)** The changes in PSR at four AP regions **(i)** and the AP trend for PSR **(ii)**. Box plots in **(i)** of Panels **(A–D)** show the median and the first (Q1) and third (Q3) quartile levels of the hemodynamic quantity for the pooled zebrafish data. The whisker bars represent the maximum and minimum ranges of the data that lie within 1.5 times of the interquartile range (IQR = Q3 − Q1) beyond Q1 and Q3.

**FIGURE 7:**
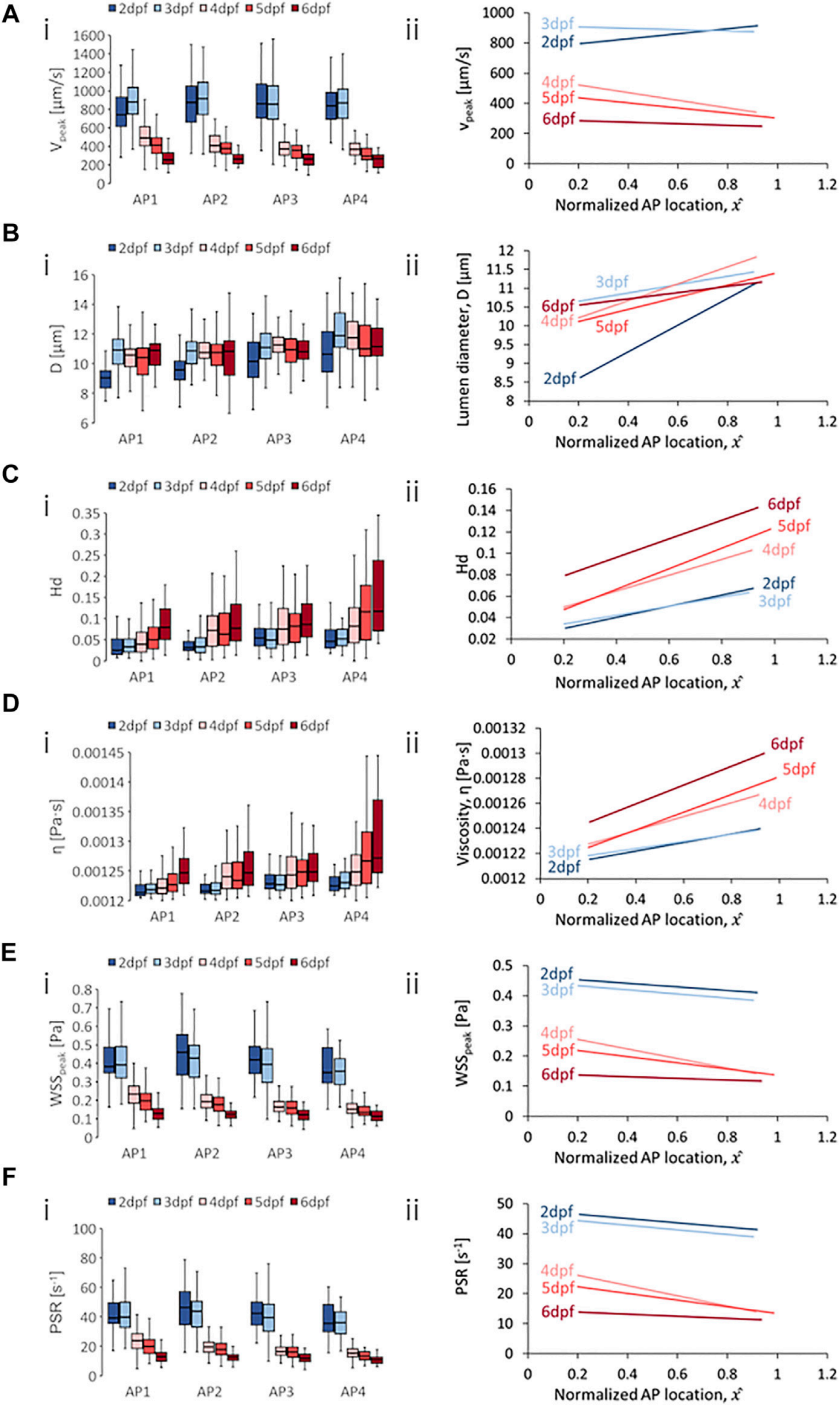
Developmental trends of morphological and hemodynamic quantities in the venous intersegmental vessels (vISVs). **(A)** The changes in $V_{peak}$ at four regions along the anterior-to-posterior (AP) axis **(i)** and the AP trend for $V_{peak}$ **(ii)**. **(B)** The changes in D at four AP regions **(i)** and the AP trend for D **(ii)**. **(C)** The changes in $H_{d}$ at four AP regions **(i)** and the AP trend for $H_{d}$ **(ii)**. **(D)** The changes in η at four AP regions **(i)** and the AP trend for η **(ii)**. **(E)** The changes in $WSS_{peak}$ at four AP regions **(i)** and the AP trend for $WSS_{peak}$ **(ii)**. **(F)** The changes in PSR at four AP regions **(i)** and the AP trend for PSR **(ii)**. Box plots in **(i)** of Panels **(A–D)** show the median and the first (Q1) and third (Q3) quartile levels of the hemodynamic quantity for the pooled zebrafish data. The whisker bars represent the maximum and minimum ranges of the data that lie within 1.5 times of the interquartile range (IQR = Q3 − Q1) beyond Q1 and Q3.

**FIGURE 8:**
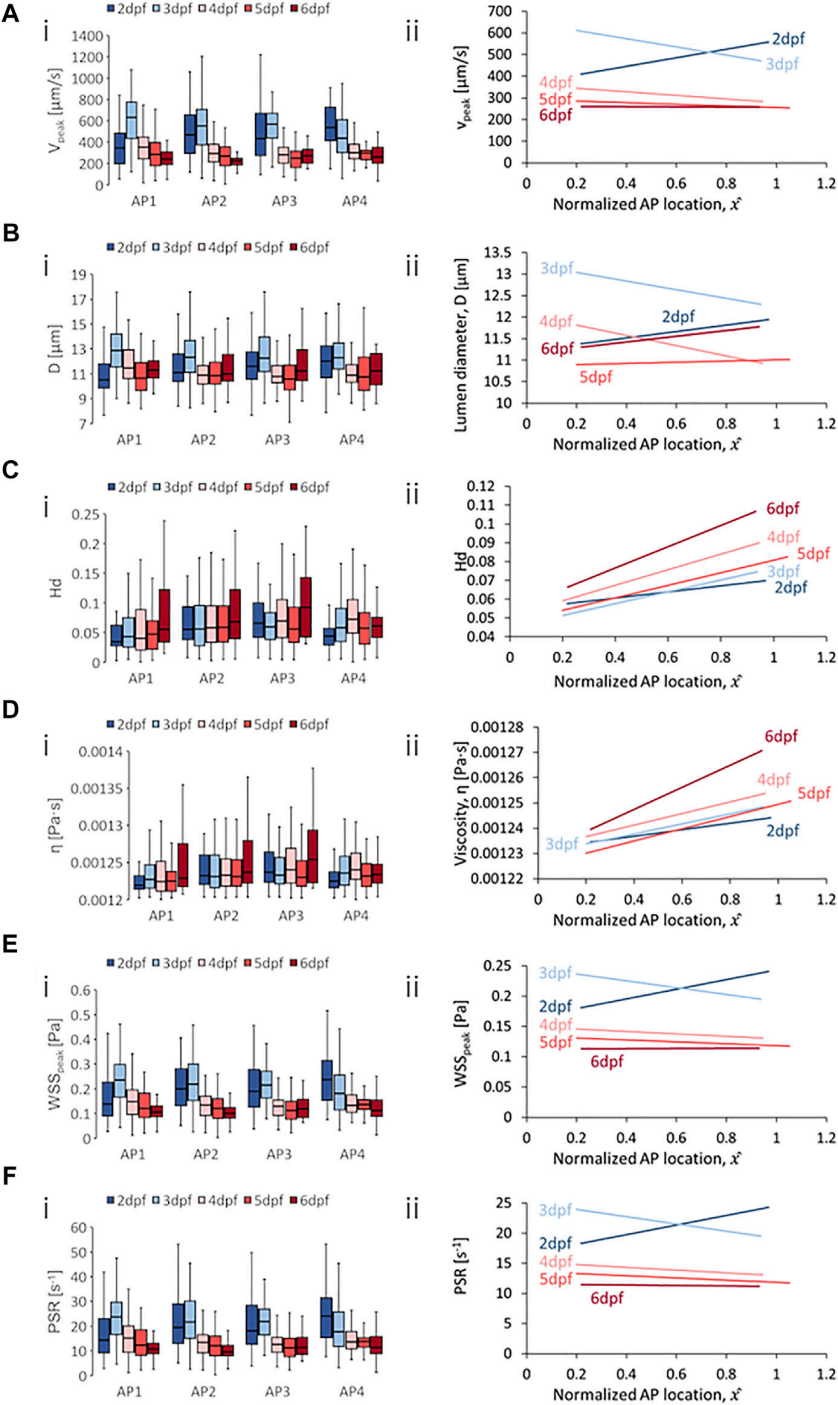
Developmental trends of morphological and hemodynamic quantities in the dorsal longitudinal anastomotic vessel (DLAV). **(A)** The changes in $V_{peak}$ at four regions along the anterior-to-posterior (AP) axis **(i)** and the AP trend for $V_{peak}$ **(ii)**. **(B)** The changes in D at four AP regions **(i)** and the AP trend for D **(ii)**. **(C)** The changes in $H_{d}$ at four AP regions **(i)** and the AP trend for $H_{d}$ **(ii)**. **(D)** The changes in η at four AP regions **(i)** and the AP trend for η **(ii)**. **(E)** The changes in $WSS_{peak}$ at four AP regions **(i)** and the AP trend for $WSS_{peak}$ **(ii)**. **(F)** The changes in PSR at four AP regions **(i)** and the AP trend for PSR **(ii)**. Box plots in **(i)** of Panels **(A–D)** show the median and the first (Q1) and third (Q3) quartile levels of the hemodynamic quantity for the pooled zebrafish data. The whisker bars represent the maximum and minimum ranges of the data that lie within 1.5 times of the interquartile range (IQR = Q3 − Q1) beyond Q1 and Q3.

### 4.2 Developmental and Anterior-to-Posterior Trend in DA/CA

Developmental trends for hemodynamic quantity levels in DA/CA showed a general reduction in median peak velocity ( $V_{peak})$ with a modest rise in median $V_{peak}$ from 2 to 3 dpf at AP1 and AP2 (Figure 4Ai; at 2, 3, 4, 5, 6 dpf, AP1: 2,745, 3,114, 1,568, 1,299, 499.2 µm/s; AP2: 2,462, 2,483, 826.0, 735.1, 376.8 µm/s; AP3: 2,143, 1834, 577.0, 518.6, 326.9 µm/s; AP4: 1,689, 1,344, 456.7, 361.5, 302.8 µm/s). The reduction in blood velocity during developmental progression suggested a higher fraction of blood from the heart was being directed to other essential organs in the zebrafish at the expense of trunk network flow. This was particularly so from 3 to 4 dpf where the precipitous fall in $V_{peak}$ contradicted the expected higher cardiac output attendant with the rise in average heart rate from 186 bpm at 3 dpf to 212 bpm at 4 dpf (Supplementary Figure S2). The closest quantities mirroring the $V_{peak}$ level developmental trend were the median $WSS_{peak}$ and median PSR level trends that saw general reduction in median levels across development with appreciable rise in median $WSS_{peak}$ and PSR from 2 to 3 dpf at AP1 and AP2 and no change in levels from 2 to 3 dpf at AP3 (Figure 4Ei for $WSS_{peak}$ ; at 2, 3, 4, 5, 6 dpf, AP1: 0.705, 0.885, 0.606, 0.468, 0.1973 Pa; AP2: 0.650, 0.705, 0.340, 0.282, 0.159 Pa; AP3: 0.548, 0.547, 0.241, 0.214, 0.135 Pa; AP4: 0.449, 0.429, 0.197, 0.157, 0.130 Pa, and Figure 4Fi for PSR ; at 2, 3, 4, 5, 6 dpf, AP1: 62.7, 82.0, 58.5, 44.7, 17.9 s^−1^; AP2: 56.7, 63.4, 31.8, 25.9, 14.4 s^−1^; AP3: 49.6, 49.4, 22.7, 19.1, 12.1 s^−1^; AP4: 41.5, 37.8, 17.9, 14.1, 11.4 s^−1^). The close correlation in the developmental trends between median $V_{peak}$ , $WSS_{peak}$ and PSR levels suggests that the dominant influencer of WSS and PSR in DA/CA for zebrafish in early development was the blood flow velocity.

Zebrafish also showed decreases in median diameter ( D ) levels from 2 to 4 dpf before exhibiting modest fluctuation in levels from 4 dpf onwards (Figure 4Bi; at 2, 3, 4, 5, 6 dpf, AP1: 22.3, 18.4, 13.5, 13.8, 13.51 µm; AP2: 21.5, 18.4, 13,5, 13.5, 13.3 µm; AP3: 22.0, 18.0, 13.2, 13.2, 13.3 µm; AP4: 20.7, 17.7, 12.8, 13.3, 13.3 µm). This developmental trend of decreasing lumen diameter corresponded with the reported trend of increasing vascular mural cell coverage in the DA beyond 2 dpf (Stratman et al., 2016). Furthermore, despite the progressive falling of median $V_{peak}$ levels beyond 4 dpf, the lack of further reductions in D thence suggests stabilization of the DA/CA lumen size by the increasing mural cell coverage. Median discharge hematocrit ( $H_{d})$ levels exhibited a gradual rise across development for AP4, a dip-and-rise trend that saddled to a minimum at 3 dpf for AP1 and at 4 dpf for AP2, and a general rising trend across development for AP3 with a drop from 3 to 4 dpf (Figure 4Ci; 2, 3, 4, 5, 6 dpf for each AP group, AP1: 0.145, 0.138, 0.151, 0.214; AP2: 0.169, 0.165, 0.160, 0.177, 0.204; AP3: 0.157, 0.181, 0.162, 0.203, 0.242; AP4: 0.148, 0.183, 0.205, 0.249, 0.252). Median blood viscosity ( η ) levels exhibited a dip-and-rise trend saddling to a minimum at 4 dpf for AP1 and AP2 while AP3 and AP4 saw an initial rise from 2 to 3 dpf followed by a similar dip-and-rise trend, saddling to a minimum at 4 dpf (Figure 4Di; 2, 3, 4, 5, 6 dpf, AP1: 0.001349, 0.001327, 0.00131, 0.001321, 0.001365 Pa s; AP2: 0.001376, 0.001359, 0.001314, 0.001331, 0.001358 Pa s; AP3: 0.001367, 0.001373, 0.001312, 0.001352, 0.001389 Pa s; AP4: 0.001348, 0.001374, 0.001349, 0.001401, 0.001402 Pa s). The saddling trend in median η levels can be explained by the sharp reduction in D between 3 and 4 dpf which by the Fåhræus–Lindqvist effect contributes towards the reduction of η . From 4 dpf onwards, without significant changes to D , $H_{d}$ was the dominant factor influencing η developmental trends.

In terms of spatial trends, we observed a consistent negative AP trend of decreasing quantity levels in the DA/CA for peak velocity ( $V_{peak}$ ) (Figure 4Aii), peak WSS ( $WSS_{peak}$ ) (Figure 4Eii) and peak PSR ( PSR ) (Figure 4Fii) at all developmental stages. A positive AP gradient trend of increasing quantity levels was observed for discharge hematocrit ( $H_{d}$ ) (Figure 4Cii) and blood viscosity ( η ) (Figure 4Dii) from 3 dpf onwards. Lumen diameter ( D) showed negative AP gradient levels (Figure 4Bii) from 2 to 5 dpf and a negligible gradient for D at 6 dpf (*p* = 0.607). The reduction in $V_{peak}$ towards the tail can be explained by the consecutive bifurcation of blood flow away from the DA/CA via the ISV network loops as blood travels along the AP axis in the DA. Plasma skimming into ISVs at these bifurcations contribute to the η rise in the DA/CA towards the tail for zebrafish from 3 dpf onwards as RBC concentration in the DA/CA blood flow increased, as highlighted by the $H_{d}$ trend (Eqs 13–15 dictate the positive effect of $H_{d}$ on η ). Interestingly, the increase in η towards the tail was modest (3.61%, 3.28%, 6.83%, and 5.41% increase along AP at 3, 4, 5 and 6 dpf) in comparison to the rise in $H_{d}$ (37.8%, 34.8%, 64.3%, and 37.8% increase along AP at 3, 4, 5, and 6 dpf). This was due to the mitigating effect of D reduction towards the tail (−5.03%, −9.13%, −5.77%, −10.3%, and −0.916% change along AP at 2, 3, 4, 5, and 6 dpf), which contributes to effective blood viscosity decrease in accordance with the Fåhræus–Lindqvist effect (Eqs 15–17 dictate the negative effect of D on η ). As put forward in Eq. 12b, $WSS_{peak}$ levels are directly proportional to η and $V_{peak}$ and inversely proportional to D . Despite the three-factor influence, the most apparent dominant contributor to the negative AP gradient for $WSS_{peak}$ (−41.4%, −64.4%, −87.4%, −83.3%, and −50.3% change along AP at 2, 3, 4, 5, and 6 dpf) was the negative AP gradient for $V_{peak}$ (−45.1%, −68.8%, −90.0%, −89.2%, and −52.8% change along AP at 2, 3, 4, 5 and 6 dpf). Similarly, the PSR was dominated by the influence of spatial distribution of $V_{peak}$ and the AP gradient for PSR was negative with very similar gradients levels (−40.6%, −66.0%, −88.1%, −84.9%, and −53.4% change along AP at 2, 3, 4, 5, and 6 dpf).

### 4.3 Developmental and Anterior-to-Posterior Trend in PCV/CV

Developmental trends for hemodynamic quantity levels in PCV/CV were generally similar to those observed in the DA/CA. Results showed a general reduction in median $V_{peak}$ with largest level changes between 3 and 4 dpf (Figure 5Ai; 2, 3, 4, 5, 6 dpf, AP1: 1,484, 1,351, 656.3, 341.7 µm/s; AP2: 1,468, 1,258, 491.4, 423.7, 279.3 µm/s; AP3: 1,185, 935.1, 427.8, 339.1, 301.5 µm/s; AP4: 1,176, 942.0, 401.3, 312.0, 288.8 µm/s). Closely mirroring the $V_{peak}$ level developmental trend were the median $WSS_{peak}$ and PSR level trends that saw general reduction in median levels across development (Figure 5Ei for $WSS_{peak}$ ; at 2, 3, 4, 5, 6 dpf, AP1: 0.339, 0.302, 0.177, 0.152, 0.104 Pa; AP2: 0.330, 0.298, 0.157, 0.138, 0.0989 Pa; AP3: 0.329, 0.281, 0.156, 0.126, 0.114 Pa; AP4: 0.403, 0.301, 0.150, 0.118, 0.105 Pa and Figure 5Fi for PSR ; at 2, 3, 4, 5, 6 dpf, AP1: 29.5, 25.6, 15.1, 13.1, 8.52 s^−1^; AP2: 28.1, 25.8, 13.6, 11.7, 7.94 s^−1^; AP3: 30.3, 24.1, 13.8, 10.9, 9.49 s^−1^; AP4: 37.8, 27.0, 13.3, 9.93, 8.95 s^−1^). The close correlation in the developmental trends between median $V_{peak}$ , median $WSS_{peak}$ and median PSR levels suggested that the dominant influencer of developmental changes in WSS and PSR in PCV/CV for zebrafish in early development was the blood flow velocity.

Zebrafish showed decreases in median D levels from 3 to 4 dpf followed by modest fluctuations in the quantity at all four AP regions. From 2 to 3 dpf, AP1 and AP4 showed increases in median D levels while AP2 and AP3 showed decrease (Figure 5Bi; 2, 3, 4, 5, 6 dpf, AP1: 25.1, 26.6, 20.9, 21.6, 18.7 µm; AP2: 25.6, 24.0, 17.7, 17.9, 16.9 µm; AP3: 18.6, 18.2, 15.4, 15.5, 15.4 µm; AP4: 14.7, 17.6, 15.3, 15.5, 15.5 µm). Median $H_{d}$ levels exhibited a gradual rise across development for all four AP regions with the exception of AP3 showing a slight drop in levels from 5 to 6 dpf (Figure 5Ci; 2, 3, 4, 5, 6 dpf, AP1: 0.166, 0.204, 0.220, 0.243, 0.283; AP2: 0.171, 0.195, 0.225, 0.245, 0.293; AP3: 0.165, 0.205, 0.262, 0.249; AP4: 0.142, 0.205, 0.237, 0.254, 0.290). Median η levels largely followed the median $H_{d}$ developmental trend which saw general increases in median η levels across development for all four AP regions (Figure 5Di; 2, 3, 4, 5, 6 dpf, AP1: 0.001403, 0.001477, 0.00145, 0.001496, 0.001527 Pa s; AP2: 0.001428, 0.001442, 0.001424, 0.001472, 0.001516 Pa s; AP3: 0.001359, 0.001406, 0.0013765, 0.001451, 0.001428 Pa s; AP4: 0.001321, 0.0014, 0.001411, 0.001448, 0.001481 Pa s).

With regards to spatial distribution trends in the PCV/CV, we observed consistent negative AP gradients for $V_{peak}$ , D , and η at all developmental stages (Figures 5Aii,Bii,Dii). $V_{peak}$ increased towards the head (reverse of the AP direction) for the PCV/CV as more blood entered the PCV/CV flow *via* consecutive ISV loops from the DA/CA towards the head (−24.7%, −42.0%, −50.0%, −58.1%, −19.5% change along AP at 2, 3, 4, 5, 6 dpf). D reduction towards the tail (−44.2%, −45.8%, −39.5%, −40.8%, −30.0% change along AP at 2, 3, 4, 5, 6 dpf) can be explained by the vascular anatomy as the PCV exists as a single tube at $\hat{x}$ < 0.5 while the CVP at $\hat{x}$ > 0.5 consists of a plexus network of multiple smaller vessels. $H_{d}$ showed no significant AP distribution trends at 2 dpf (−12.2% change along AP, *p* = 0.0597), 3 dpf (6.89%, *p* = 0.06), 4 dpf (8.37%, *p* = 0.147) and 6 dpf (4.30%, *p*=0.343) and a positive trend at 5 dpf where $H_{d}$ increased by 12.9% towards the tail (Figure 5Cii). Based on these gradient levels at different developmental stages, we concluded that there were no major spatial trends for $H_{d}$ along the AP axis between 2 and 6 dpf in our experiments. Given that there was no major trend in $H_{d}$ long the AP axis, η reduction towards the tail for all developmental stages in the PCV/CV was likely dictated by the Fåhræus–Lindqvist effect concomitant with D reduction towards the tail. The AP trend for $WSS_{peak}$ in the PCV/CV was positive for 2 dpf (33.9% increase along AP), negative for 4 dpf (−18.5%) and 5 dpf (−29.1%), and statistically insignificant for 3 dpf (1.49% increase, *p* = 0.624) and 6 dpf (8.24% increase, *p* = 0.115) (Figure 5Eii). As indicated in Figure 5Fii, the AP trend for PSR in the PCV/CV was positive for 2, 3 and 6 dpf (46.9, 8.10 and 12.1% increase along AP for the 2, 3, and 6 dpf stages) and negative for 4 dpf (−15.6%) and 5 dpf (−25.7%).

### 4.4 Developmental and Anterior-to-Posterior Trend in aISVs

Median levels of $V_{peak}$ in all four AP groups decreased after initial increases in levels from 2 to 3 dpf; the largest reductions in median $V_{peak}$ levels occurred between 3 and 4 dpf (Figure 6Ai; 2, 3, 4, 5, 6 dpf, AP1: 1,015, 1,248, 697.2, 565.2, 356.3 µm/s; AP2: 1,039, 1,157, 552.2, 441.3, 298.1 µm/s; AP3: 965.9, 1,015, 448.2, 259.0 µm/s; AP4: 774.4, 789.6, 388.4, 320.2, 317.8 µm/s). The falling $V_{peak}$ across development was a natural consequence of reduced blood flow in the feeding vessel DA/CA where aISVs draw their blood from. As with the DA/CA and PCV/CV vessel types, the $WSS_{peak}$ and PSR developmental trend in aISVs was closely related to the $V_{peak}$ trend. Median $WSS_{peak}$ and PSR saw general reductions in levels across development for all four AP groups with AP1 seeing an initial slight rise in levels from 2 to 3 dpf prior to reductions; AP4 seeing a slight rise in levels from 5 to 6 dpf after general reductions; and the largest reductions occurred from 3 to 4 dpf (Figure 6Ei for $WSS_{peak}$ ; at 2, 3, 4, 5, 6 dpf, AP1: 0.554, 0.568, 0.349, 0.282, 0.175 Pa; AP2: 0.523, 0.511, 0.269, 0.217, 0.136 Pa; AP3: 0.453, 0.437, 0.204, 0.183, 0.111 Pa; AP4: 0.369, 0.316, 0.162, 0.143, 0.155 Pa, and Figure 6Fi for PSR ; at 2, 3, 4, 5, 6 dpf, AP1: 57.3, 58.8, 35.8, 28.6, 17.8 s^−1^; AP2: 53.3, 52.2, 27.1, 21.9, 13.9 s^−1^; AP3: 45.9, 44.7, 20.6, 18.1, 10.7 s^−1^; AP4: 38.0, 32.7, 16.2, 14.5, 15.5 s^−1^). The close correlation in the developmental trends between median $V_{peak}$ , median $WSS_{peak}$ and median PSR levels suggested that the dominant influencer of developmental changes in WSS and PSR in aISVs for zebrafish in early development was the blood flow velocity.

One developmental trend distinctly different than those from DA/CA and PCV/CV was the diameter evolution. Zebrafish showed rising D levels from 2 to 3 dpf at all four AP regions that corresponded well with the velocity increase across these stages. This correlation supported the previously reported outward remodeling of vessels when flow increases (Langille and O’Donnell, 1986; Sugden et al., 2017). Following this rise, changes in D levels beyond 3 dpf were oscillatory at AP1 to AP3 and reducing at AP4, suggesting complex remodeling mechanisms beyond the correlation between flow velocity and vessel diameter (Figure 6Bi; 2, 3, 4, 5, 6 dpf, AP1: 8.83, 10.4, 9.84, 9.74, 10.6 µm; AP2: 9.47, 10.8, 10.2, 10.0, 10.9 µm; AP3: 9.83, 11.0, 10.8, 10.7, 11.5 µm; AP4: 10.2, 11.8, 11.4, 11.2, 10.6 µm). Median $H_{d}$ levels and median η levels exhibited similar rising level trends across development at each of the AP regions: AP1 showed a consistent rise in median levels for both quantities across 2–6 dpf; AP2 and AP3 saw a fall and rise trend saddling to a minimum for both quantities at 3 dpf before consistent increase across 3–6 dpf; AP4 saw an initial fall from 2–3 dpf followed by a peaking at 5 dpf before falling again at 6 dpf to a level similar to that at 2 dpf (Figure 6Ci for $H_{d}$ , 2, 3, 4, 5, 6 dpf, AP1: 0.0222, 0.0223, 0.0316, 0.0338, 0.0503; AP2: 0.0337, 0.0302, 0.0382, 0.0533, 0.0527; AP3: 0.0487, 0.0380, 0.0526, 0.0602, 0.108; AP4: 0.0467, 0.0414, 0.0512, 0.0713, 0.0495; and Figure 6Di for η , AP1: 0.001211, 0.001212, 0.001216, 0.001218, 0.001225 Pa s; AP2: 0.001217, 0.001216, 0.00122,0, 0.001227, 0.001230 Pa s; AP3: 0.001225, 0.001220, 0.001227, 0.001234, 0.001261 Pa s; AP4: 0.001225, 0.001223, 0.00123, 0.001245, 0.001225 Pa s). The dominant effector of developmental changes in median η levels was the median $H_{d}$ levels. Interpreting the rise in median $H_{d}$ levels across development for AP1 to AP3, it is possible that the maintenance of median D with oscillatory levels in these same regions after 3 dpf is a morphological optimization in the vascular remodeling that attempted to mitigate against the lower oxygen perfusion concomitant with falling median $V_{peak}$ levels by making aISVs more perfusable to RBCs.

For spatial trends in quantity distribution, we observed consistent negative AP gradients in the aISVs for $V_{peak}$ , $WSS_{peak}$ , and PSR at all developmental stages ( $V_{peak}$ changes along AP for 2, 3, 4, 5, 6 dpf in Figure 6Aii: −24.0%, −42.8%, −60.6%, −52.9%, −31.8%; $WSS_{peak}$ changes along AP for 2, 3, 4, 5, 6 dpf in Fig. 6Eii: 38.9%, −51.1%, −70.3%, −60.3%, −36.2%; PSR changes along AP for 2, 3, 4, 5, 6 dpf in Figure 6Fii: −39.9%, −51.4%, −71.2%, −58.5%, −39.0%). High pressure on the anterior end of the DA/CA drives DA/CA blood flow towards its posterior end where the pressure is moderate. Likewise, a moderate pressure on the posterior end of the PCV drives blood flow back towards the heart at the anterior end where pressure is low. aISVs and vISVs form arcades between DA/CA and PCV/CV where blood flow is driven by the pressure difference between the two main vessels of the trunk network. As a result of this anatomical design, pressure differences across aISVs and vISVs generally reduce in the AP direction. Consequently, the lowering of pressure differences lower median $V_{peak}$ levels in the aISVs located further down the AP axis. A mitigation against reduced pressure differences along AP is the shortening of ISV lengths to increase the flow-driving pressure gradient. Another mitigating feature by morphological design is increasing lumen diameters which we observed along the AP axis for D (25.9%, 17.6%, 27.2%, 19.5%, 11.6% increase along AP for 2, 3, 4, 5, 6 dpf). Increasing D permits higher RBC perfusion in the aISVs further down the AP axis to compensate for reduced oxygen perfusion concomitant with lower blood flow in these vessels. This possible compensatory mechanism was evinced by the rise in $H_{d}$ levels in aISVs further down the AP axis (107%, 75.9%, 77.9%, 200%, 179% increase along AP for 2, 3, 4, 5, 6 dpf).

η also exhibited a positive AP gradient that although statistically significant, was a mild level of change along the AP axis that did not augment $WSS_{peak}$ . From the comparison of AP trends among quantities, the strongest influencer of the negative $WSS_{peak}$ AP gradient was the negative $V_{peak}$ AP gradient.

### 4.5 Anterior-to-Posterior Trend in vISVs Across Development

Results for the vISVs showed a general reduction in median levels of $V_{peak}$ in all four AP regions after initial increases in levels from 2 to 3 dpf, except for AP3 that saw a small drop in levels from 2 to 3 dpf. The largest reductions for all regions occurred between 3 and 4 dpf for all regions (Figure 7Ai; 2, 3, 4, 5, 6 dpf, AP1: 742.4, 879.9, 493.2, 411.5, 254.0 µm/s; AP2: 874.9, 917.9, 409.6, 375.8, 261.9 µm/s; AP3: 863.0, 854.2, 373.6, 359.3, 260.7 µm/s; AP4: 836.3, 872.1, 367.2, 294.8, 269.5 µm/s). As vISVs serve the second half of the ISV loops between DA/CA and PCV/CV, falling $V_{peak}$ levels in DA/CA and aISVs also similarly affected the $V_{peak}$ levels in vISVs. Median $WSS_{peak}$ and PSR saw general reduction in levels across development for all four AP groups with AP1 and AP4 seeing initial slight rise in levels from 2 to 3 dpf prior to reductions; the largest reductions occurred from 3 to 4 dpf for all AP groups (Figure 7Ei for $WSS_{peak}$ ; 2, 3, 4, 5, 6 dpf, AP1:0.383, 0.391, 0.234, 0.197, 0.128 Pa; AP2: 0.459, 0.429, 0.193, 0.178, 0.125 Pa; AP3: 0.419, 0.394, 0.162, 0.159, 0.121 Pa; AP4: 0.351, 0.357, 0.152, 0.134, 0.113 Pa and Figure 7Fi for PSR ; at 2, 3, 4, 5, 6 dpf, AP1: 39.2, 39.9, 23.8, 19.9, 12.9 s^−1^; AP2: 45.5, 43.8, 19.6, 17.8, 12.7 s^−1^; AP3: 42.4, 39.4, 16.4, 16.2, 11.7 s^−1^; AP4: 35.5, 36.2, 15.3, 13.2, 10.9 s^−1^). The close correlation in the developmental trends between median $V_{peak}$ , median $WSS_{peak}$ and PSR levels suggested that the dominant influencer of developmental changes in WSS and PSR in vISVs for zebrafish in early development was the blood flow velocity.

Zebrafish showed a sharply rising median levels of D from 2 to 3 dpf for all AP regions which correlated well with increases in $V_{peak}$ in AP1, AP2, and AP4 but not AP3. Beyond 3 dpf, the median levels of D fluctuated around the 3 dpf level at all four AP regions (Figure 7Bi; 2, 3, 4, 5, 6 dpf, AP1: 9.05, 10.9, 10.6, 10.4, 10.9 µm; AP2: 9.59, 10.9, 10.75, 10.76, 10.85 µm; AP3: 10.2, 11.1, 11.3, 11.0, 10.8 µm; AP4: 10.6, 11.9, 11.7, 11.0, 11.2 µm). Median $H_{d}$ levels and median η levels exhibited similar developmental trends that saw levels consistently rise in all regions across 2–6 dpf, with AP3 showing an initial fall in both quantity medians from 2 dpf to 3 dpf and AP2 showing a brief fall in median levels for both quantities from 4 dpf to 5 dpf (in Figure 7Ci for $H_{d}$ at 2, 3, 4, 5, 6 dpf, AP1: 0.0254, 0.0336, 0.0399, 0.0504, 0.0790; AP2: 0.0317, 0.0331, 0.0727, 0.0631, 0.0775; AP3: 0.0548, 0.0500, 0.0752, 0.0829, 0.0865; AP4: 0.0461, 0.0526, 0.0819, 0.116, 0.117, and Figure 7Di for η , AP1: 0.001214, 0.001218, 0.001221, 0.001227, 0.001247 Pa s; AP2: 0.001216, 0.001217, 0.001240, 0.001234, 0.001247 Pa s; AP3: 0.001228, 0.001227, 0.001243, 0.001248, 0.001249 Pa s; AP4: 0.001225, 0.001230, 0.001249, 0.001267, 0.001272 Pa s). The dominant effector of developmental changes in median η levels for the vISVs was the developmental trend for median $H_{d}$ levels. As with the aISVs, we think the rise in median $H_{d}$ levels across development for all four AP regions in the vISVs indicates a morphological optimization that seeks to maximize oxygen perfusion and RBC flow by maintaining the median D after 3 dpf despite the falling median $V_{peak}$ .

For spatial trends in quantity distribution, we observed negative AP gradients in the vISVs for $V_{peak}$ and $WSS_{peak}$ at 4–5 dpf while gradients at 2, 3, and 6 dpf were statistically insignificant for both quantities ( $V_{peak}$ changes along AP for 2, 3, 4, 5, 6 dpf in Figure 7Aii: 16.9 (*p* = 0.107), −3.83 (*p* = 0.526), −38.9, -31.2, −14.3% (*p* = 0.0872) and $WSS_{peak}$ changes along AP for 2, 3, 4, 5, 6 dpf in Figure 7Eii: −10.4 (*p* = 0.274), −12.5 (*p* = 0.108), −50.0%, −37.5%, −16.0% (*p* = 0.103). Like aISVs, we observed that D in vISVs further down the AP axis were dilated, only at 6 dpf was the dilation found to be statistically insignificant (33.1, 8.42, 17.6, 12.9, 6.43 (*p*=0.158) % increase along AP for 2, 3, 4, 5, 6 dpf in Figure 7Bii). We believe the AP trend of vISV dilation towards the tail worked in tandem with the similar aISV trend to ensure higher RBC perfusion in the ISV loops further down the AP axis to compensate for reduced blood flow in these vessels. Proof of this is the rise in $H_{d}$ levels in vISVs further down the AP axis (138%, 96.3%, 119%, 160%, 87.6% increase along AP for 2, 3, 4, 5, 6 dpf in Figure 7Cii).

η exhibited a mild positive AP gradient that was statistically significant across all developmental stages (2.26%, 1.88%, 3.58%, 4.63%, 4.81% increase along AP for 2, 3, 4, 5, 6 dpf in Figure 7Dii). Despite the developmental increase in η AP gradients, the mild level of change did not augment $WSS_{peak}$ AP trend and the strongest influencer of the negative $WSS_{peak}$ AP gradient was the negative $V_{peak}$ AP gradient. Likewise, the strongest influencer of the negative PSR AP gradient ( PSR changes along AP for 2, 3, 4, 5, 6 dpf in Figure 7Fii: −12.3 [*p* = 0.194), −13.9 (*p* = 0.0746), −51.8%, −40.0%, −19.9%] was the negative $V_{peak}$ AP gradient.

### 4.6 Anterior-to-Posterior Trend in DLAV Across Development

DLAV segments exhibited a general reduction in median $V_{peak}$ levels across development for all AP regions after the initial sharp rise in median levels from 2 to 3 dpf (Figure 8Ai; 2, 3, 4, 5, 6 dpf, AP1: 346.1, 633.3, 354.9, 284.2, 243.0 µm/s; AP2: 469.9, 551.7, 293.6, 269.2, 225.2 µm/s; AP3: 433.6, 567.5, 282.8, 250.0, 275.4 µm/s; AP4: 536.6, 437.6, 303.5, 296.0, 263.7 µm/s). Forming similar developmental trends were the median levels of $WSS_{peak}$ and PSR which generally fell across development after initial rise in levels during 2–3 dpf for regions AP1, AP2, and AP3. AP4 saw gradual reductions in median levels of $WSS_{peak}$ and PSR across development (Figure 8Ei for $WSS_{peak}$ ; 2, 3, 4, 5, 6 dpf, AP1: 0.137, 0.236, 0.148, 0.121, 0.106 Pa; AP2: 0.200, 0.218, 0.134, 0.122, 0.101 PA; AP3: 0.189, 0.215, 0.129, 0.111, 0.118 Pa; AP4: 0.237, 0.181, 0.133, 0.136, 0.113 Pa and Figure 8Fi for PSR ; at 2, 3, 4, 5, 6 dpf, AP1: 14.3, 23.8, 15.1, 12.2, 10.7 s^−1^; AP2: 19.45, 21.7, 13.4, 12.1, 9.54 s^−1^; AP3: 13.0, 21.9, 12.6, 11.2, 11.4 s^−1^; AP4: 24.1, 17.8, 13.6, 13.8, 11.4 s^−1^).

Diameters exhibited a dynamic trend that was oscillatory in all four AP regions in the DLAV segments and there was no clear directionality in the developmental trend for median levels of D (Figure 8Bi; 2, 3, 4, 5, 6 dpf, AP1: 10.5, 12.9, 11.5, 10.6, 11.3 µm; AP2: 11.1, 12.3, 10.9, 10.9, 11.0 µm; AP3: 11.6, 12.2, 10.8, 10.6, 11.2 µm; AP4: 12.0, 12.3, 10.9, 10.7, 11.2 µm). Median levels of $H_{d}$ and η in DLAV segments both generally rose across development with undulating patterns in development along developmental time (Figure 8Ci for $H_{d}$ at 2, 3, 4, 5, 6 dpf, AP1: 0.0345, 0.0433, 0.0401, 0.0473, 0.0555; AP2: 0.0551, 0.0558, 0.0582, 0.0592, 0.0683; AP3: 0.0662, 0.0593, 0.0694, 0.0561, 0.0928; AP4: 0.0438, 0.0584, 0.0725, 0.0577, 0.0609 and in Fig. 8Di for η at 2, 3, 4, 5, 6 dpf, AP1: 0.001219, 0.001227, 0.001224, 0.001225, 0.001229 Pa s; AP2: 0.001232, 0.001231, 0.001233, 0.001231, 0.001237 Pa s; AP3: 0.001237, 0.001233, 0.001240, 0.001230, 0.001254 Pa s; AP4: 0.001225, 0.001236, 0.001240, 0.001232, 0.001234 Pa s). The $H_{d}$ and η rises in DLAV segments with development were a consequence of their feeding aISVs receiving higher RBC perfusion as described in Section 4.4.

Spatial trends for DLAV segments saw negative AP gradients for $V_{peak}$ at 3 and 4 dpf with statistically insignificant gradient levels at 2, 5, and 6 dpf [ $V_{peak}$ changes along AP for 2, 3, 4, 5, 6 dpf in Figure 8Aii: 38.9 (*p* = 0.0542), −24.8, −19.2, −10.4 (*p* = 0.264), −0.849% (*p* = 0.959)]. The AP gradient for $WSS_{peak}$ was negative at 3 dpf but statistically insignificant at other developmental stages [ $WSS_{peak}$ changes along AP for 2, 3, 4, 5, 6 dpf in Figure 8Eii: 35.8 (*p* = 0.0947), −19.1, −10.8 (*p* = 0.181), −9.84 (*p* = 0.309), −0.849% (*p* = 0.983)]. Likewise, AP gradient for PSR was negative at 3 dpf but statistically insignificant at other developmental stages [ PSR changes along AP for 2, 3, 4, 5, 6 dpf in Figure 8Fii: 35.4 (*p* = 0.0984), −20.0, −12.6 (*p* = 0.122), −11.0 (*p* = 0.261), −2.66% (*p* = 0.869)]. The AP gradients for D were largely statistically insignificant across development except for a mild negative AP gradient at 4 dpf [ D changes along AP for 2, 3, 4, 5, 6 dpf in Figure 8Bii: 5.25 (*p* = 0.375), −6.18 (*p* = 0.0648), −8.03, 1.01 (*p* = 0.75), 4.76% (*p* = 0.413)]. DLAV segments form the connecting passages between ipsilateral and contralateral ISVs and receive blood flow primarily from the DA/CA *via* aISVs. Although the anterior DLAV connects to the primordial hindbrain channel at 2.5 dpf and the basilar artery by 5 dpf, these events did not appear to affect AP gradient for $V_{peak}$ during the development between 2 and 6 dpf in our zebrafish. As $V_{peak}$ , D and $WSS_{peak}$ generally do not exhibit strong AP gradient trends across most developmental stages, we believe DLAVs do not have a clear trunk-wise directionality in the hemodynamic nor morphological tuning with regards to the AP axis. The evolution of this vessel type probably acquiesces to the local connectivity needs and flow requirements between neighboring ISVs.

The $H_{d}$ and η quantities both show positive AP gradients that saw levels rise towards the tail across most developmental stages except for statistically insignificant gradient levels at 2 and 6 dpf [ $H_{d}$ changes along AP for 2, 3, 4, 5, 6 dpf in Figure 8Cii: 22.9 (*p* = 0.355), 48.8%, 55.8%, 49.4%, 69.3% (*p* = 0.0790) and η changes along AP for 2, 3, 4, 5, 6 dpf in Figure 8Dii: 0.837 (*p* = 0.327), 1.30%, 1.48%, 1.58%, 2.82% (*p* = 0.0733)]. The positive AP trends for $H_{d}$ and η quantities in the DLAV segments resulted from the progressive increase in RBC flow in aISVs located further down the AP axis.

### 4.7 Summary of Anterior-to-Posterior Trends

**FIGURE 9:**
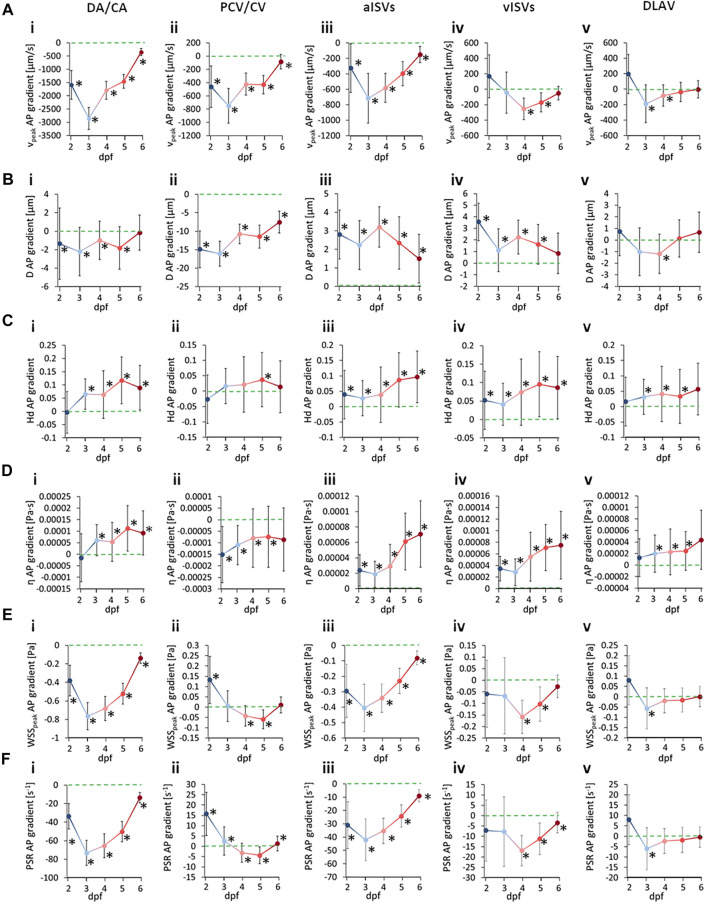
Developmental patterns of the anterior-to-posterior (AP) gradients in morphological and hemodynamic quantities in the zebrafish trunk vasculature. Circles indicate the hemodynamic AP gradient obtained from linear regression fitting of the pooled AP data, whisker bars indicate the standard error of the regression and * symbols denote statistically significant gradients from the slope T-test (*p* < 0.05). **(A)** AP gradients for $V_{peak}$ in the DA/CA **(i)**, PCV/CV **(ii)**, aISVs **(iii)**, vISVs **(iv)** and DLAV **(v)**. **(B)** AP gradients in D in the DA/CA **(i)**, PCV/CV **(ii)**, aISVs **(iii)**, vISVs **(iv)** and DLAV **(v)**. **(C)** AP gradients for $H_{d}$ in the DA/CA **(i)**, PCV/CV **(ii)**, aISVs **(iii)**, vISVs **(iv)** and DLAV **(v)**. **(D)** AP gradients for η in the DA/CA **(i)**, PCV/CV **(ii)**, aISVs **(iii)**, vISVs **(iv)** and DLAV **(v)**. **(E)** AP gradients for $WSS_{peak}$ in the DA/CA **(i)**, PCV/CV **(ii)**, aISVs **(iii)**, vISVs **(iv)** and DLAV **(v)**. **(F)** AP gradients in PSR in the DA/CA **(i)**, PCV/CV **(ii)**, aISVs **(iii)**, vISVs **(iv)** and DLAV **(v)**.

We summarized the changes in AP gradients across development time in Figure 9 for quantities (A) $V_{peak}$ (B) D (C) $H_{d}$ (D) η (E) $WSS_{peak}$ and (F) PSR in the vessel types (i) DA/CA, (ii) PCV/CV, (iii) aISVs, (iv) vISVs and (v) DLAV. The * symbols in Figure 9 denote the gradient levels deemed to be statistically significant (*p* < 0.05) from zero. For reference to the quantitated gradient magnitudes and slope difference statistical comparison between developmental stages, the reader may refer to Supplementary Tables S1–S5. From the summary trends in Figure 9, with the exception of η and $H_{d}$ , there appears to be an asymptotic developmental trend in the distribution of all quantities towards a zero AP gradient level from 2 to 6 dpf. This suggested a developmental progression towards distribution homogeneity in systolic blood flow, vessel diameters and systolic WSS distribution.

**FIGURE 10:**
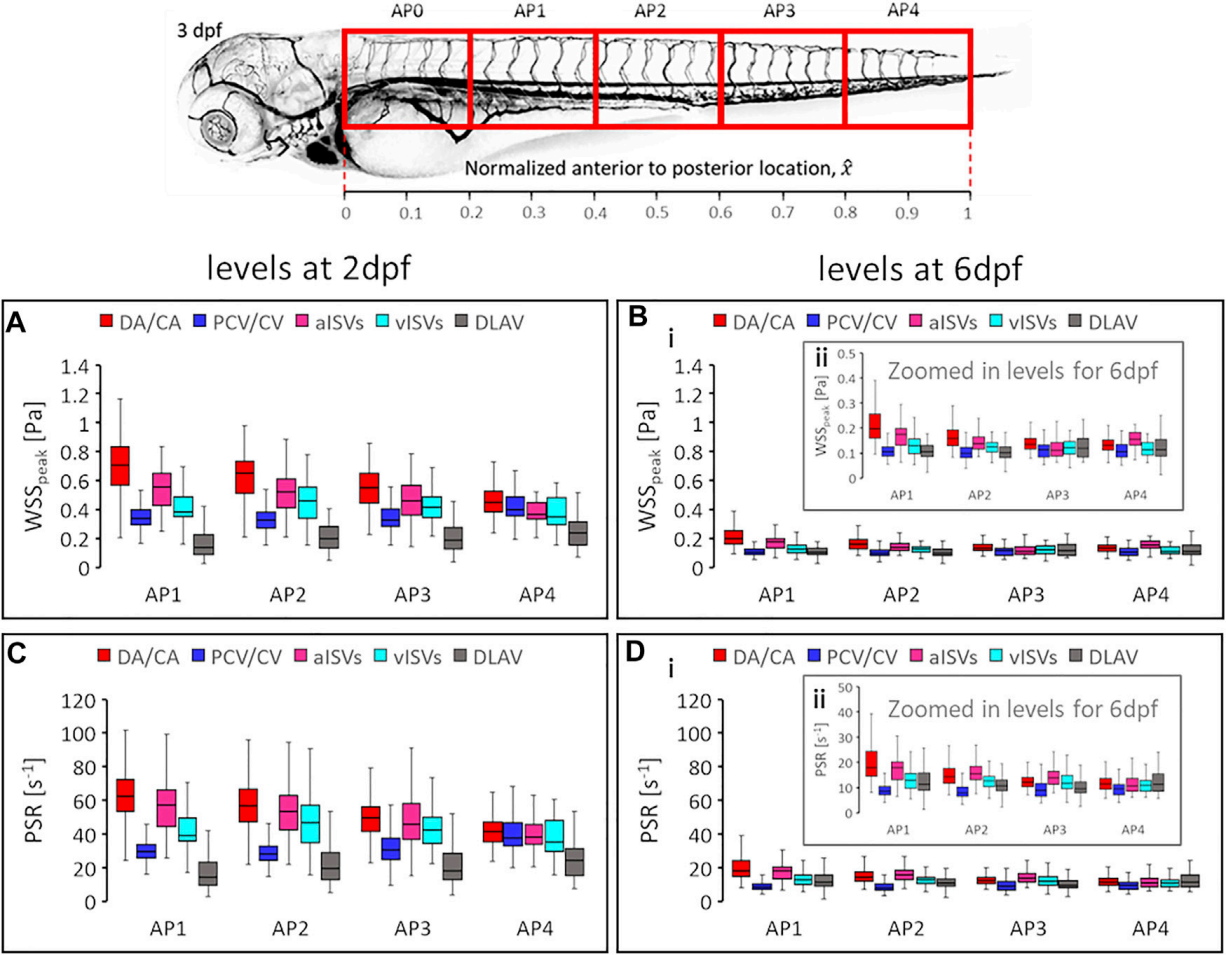
Developmental changes in magnitude levels of shear rate related quantities. The $WSS_{peak}$ and its hierarchy order among vessel types are shown for 2 dpf **(A)** and 6 dpf **(Bi**,**ii)**. The PSR and its hierarchy order among vessel types are shown for 2 dpf **(C)** and 6 dpf **(Di,ii)**. Box plots in graphs show the median and the first (Q1) and third (Q3) quartile levels of the hemodynamic quantity for the pooled zebrafish data. The whisker bars represent the maximum and minimum ranges of the data that lie within 1.5 times of the interquartile range (IQR = Q3 − Q1) beyond Q1 and Q3.

For the case of peak WSS comparison between vessel types, we observed that the median levels of $WSS_{peak}$ at 2 dpf demonstrated a clear hierarchy in levels between vessel types (Figure 10A). The hierarchy of $WSS_{peak}$ levels from highest to lowest was DA/CA, aISVs, vISVs, PCV/CVP, and DLAVs in all AP regions except for AP4 where PCV/CVP recorded the second-highest $WSS_{peak}$ . The median $WSS_{peak}$ dropped significantly from levels at 2 dpf to below 0.3 Pa for all vessel types across all AP regions by 6 dpf (Figure 10Bi). Although stratification order of median levels of $WSS_{peak}$ between vessel types was maintained in the anterior half of the zebrafish, the posterior half had a different $WSS_{peak}$ hierarchy establishment between 6dpf (Figure 10Bii) and 2 dpf stages. At 6 dpf, the order of $WSS_{peak}$ in regions AP1 and AP2 from highest to lowest was DA/CA, aISVs, vISVs, and similar levels between PCV/CV and DLAV; in region AP3, DA/CA had the highest levels while other vessel types had similar levels; in region AP4, aISVs showed higher levels than DA/CA while other vessel types showed similar levels. Like the AP gradient developmental trends, the reduction in vessel type differences in $WSS_{peak}$ levels at 6 dpf in the posterior half of zebrafish in most vessel types suggested a developmental progression towards distribution homogeneity of WSS in the zebrafish. Developmental evolution of peak PSR across the various spatial regions saw very similar trends to the peak WSS. Like the $WSS_{peak}$ , the PSR fell from high levels at 2 dpf (Figure 10C) to below 30 s^−1^ at 6 dpf (Figure 10Di) for all vessel types across all AP regions. The hierarchy of PSR levels between the vessel types for all AP regions at 2 dpf (Figure 10C) was similar to the $WSS_{peak}$ hierarchy. By 6 dpf the same hierarchy seen at 2 dpf was maintained in AP1, AP2, and AP3 for all the vessel types except for the PCV/CV which now became the vessel type with the lowest PSR (Figure 10Dii). In AP4, the PSR hierarchy could not be seen for all the vessel types except the PCV/CV which was lower than its peers. The subtle differences for changes in vessel hierarchy from 2 to 6 dpf for PSR levels versus the WSS levels in the posterior half and for PCV/CV in all regions can be understood to be the contribution of viscosity and hematocrit enrichment of blood towards the posterior end of the fish which was reflected in the calculation of WSS but not for PSR.

## 5 Discussion

In this study, we have developed a high-throughput protocol to image and analyze blood flow in order to estimate systolic wall shear stress (WSS) in the zebrafish trunk network. The aim of our study was to investigate the spatial and developmental trends in WSS distribution and vascular diameters in a developing microvascular network using the zebrafish trunk network as our animal model. To achieve the high-throughput imaging, we implemented a semi-automated zebrafish mounting and imaging protocol that can image RBC flow up to 50 zebrafish in one imaging sequence. We imaged zebrafish at 2, 3, 4, 5, and 6 days post-fertilization (dpf) for spatial and developmental trends in hemodynamics. To achieve high-throughput data analysis, lumen diameter, hemodynamic quantity calculation and data filtering were automated using our custom-written Python and C scripts. A key point to note is that our approach required only fluorescent labelling of RBCs and evaluation of their trajectories from the imaging to perform all the analyses in our study.

One limitation with our current protocol was the low imaging resolution, which was 1.625 µm for 2 dpf images and 1.85 µm for 3 dpf onwards. This setting was a consequence of prioritizing whole zebrafish imaging over high-resolution imaging in order to facilitate high-throughput imaging protocol since our robotic stage for ROI scanning required manual pre-registration of the imaging positions (which we aimed to minimize) on the mounting chamber. Further improvement to the protocol can be achieved with higher automation in the ROI registration process and image post-processing in order to obtain higher resolution images of the zebrafish trunk vasculature. We found that the levels of changes in median D across developmental time and changes in D along AP axis in the ISVs and DLAV segments were on the same order as the imaging resolution. This limited the precision of our technique and prevents our method from being used in a quantitative manner. However, the high throughput afforded by our automated diameter and data assessment approach in addition to the ∼50 zebrafish per imaging sequence allows our analysis to confidently discuss qualitative trends and relationships between hemodynamic quantities and vessel morphologies.

With regards to our WSS calculation, a key component is the evaluation of the apparent blood viscosity in micron-sized vessels (Eqs 13–17). In this respect, it is important to consider the Fåhræus–Lindqvist (FL) effect that states the dependence of apparent blood viscosity on the holding vessel inner diameter for micron-sized vessels (Pries et al., 1992). Specifically, the apparent blood viscosity reduces with diameter reduction for microvessels with diameters below 300 µm before the trend inverts to sharp increases in viscosity with further diameter reduction at capillary scales (3–10 µm). The inversion point for this biphasic trend is dependent on the vessel hematocrit but for most of the diameter range below 300 µm, the blood viscosity reduces in response to vessel diameter reduction (Supplementary Figure S2). Mechanistically, the FL effect is due to the increasing prominence of the hydrodynamics of the cell-free plasma layer (CFL) as vessel sizes reduce. The CFL provides a lubricating buffer for RBC-flow in the center of the lumen and progressively increases in effective lubrication as its relative size increases as vessels reduce in diameter (Pries and Secomb, 2005; Fedosov et al., 2010). The FL trend inversion occurs when further lumen reductions in capillary-sized vessels diminishes the CFL to the extent that flowing RBCs now experience higher frequency of frictional contact with vessel walls, thus heightening apparent blood viscosity. To the best of our knowledge no study has measured zebrafish blood viscosity using microfluidic devices with geometries in 5–40 µm range relevant to the zebrafish trunk vascular network. Only the empirical observations of mammalian blood behavior in glass microcapillary tubes such as ones summarized by Pries’ model (Pries et al., 1992) provide a reference for the blood viscosity estimation in small microvessels. Thus, it should be noted that the commonly reported zebrafish blood viscosity has been measured at macroscopic scales. For example, calculation of WSS in the zebrafish heart has been presented with assumptions of a macroscopic blood viscosity between 0.003–0.005 Pa s in the work by (Jamison et al., 2013) and this is perfectly valid due to the size of the heart chamber exceeding 100 µm. When measured using a device with 240 µm width, the macroscopic scale blood viscosity was reported to be 4.2 cP (Lee et al., 2017) at 0.4 hematocrit ( $H_{t}$ ). Pries’ model employed in our study corresponded well with this data and predicted a similar macroscopic scale blood viscosity of 0.0038 Pa s (3.8 cP) in vessels of 240 µm diameter if we assume their reported plasma viscosity of 0.00146 Pa s in our calculations (Supplementary Figure S2Aii). Notably, our estimated apparent blood viscosity in the zebrafish trunk network is lower than those reported in literature because of the pronounced FL effect at small diameter ranges—the model predicts a significantly lower blood viscosity of 0.0022 Pa s (2.2 cP) at 10 um (see $H_{t}$ = 0.4 in Supplementary Figure S2Bii) for the same hematocrit level due to the FL effect.

We would like to provide some discussion on alternative methods for obtaining WSS that exist in comparison to our coarse-grained approach. In formulating Eq. 12 for the evaluation of WSS, we have made the assumption that blood flow velocity profile in the lumen cross section is parabolic. Contrary to this, the lumen velocity profile in zebrafish vessels under physiological hematocrit has been reported (Choi et al., 2017) to be blunted and non-parabolic. Two-phase blood flow models can account for non-parabolic velocity profiles (Namgung et al., 2013) but application of such models require calculation of the mass and momentum balance through iterative optimization of the best-fit cross-sectional velocity profile model in the vessel lumen, thus making their usage complicated. Applying this technique, our WSS evaluation can double from current calculations in vessel regions with high hematocrit. Given that this error is systematic, it is expected to only affect the absolute values of our WSS calculations but not affect the spatial trends discussed here. Hence, our significant findings are not diminished by this methodology limitation. Instead of theoretically calculating the velocity profile, another approach to calculating WSS is by directly measuring the RBC velocity distribution radially along the lumen cross-section. In this approach, the edge velocity of the moving RBC core is identified and by assuming the plasma velocity to decay linearly from the edge velocity at the fringe of the RBC core to zero at the lumen wall, the WSS is estimated from the plasma shear rate ( $\gamma_{CFL}$ ) in the CFL and the CFL thickness ( $\delta_{CFL}$ ) (Namgung et al., 2011; Choi et al., 2017):

$$WSS=\eta_{plasma}\gamma_{CFL}\;where\;\;\gamma_{CFL}=U_{core-edge}/\delta_{CFL}$$ (19)

As shown in Eq. 19, the evaluation of $\gamma_{CFL}$ requires measurement of the velocity at the edge of the RBC core flow ( $U_{core-edge}$ ). Our peak-filtered RBCs velocity $V_{peak}$ is not the same as $U_{core-edge}$ . To use this method, we require high resolution particle image velocimetry (PIV) of the velocity profile across the lumen cross-section to determine $U_{core-edge}$ which is not possible when the tracking particles such as RBCs are of comparable sizes to the vessel lumen diameter. Thus, this technique is incompatible with the evaluation of WSS in small vessels observed in the zebrafish trunk network. Lastly, in terms of quantitative accuracy, WSS estimation is best assessed by combining computational fluid dynamics (CFD) simulations with high resolution imaging of blood velocities and lumen geometry (Vedula et al., 2017; Karthik et al., 2018; Roustaei et al., 2022). WSS calculation requires two key elements for a good estimation, the first is the accurate calculation of the wall shear rate (WSR) and the second is the accurate evaluation of the local blood viscosity near the wall. The WSR calculation requires evaluation of the velocity gradients near the wall and this can be provided by micro PIV with high resolution imaging that reconstructs the velocity field in the lumen space. Blood viscosity estimation often employs either empirical formulations as presented in our current approach or a measured macro viscosity of blood that does not recapitulate the modulation of viscosity by RBC biophysics at microvascular scales. CFD performed in tandem with experimentally obtained velocity information can recapitulate blood viscosity with correct modeling of the blood cell components (Fedosov et al., 2011; Vedula et al., 2017). However, CFD is costly in both the model preparation and computing time consumed by the numerical simulation. This makes CFD far from being high-throughput. Using CFD to study qualitative trends is inefficient and should be reserved for geometry-specific investigations of hemodynamic interactions between flow forces and vessel morphogenesis. In summary, although our WSS model is relatively coarse-grained in comparison with high-resolution imaging and modeling techniques, our approach considers sufficiently represents biophysics of multiphase blood flow at micron scales as described by the FL effect while maintaining the high-throughput sensibility that facilitates qualitative trend analyses.

Our analyses revealed two interesting hemodynamic features during the growth and development of the zebrafish. The first is developmental changes in the systolic WSS ( $WSS_{peak}$ ), which peaks at 3 dpf and decreases thereafter. The second is the existence of an anterior-to-posterior $WSS_{peak}$ gradient that although decreases in magnitude, is maintained in all vessel types. Notably, WSS decreases from anterior to posterior in the DA/CA while it decreases from posterior to anterior in the PCV/CV. The observed higher $WSS_{peak\;}$ and steeper AP gradient in $WSS_{peak}\;$ at 2–3 dpf coincide with the period of active vessel remodeling such as pruning and vessel constriction, processes that require blood flow-dependent EC rearrangement within the vascular network and EC shape changes (Chen et al., 2012; Kochhan et al., 2013; Lenard et al., 2015; Sugden et al., 2017). Later, $WSS_{peak}$ decreases to below 0.3 Pa in all vessel types analyzed and AP gradients in $WSS_{peak}$ appear to asymptote towards zero gradient levels by 6 dpf. The observation that $WSS_{peak}$ lowers below 0.3 Pa and PSR below 30 s^−1^ in all vessels indicates that the absolute levels of hemodynamic quantities are becoming more homogeneous as the zebrafish develops, possibly reaching a state of homeostasis, especially in the caudal regions of the zebrafish. It is unclear if ECs are capable of directly sensing or responding to WSS gradients and if so, how the interplay between WSS gradients and absolute WSS levels affect the EC response to these observed trends. Further experiments will be required to investigate the relationship between the weakening of $WSS_{peak}\;$ gradient and EC behaviors, in particular cell rearrangements, at later stages of vessel morphogenesis when ECs are expected to reach a state of quiescence.

It has been reported that the endothelial junctional mechanosensory complex regulate vascular diameters to maintain a shear stress setpoint (Baeyens et al., 2015) by influencing EC behaviors. Studies have explored the role of VEGF receptors in modulating the mechanosensory complexes (Coon et al., 2015) but few studies have provided empirical data for crafting mechanistic understanding of the vascular morphogenesis and EC rearrangement trends in accordance with fluid shear stress levels as described in the theoretical discussions of this topic by Roux and colleagues (Roux et al., 2020). We believe that our high throughput approach to estimating WSS distribution in developing zebrafish can provide empirical data to further explore the fluid shear stress setpoint concept from an empirical perspective.

In conclusion, our current study represents the first in-depth comprehensive analysis of hemodynamics during the development of the zebrafish. Using a high-throughput semi-automated imaging and analyses protocol we presented a new finding that anterior-to-posterior (AP) gradients of hemodynamic quantities exist in the zebrafish trunk vasculature and evolve with development. We believe this previously unreported developmental trend can increment the current understanding of zebrafish vascular development and physiology with follow up studies that elucidate the correlation between EC rearrangements and the developmental changes in AP gradients. Furthermore, our method can be applied to investigate systemic changes in hemodynamics in zebrafish models of vessel malformations under pathological conditions and can be of significant relevance to diverse research fields such as tumor angiogenesis and organoid vascularization research.

## Data Availability

The original contributions presented in the study are included in the article/Supplementary Material, further inquiries can be directed to the corresponding author
