# Supplementary material for: High-Throughput Imaging of Blood Flow Reveals Developmental Changes in Distribution Patterns of Hemodynamic Quantities in Developing Zebrafish
Source: Front Physiol. 2022 Jun 20;13:881929. doi: 10.3389/fphys.2022.881929 (PMC9251365; doi:10.3389/fphys.2022.881929)
Supplement: Supplementary file 5 [file DataSheet1.pdf]

Supplemental Data: Figures, Tables and Videos

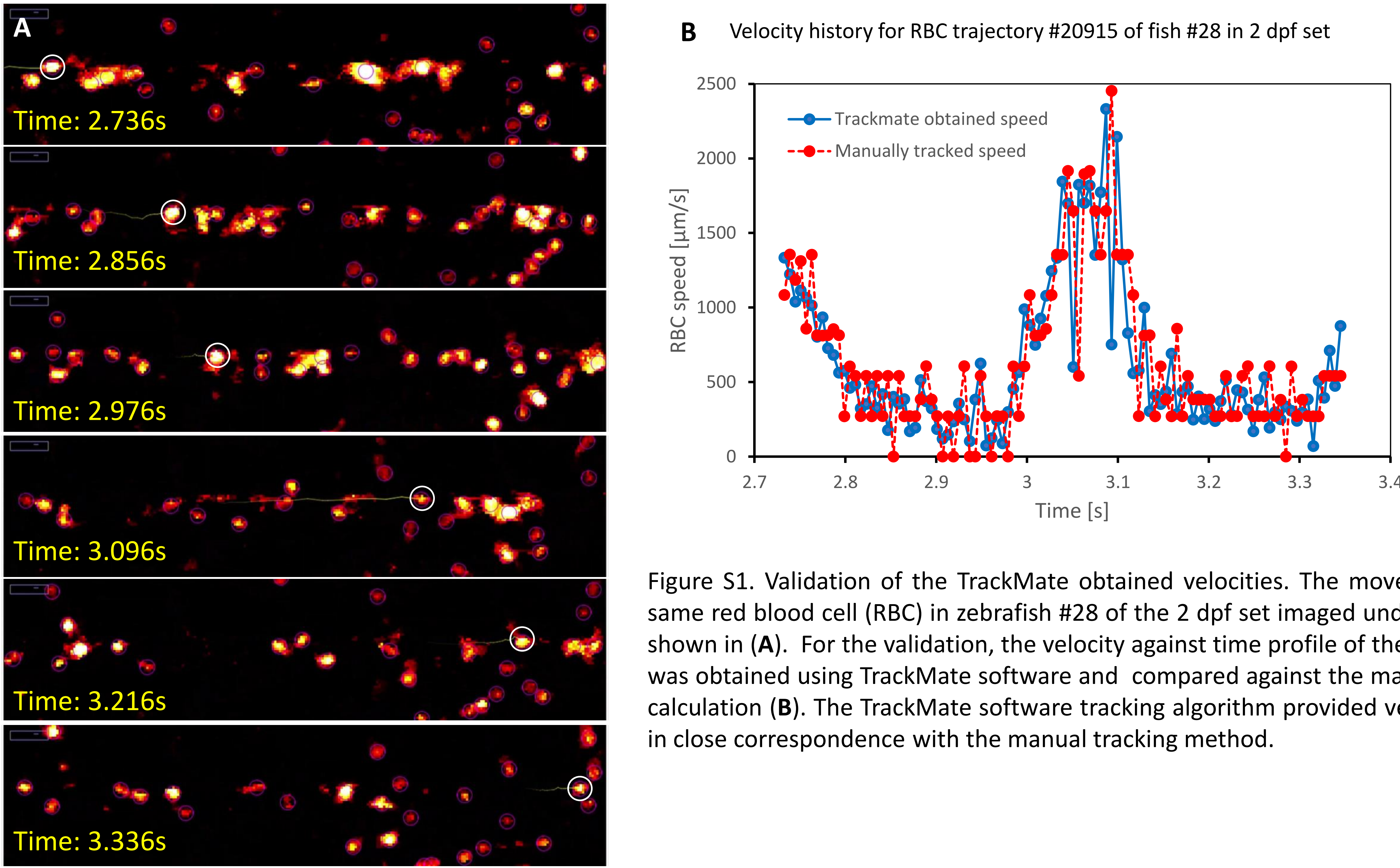

Figure S1. Validation of the TrackMate obtained velocities. The movement of the same red blood cell (RBC) in zebrafish #28 of the 2 dpf set imaged under 180 fps is shown in (**A**). For the validation, the velocity against time profile of the tracked RBC was obtained using TrackMate software and compared against the manual tracking calculation (**B**). The TrackMate software tracking algorithm provided velocity values in close correspondence with the manual tracking method.

Figure S2. Fåhræus–Lindqvist (FL) effect model for microvessels, where the blood viscosity is dependent on plasma viscosity, lumen tube hematocrit (Ht) and vessel lumen diameter; these calculations are performed using equations 13 to 17 of the main article: Biphasic viscosity against vessel diameter behavior for blood flow in microvessels with a comparatively wide range of diameters over which viscosity reduces with diameter reduction (**A** and **B**) and a trend reversal at capillary diameters **(C).** Examples of the FL effect model is showed on whole blood with plasma viscosity of 0.0012 Pa·s (i) and 0.00146 Pa·s (ii).

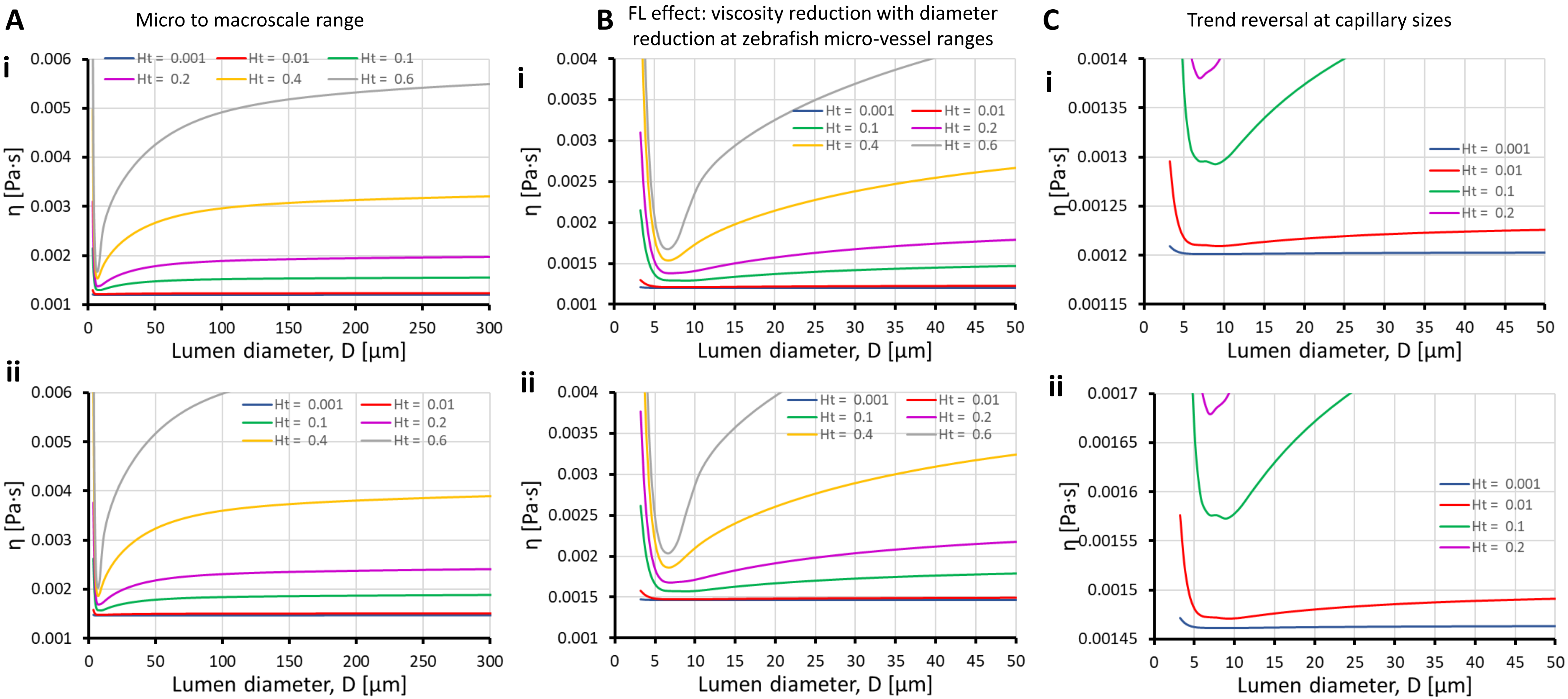

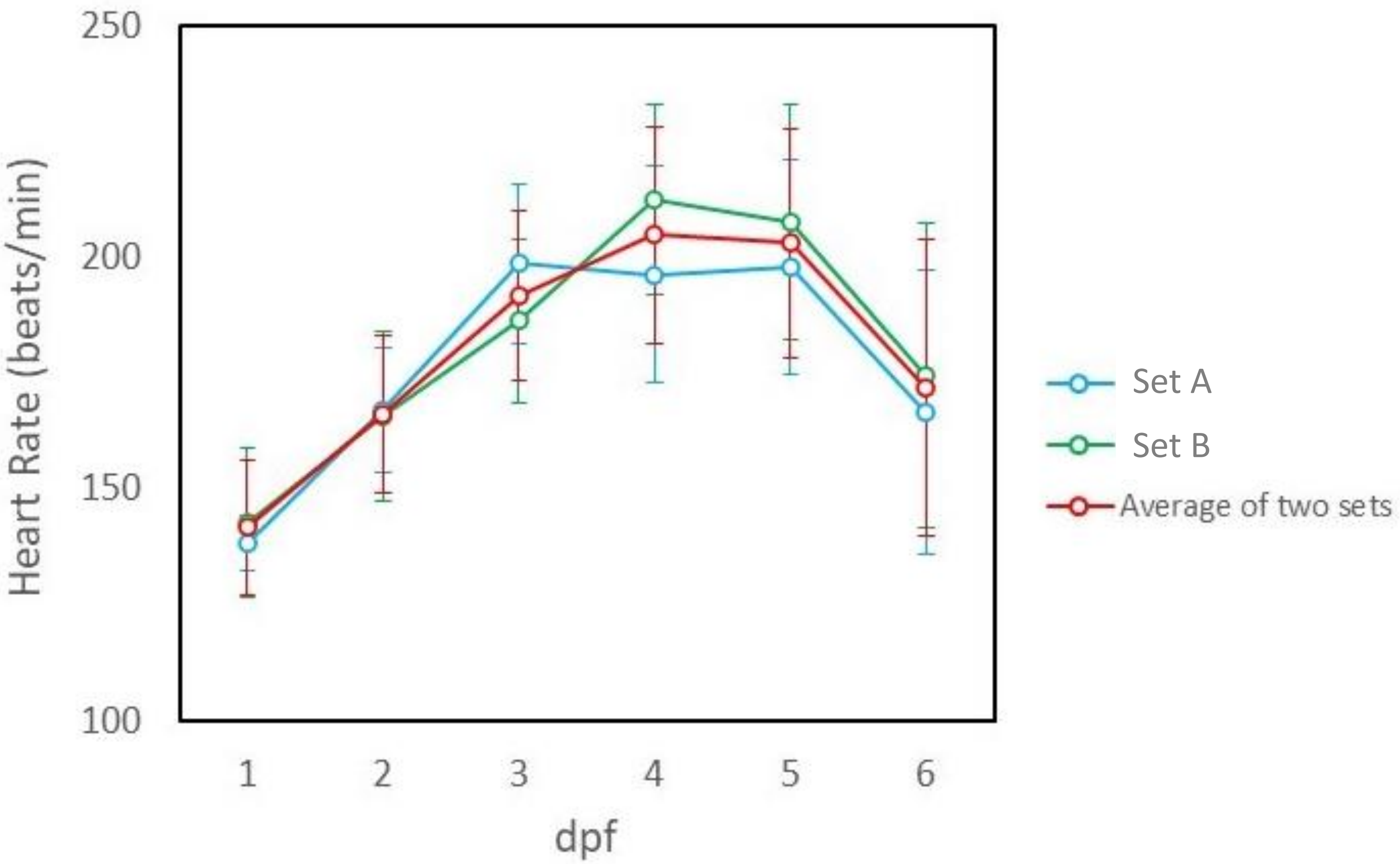

Figure S3. Zebrafish heart rate across development for two different experiment sets. Circle points indicate the mean level heartbeat, and whisker bars represent the standard deviation ranges. Set A (blue): 6, 15, 26, 34, 32 and 15 zebrafish were measured for heart rate at 1, 2, 3, 4, 5 and 6 dpf. Set B (green): 18, 30, 32, 38, 35 and 29 zebrafish were measured for heart rate at 1, 2, 3, 4, 5 and 6 dpf. Set B zebrafish were used for all analysis performed and discussed in the main article. The average of two sets data (red) is a pooling of set A and set B data.

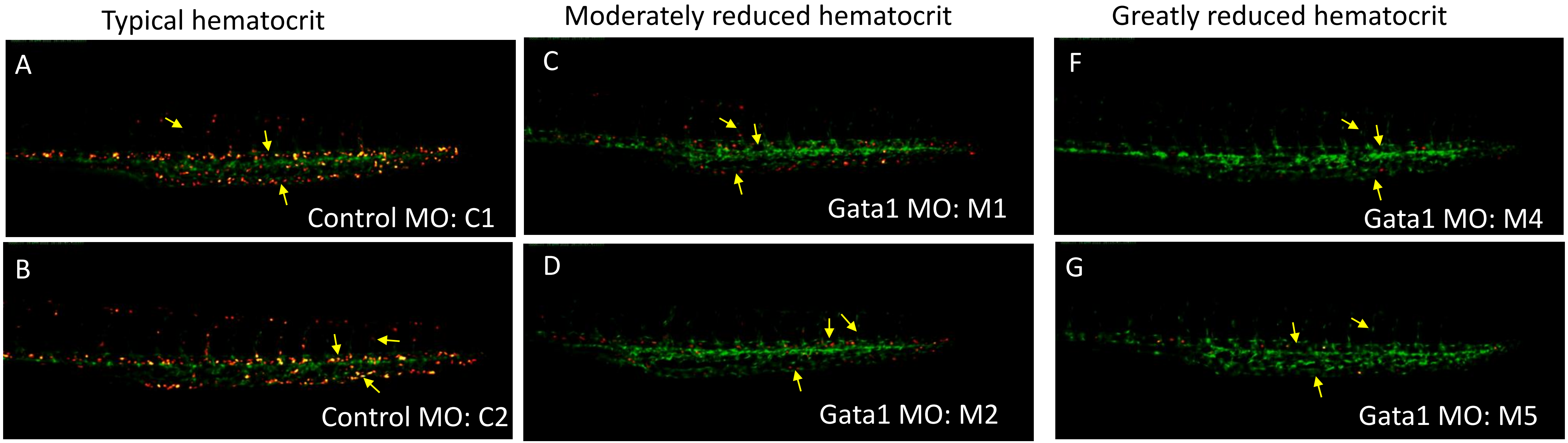

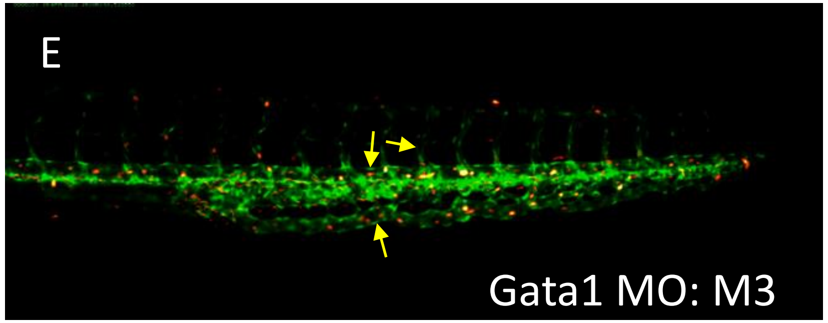

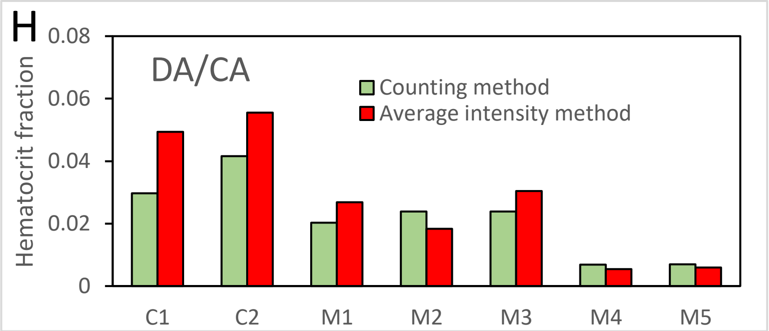

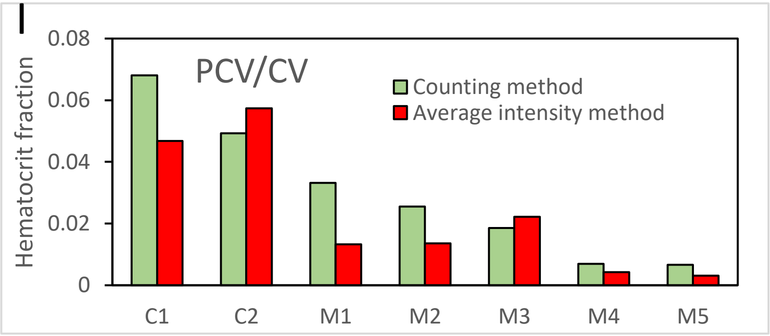

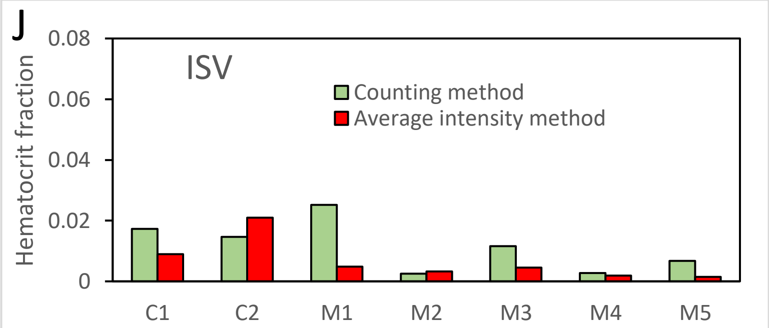

Figure S4. Validation of the hematocrit assessment using RBC reduction in networks by morpholino (MO) treatment targeting Gata1. Varied dosage delivery resulted in three hematocrits levels: normal or typical levels for control MO samples C1 (**A**) and C2 (**B**); moderate reduction in morphants M1 (**C**), M2 (**D**) and M3 (**E**); and greatly reduced levels in M4 (**F**) and M5 (**G**). Red heat color indicates the Gata1:dsRed RBC markers and green indicates the kdrl:EGP lumen EC marker. Yellow arrows denote regions where the diameters and hematocrits were measured for the validation. Videos of circulating RBCs in these respective samples can be seen in **SV 6 – SV12**. The comparison of hematocrit evaluation by counting against the proposed average intensity correlation method is shown for the DA/CA (**H**), PCV/CV (**I**) and ISV (**J**).

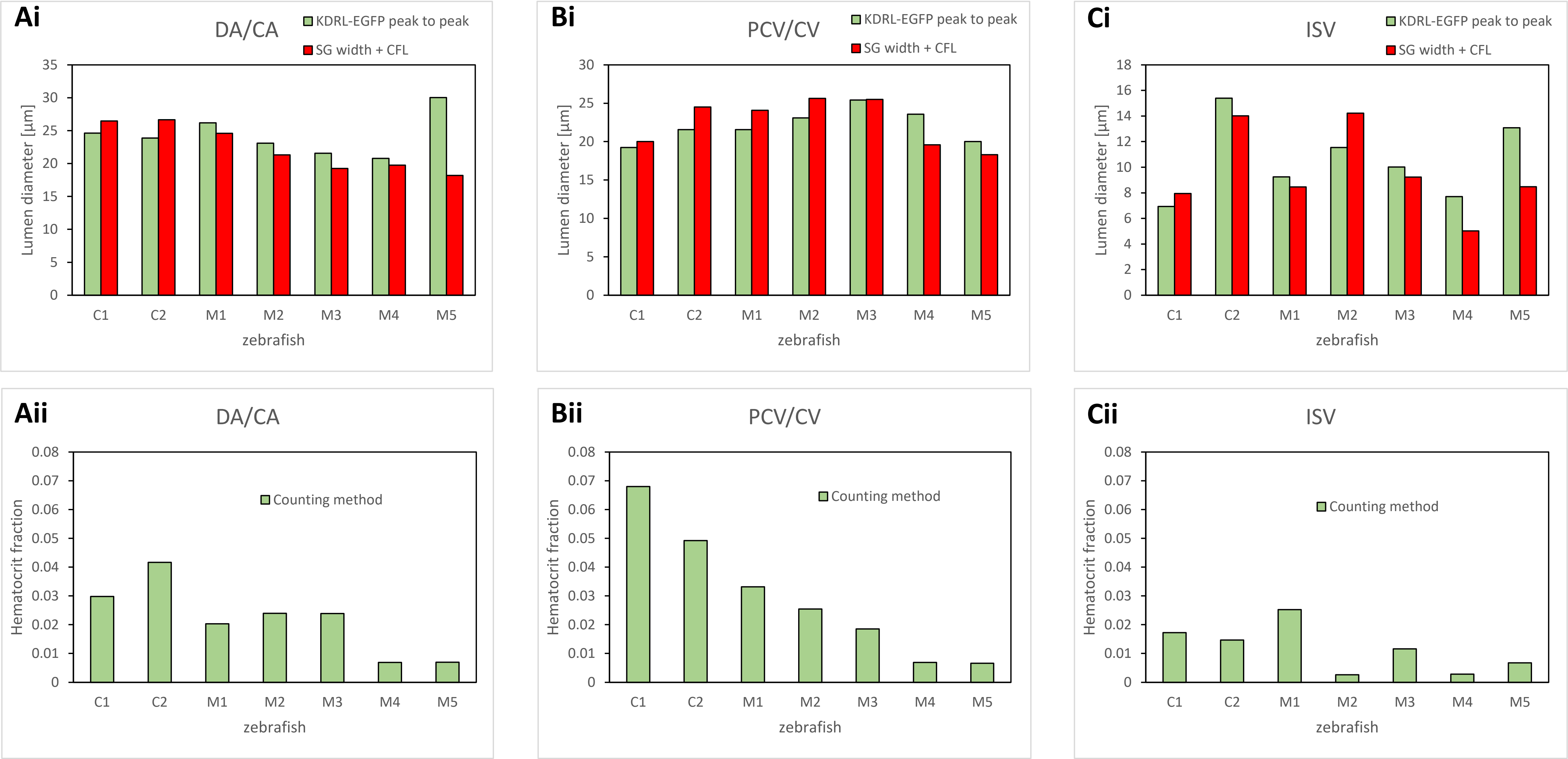

Figure S5. Validation of lumen diameter assessment by comparing method in paper (red) against EC marker peak to peak distance assessment (green) for the DA/CA (**Ai**), PCV/CV (**Bi**) and ISV (**Ci**). Judging against the hematocrit in **Aii**, **Bii** and **Cii**, the discrepancy between the two methods grows as vessel lumen diameters decrease and hematocrits become vanishingly low (hematocrit fraction < 0.01).

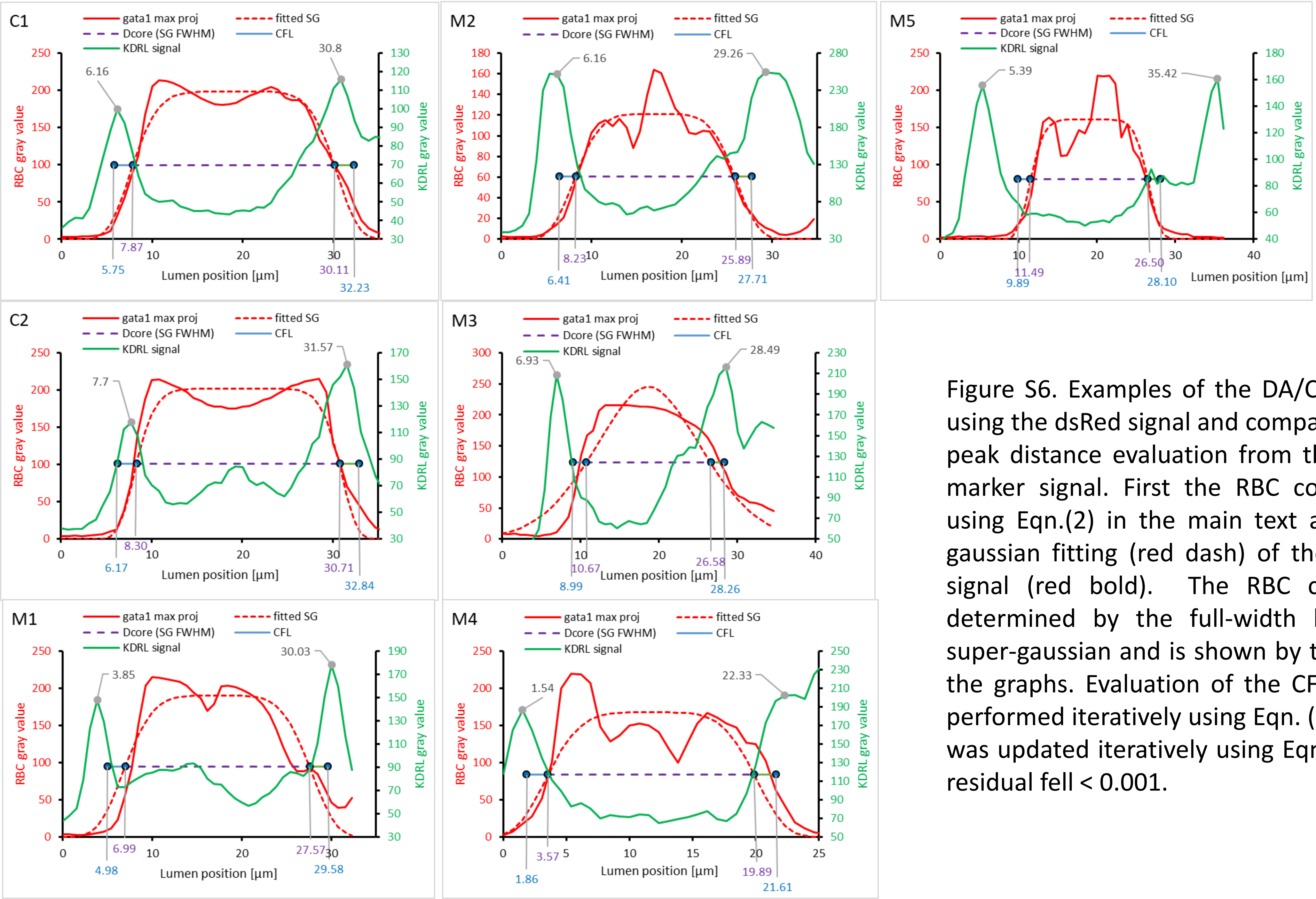

Figure S6. Examples of the DA/CA diameter calculation using the dsRed signal and compared against the peak to peak distance evaluation from the kdrl:EGFP lumen EC marker signal. First the RBC core width is estimated using Eqn.(2) in the main text after applying a super-gaussian fitting (red dash) of the maximum projection signal (red bold). The RBC core width (D_{core}) is determined by the full-width half maximum of the super-gaussian and is shown by the purple dash lines in the graphs. Evaluation of the CFL thickness (δ_{CFL}) was performed iteratively using Eqn. (5) and lumen width (D) was updated iteratively using Eqn. (7) until the iteration residual fell < 0.001.

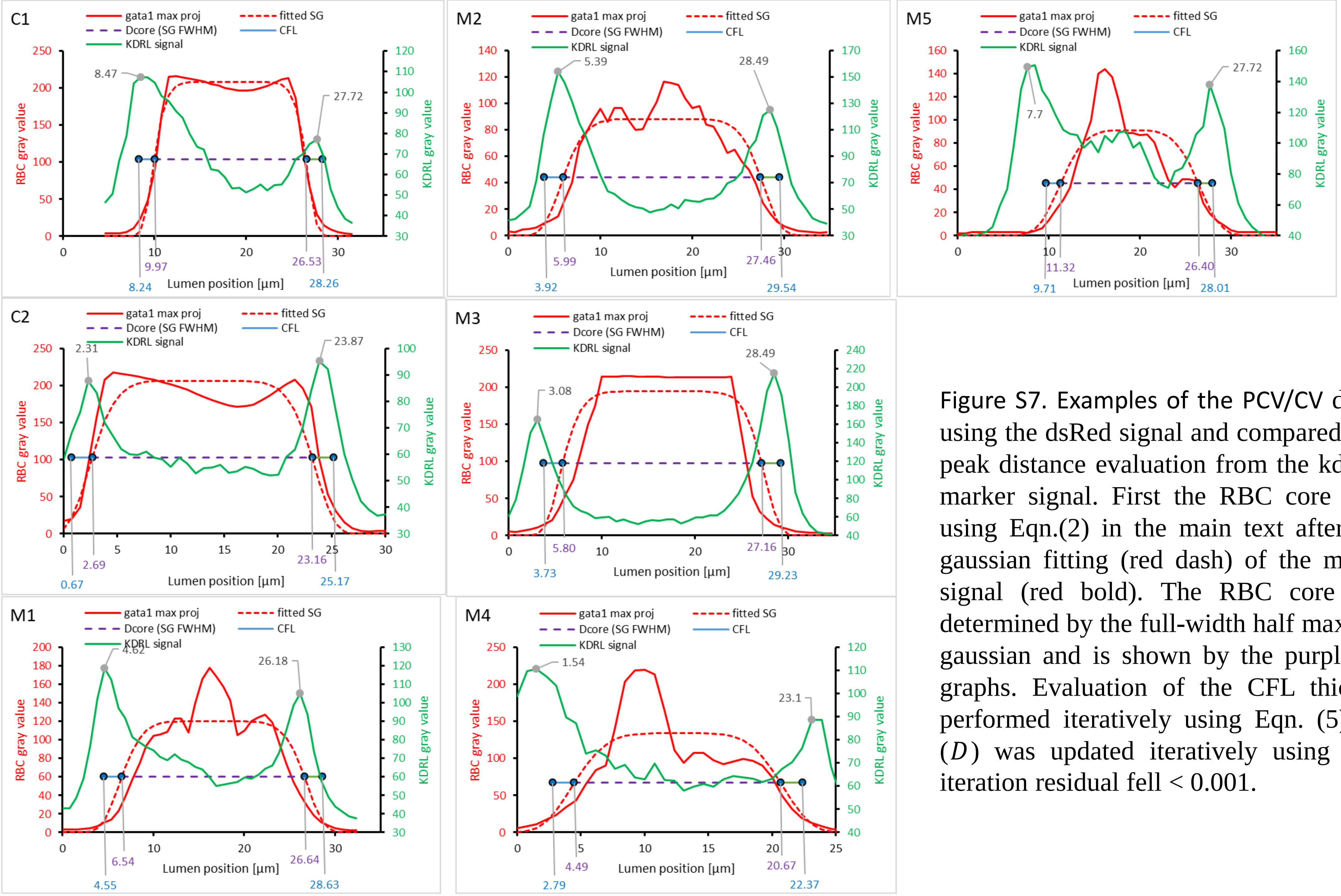

Figure S7. Examples of the PCV/CV diameter calculation using the dsRed signal and compared against the peak to peak distance evaluation from the kdrl:EGFP lumen EC marker signal. First the RBC core width is estimated using Eqn.(2) in the main text after applying a super-gaussian fitting (red dash) of the maximum projection signal (red bold). The RBC core width (D_{core}) is determined by the full-width half maximum of the super-gaussian and is shown by the purple dash lines in the graphs. Evaluation of the CFL thickness (δ_{CFL}) was performed iteratively using Eqn. (5) and lumen width (D) was updated iteratively using Eqn. (7) until the iteration residual fell < 0.001.

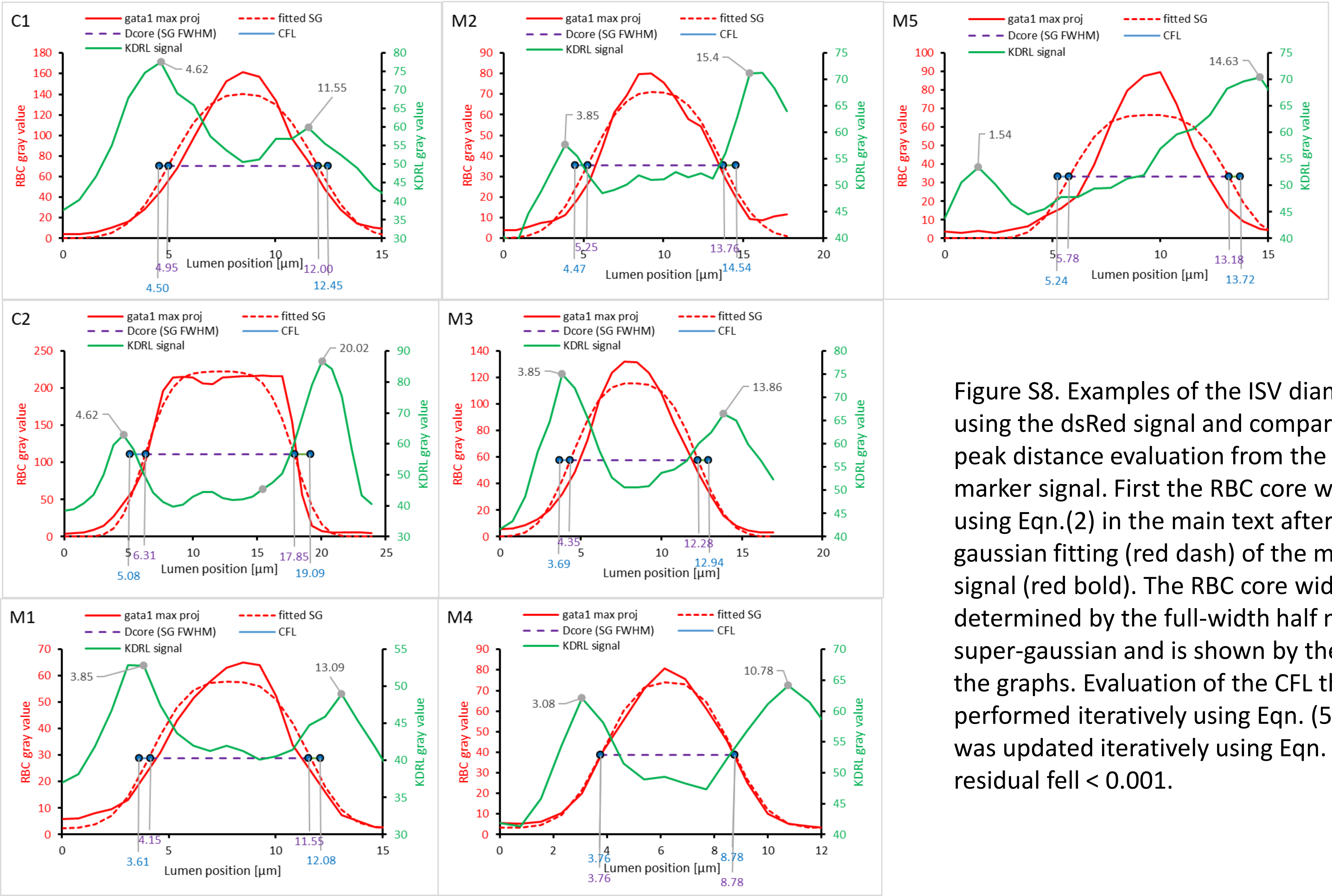

Figure S8. Examples of the ISV diameter calculation using the dsRed signal and compared against the peak to peak distance evaluation from the kdrl:EGFP lumen EC marker signal. First the RBC core width is estimated using Eqn.(2) in the main text after applying a super-gaussian fitting (red dash) of the maximum projection signal (red bold). The RBC core width (D_{core}) is determined by the full-width half maximum of the super-gaussian and is shown by the purple dash lines in the graphs. Evaluation of the CFL thickness (δ_{CFL}) was performed iteratively using Eqn. (5) and lumen width (D) was updated iteratively using Eqn. (7) until the iteration residual fell < 0.001.

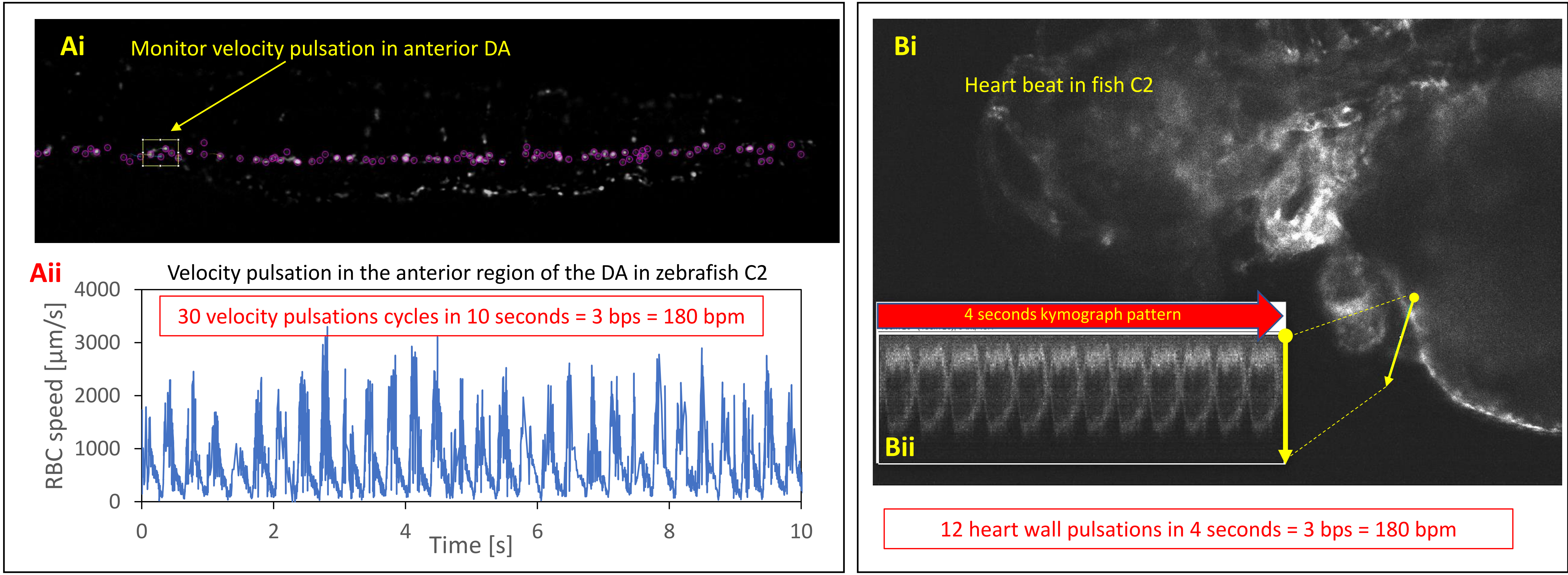

Figure S9. Validation of the direct correspondence between heartbeat rate and velocity pulsation frequency in the DA. Using zebrafish C2 of the validation experiments data set, the velocity pulsation was monitored in the anterior region of the DA in (boxed region in **Ai**) and a pulsation frequency of 180 bpm was obtained based on the observation of 30 pulsation cycles over 10 seconds of velocity sampling (**Aii**). The heartbeat in C2 was obtained by examining the heart wall displacement along the yellow arrow line indicated in **Bi**: Kymograph pattern along this line presented 12 wall pulsation cycles over 4 seconds, which works out to be a heartrate of 180 bpm (**Bii**). Since both methods indicated the same 180 bpm frequency, the assumption that velocity pulsation in the DA gives the heartbeat rate is a valid assumption.

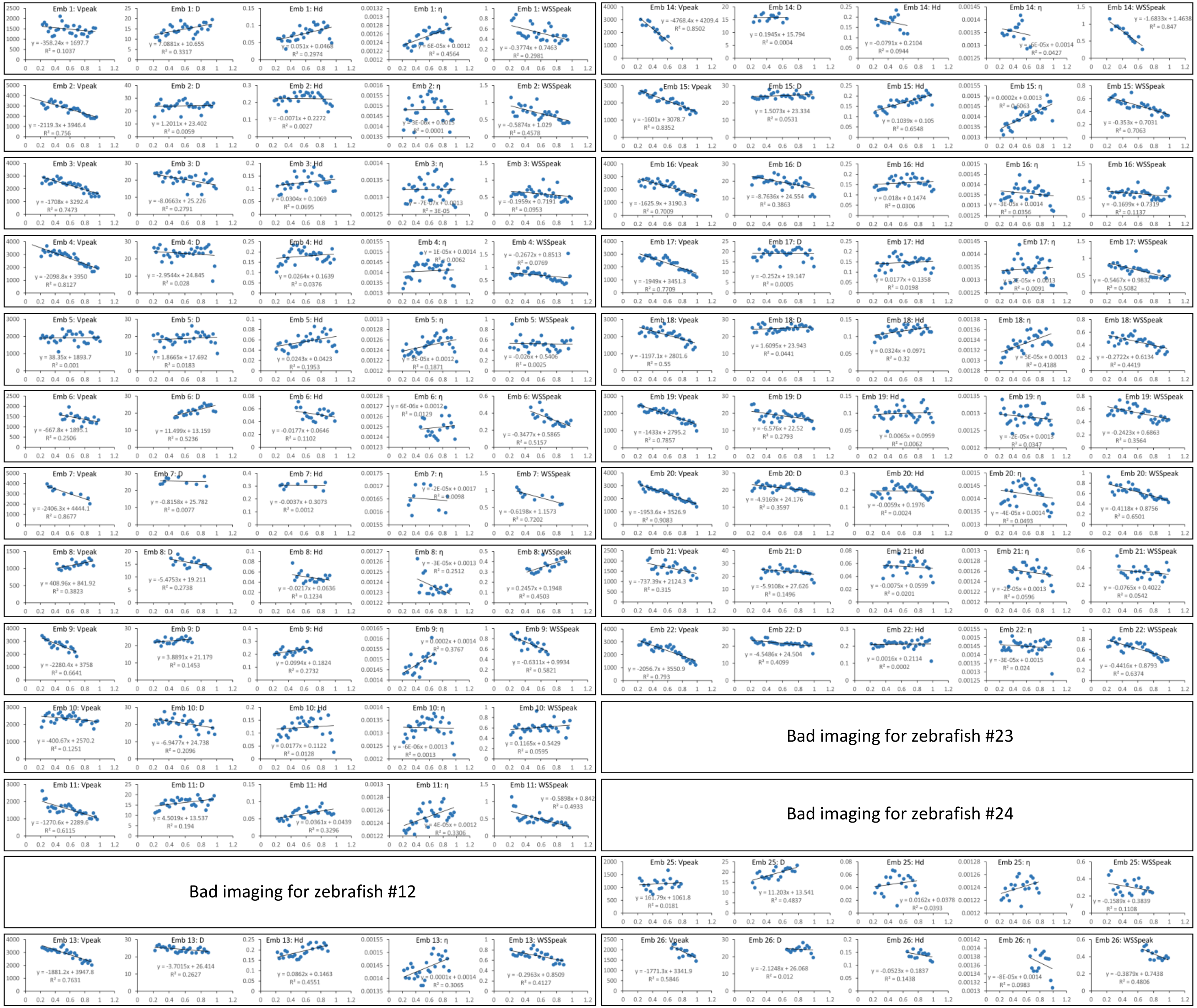

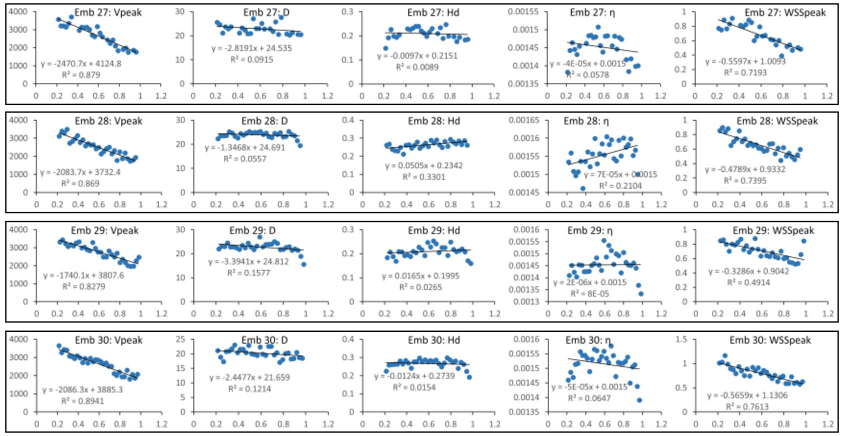

Figure S10. Dorsal aorta/caudal artery trends along the normalized anterior-posterior axis (x-axis of graphs) for systolic velocity Vpeak [µm/s], lumen diameter D [µm], discharge hematocrit fraction Hd [max is 1], apparent blood viscosity η [Pa·s] and systolic wall shear stress WSSpeak [Pa] in 2 dpf zebrafish.

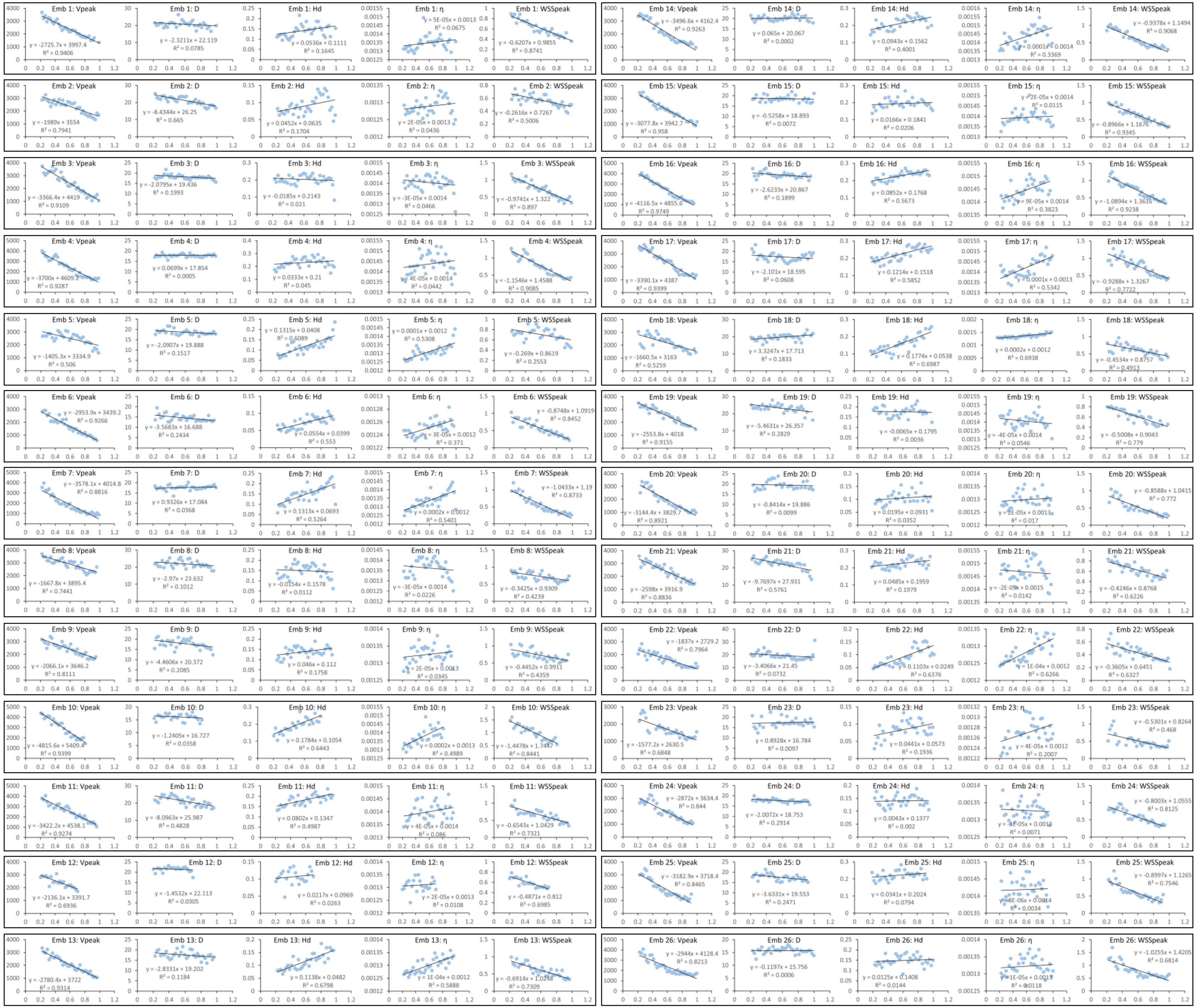

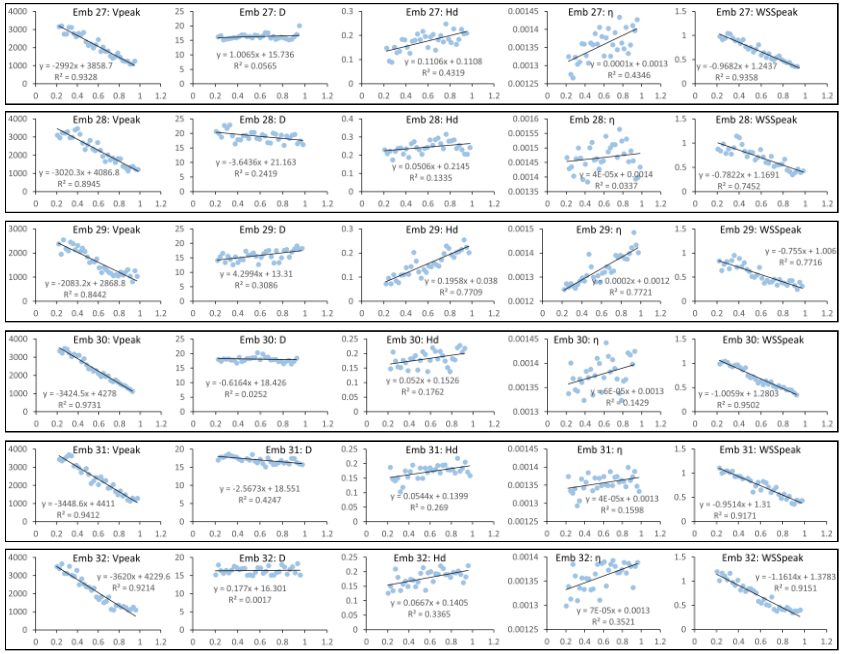

Figure S11. Dorsal aorta/caudal artery trends along the normalized anterior-posterior axis (x-axis of graphs) for systolic velocity Vpeak [µm/s], lumen diameter D [µm], discharge hematocrit fraction Hd [max is 1], apparent blood viscosity η [Pa·s] and systolic wall shear stress WSSpeak [Pa] in 3 dpf zebrafish.

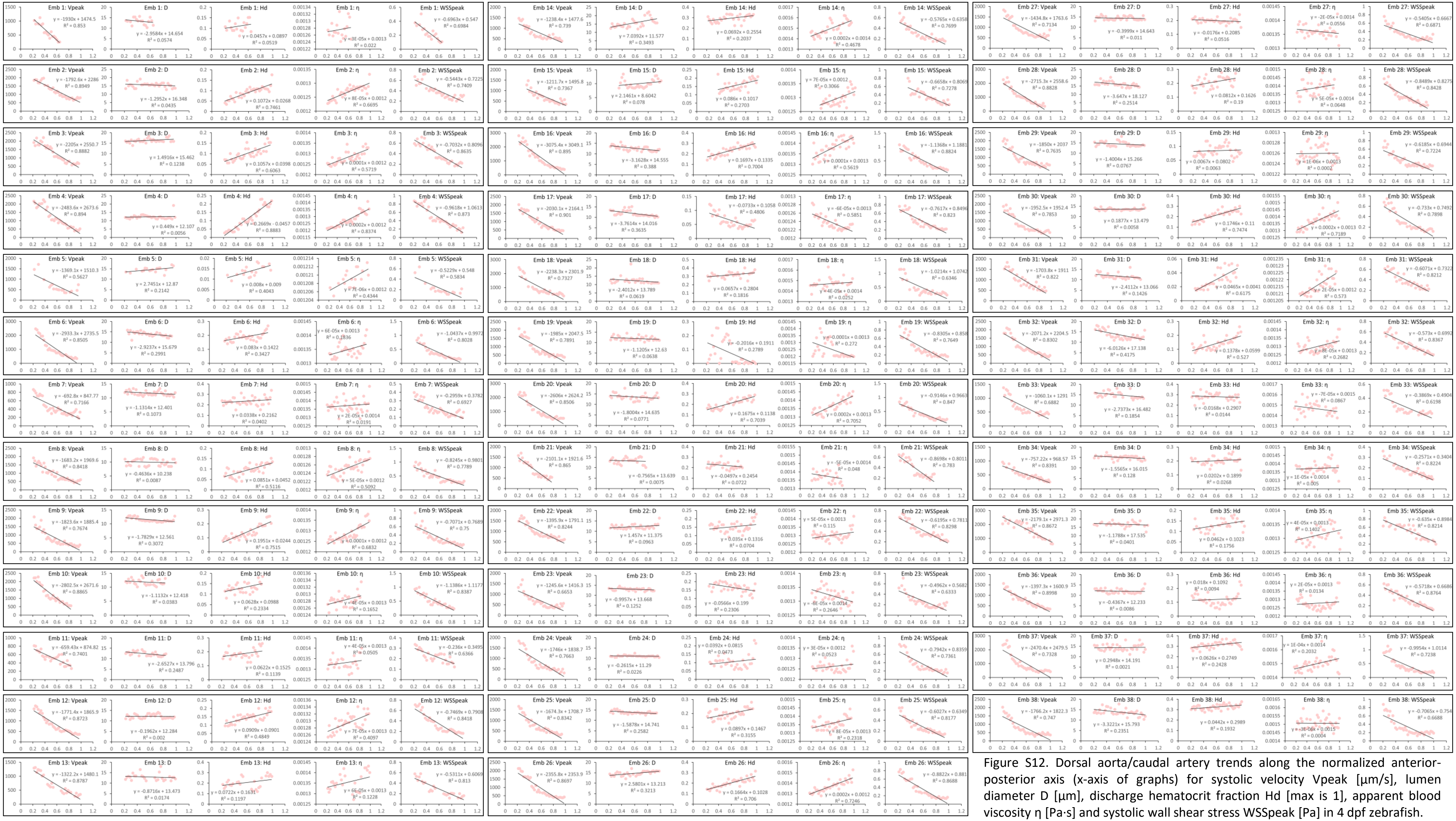

Figure S12. Dorsal aorta/caudal artery trends along the normalized anterior-posterior axis (x-axis of graphs) for systolic velocity Vpeak [μm/s], lumen diameter D [μm], discharge hematocrit fraction Hd [max is 1], apparent blood viscosity η [Pa·s] and systolic wall shear stress WSSpeak [Pa] in 4 dpf zebrafish.

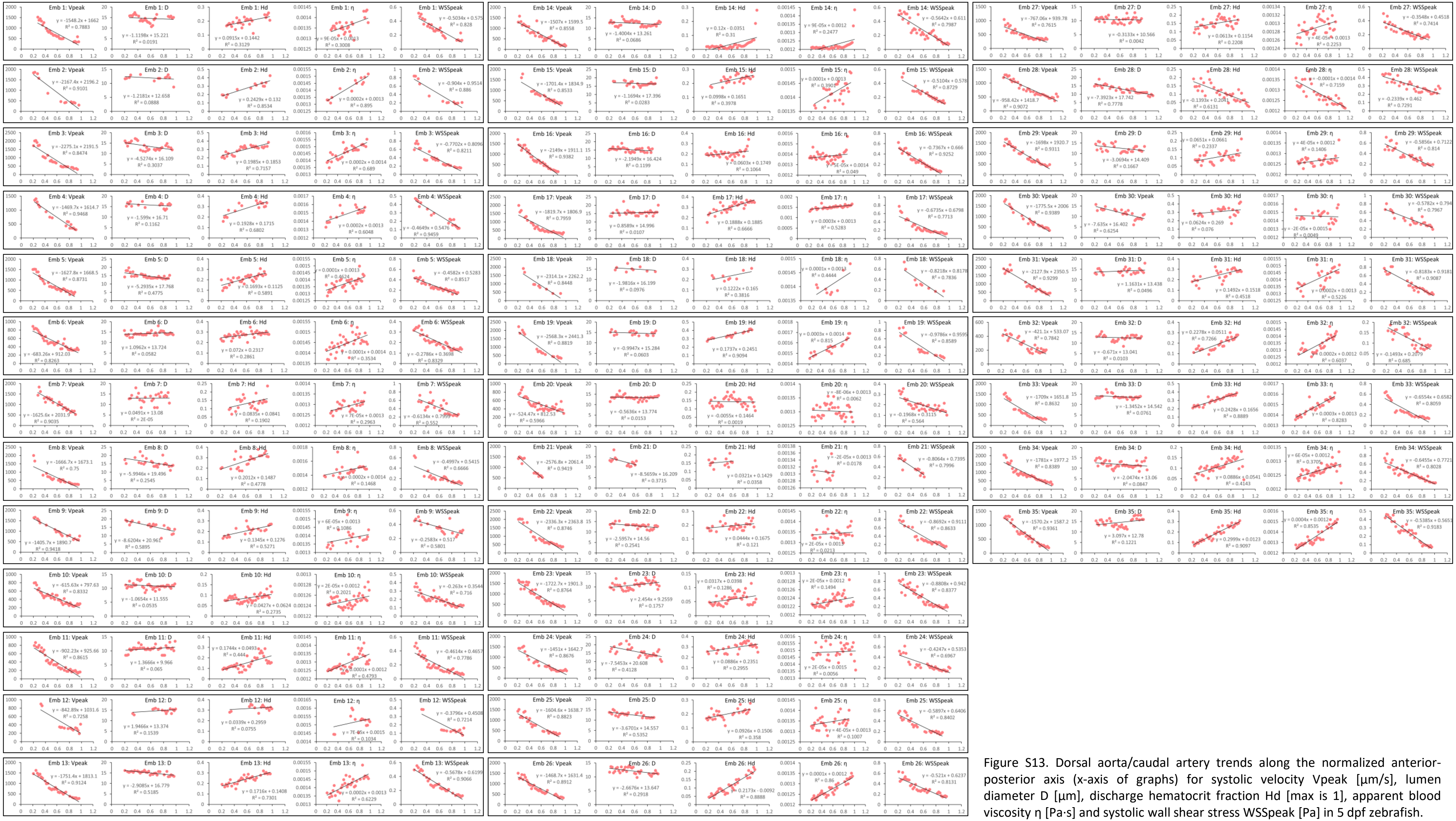

Figure S13. Dorsal aorta/caudal artery trends along the normalized anterior-posterior axis (x-axis of graphs) for systolic velocity Vpeak [µm/s], lumen diameter D [µm], discharge hematocrit fraction Hd [max is 1], apparent blood viscosity η [Pa·s] and systolic wall shear stress WSSpeak [Pa] in 5 dpf zebrafish.

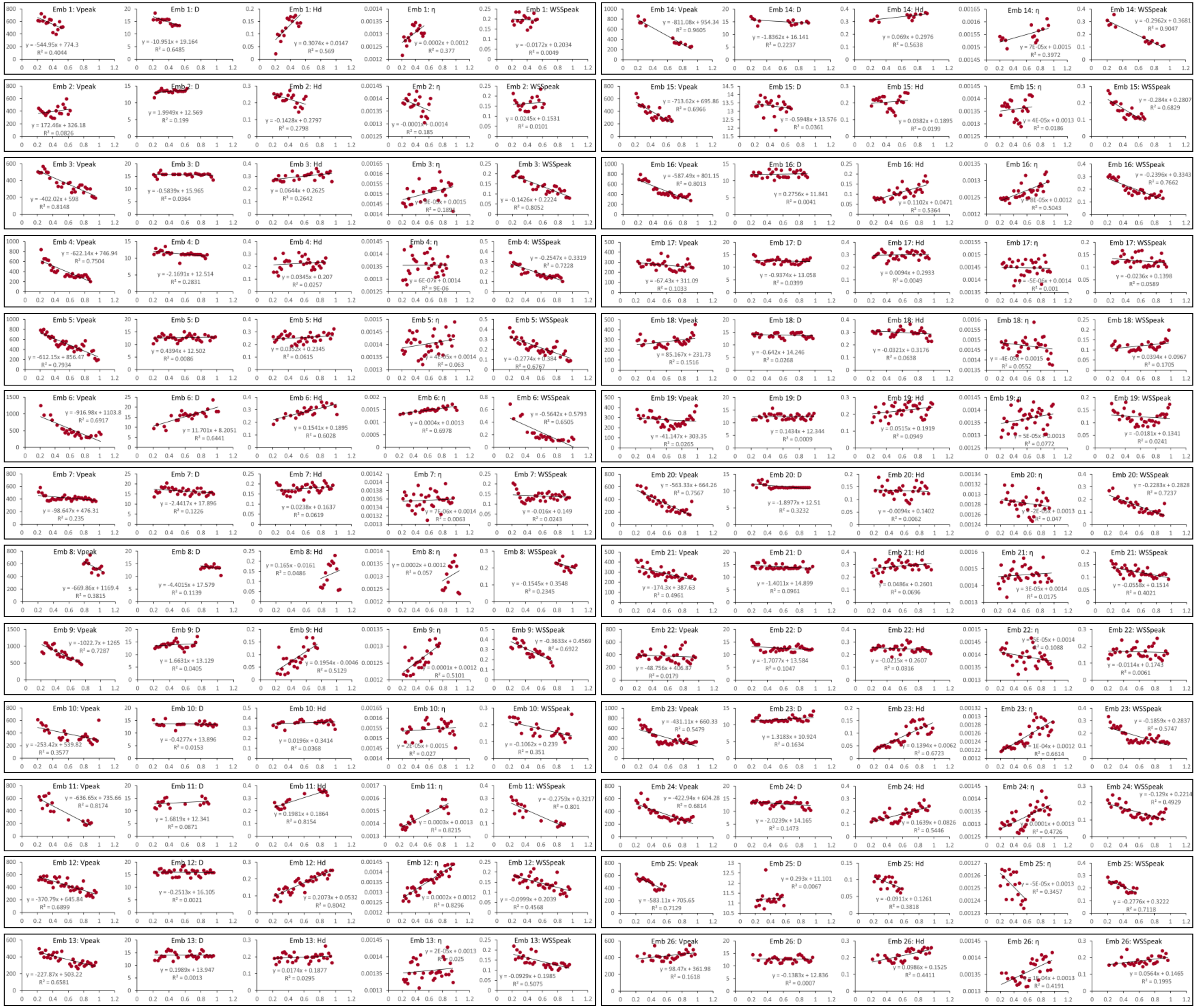
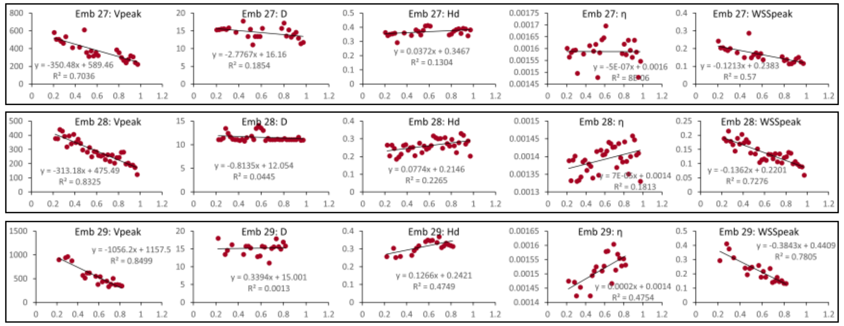

Figure S14. Dorsal aorta/caudal artery trends along the normalized anterior-posterior axis (x-axis of graphs) for systolic velocity Vpeak [μm/s], lumen diameter D [μm], discharge hematocrit fraction Hd [max is 1], apparent blood viscosity η [Pa·s] and systolic wall shear stress WSSpeak [Pa] in 6 dpf zebrafish.

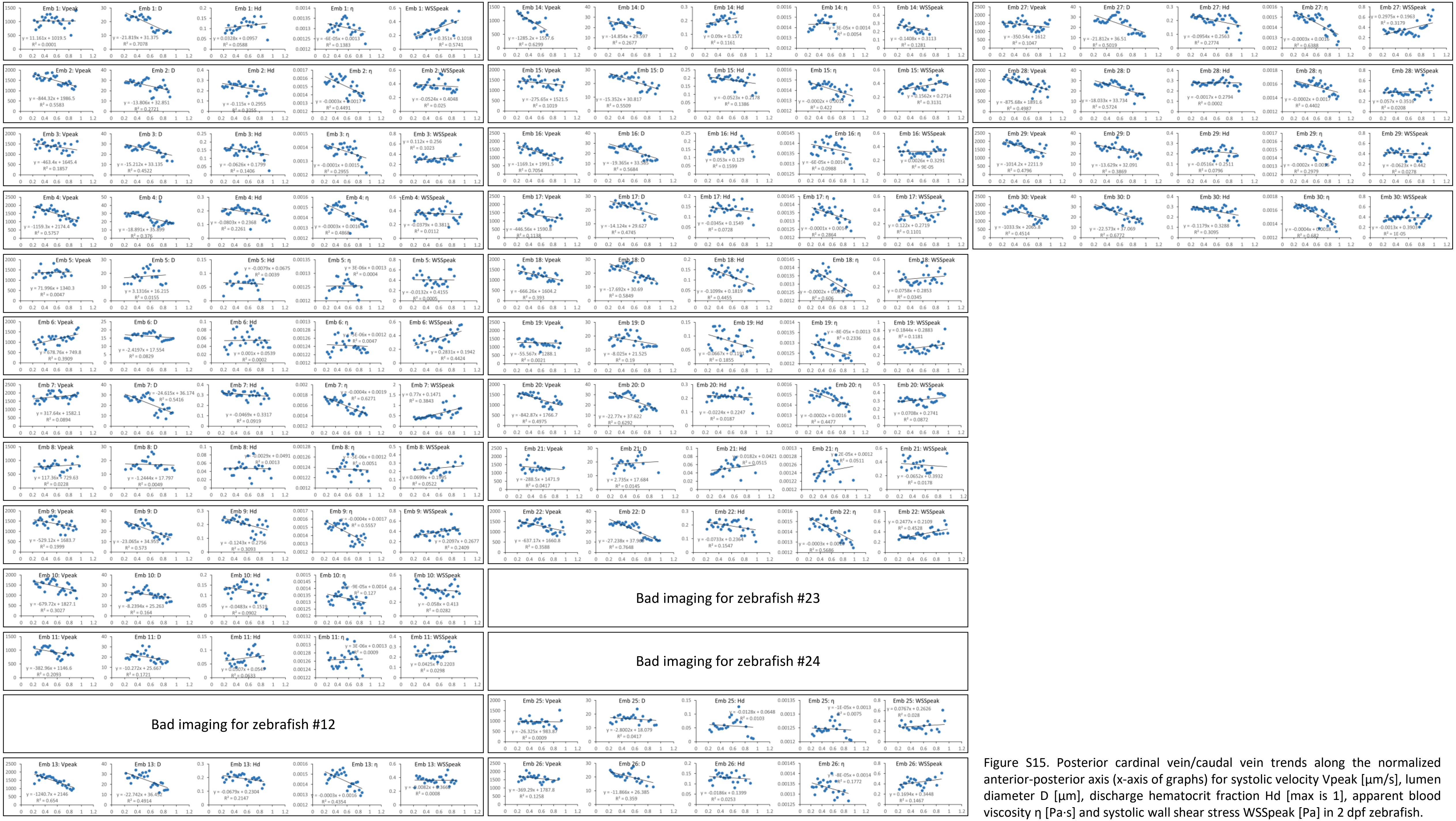

Figure S15. Posterior cardinal vein/caudal vein trends along the normalized anterior-posterior axis (x-axis of graphs) for systolic velocity Vpeak [µm/s], lumen diameter D [µm], discharge hematocrit fraction Hd [max is 1], apparent blood viscosity η [Pa·s] and systolic wall shear stress WSSpeak [Pa] in 2 dpf zebrafish.

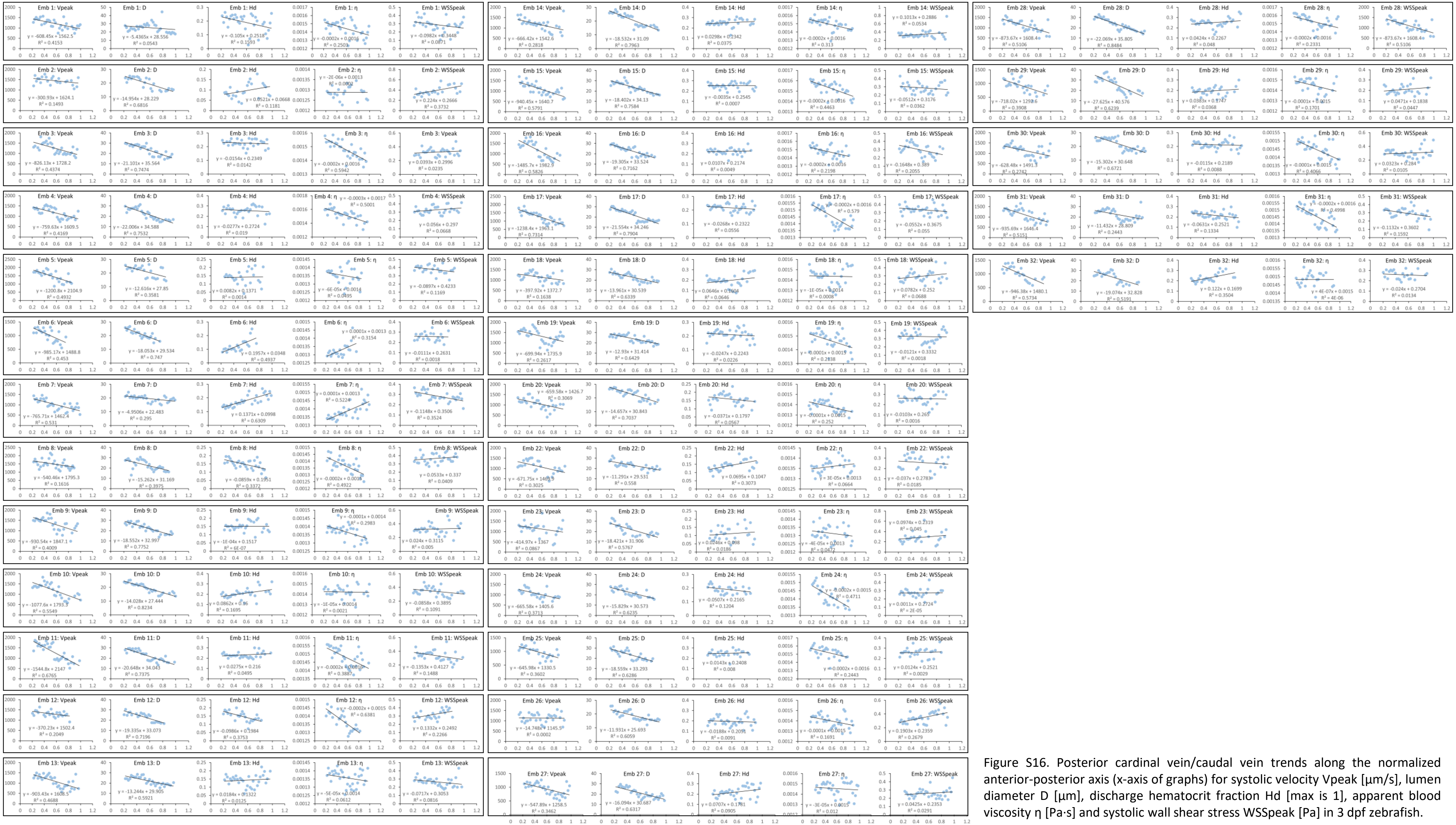

Figure S16. Posterior cardinal vein/caudal vein trends along the normalized anterior-posterior axis (x-axis of graphs) for systolic velocity Vpeak [µm/s], lumen diameter D [µm], discharge hematocrit fraction Hd [max is 1], apparent blood viscosity η [Pa·s] and systolic wall shear stress WSSpeak [Pa] in 3 dpf zebrafish.

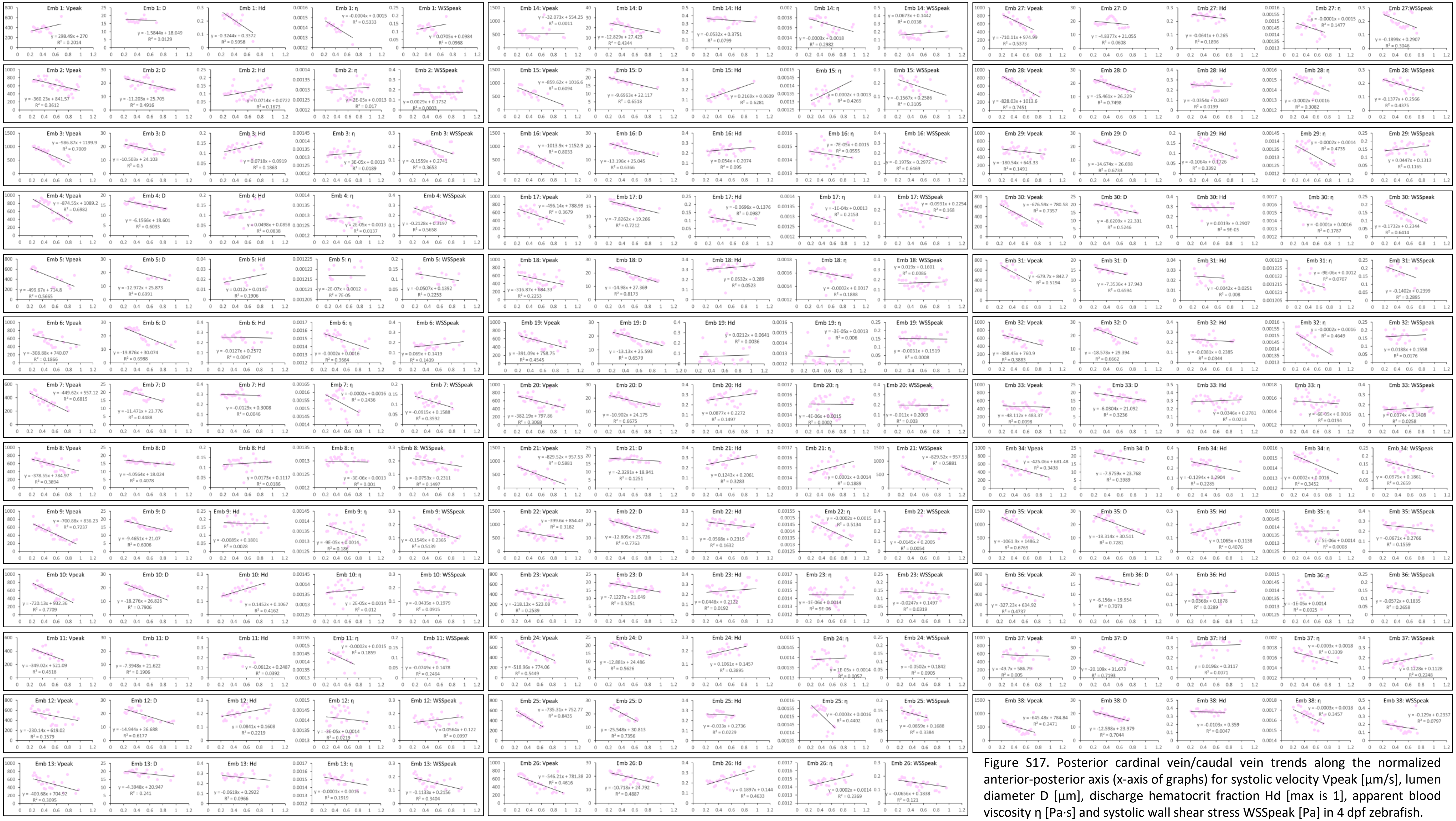

Figure S17. Posterior cardinal vein/caudal vein trends along the normalized anterior-posterior axis (x-axis of graphs) for systolic velocity Vpeak [μm/s], lumen diameter D [μm], discharge hematocrit fraction Hd [max is 1], apparent blood viscosity η [Pa·s] and systolic wall shear stress WSSpeak [Pa] in 4 dpf zebrafish.

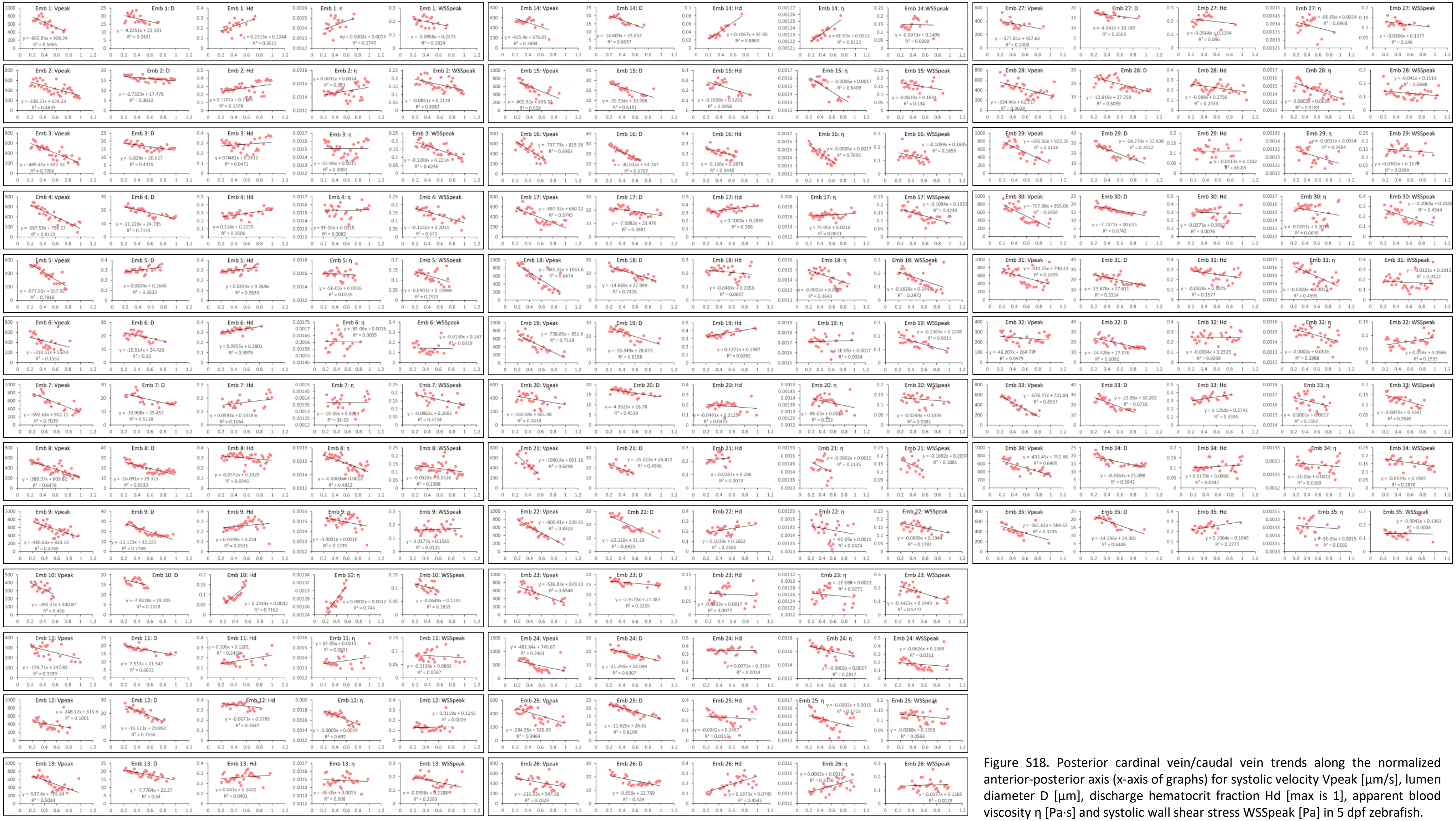

Figure S18. Posterior cardinal vein/caudal vein trends along the normalized anterior-posterior axis (x-axis of graphs) for systolic velocity Vpeak [μm/s], lumen diameter D [μm], discharge hematocrit fraction Hd [max is 1], apparent blood viscosity η [Pa·s] and systolic wall shear stress WSSpeak [Pa] in 5 dpf zebrafish.

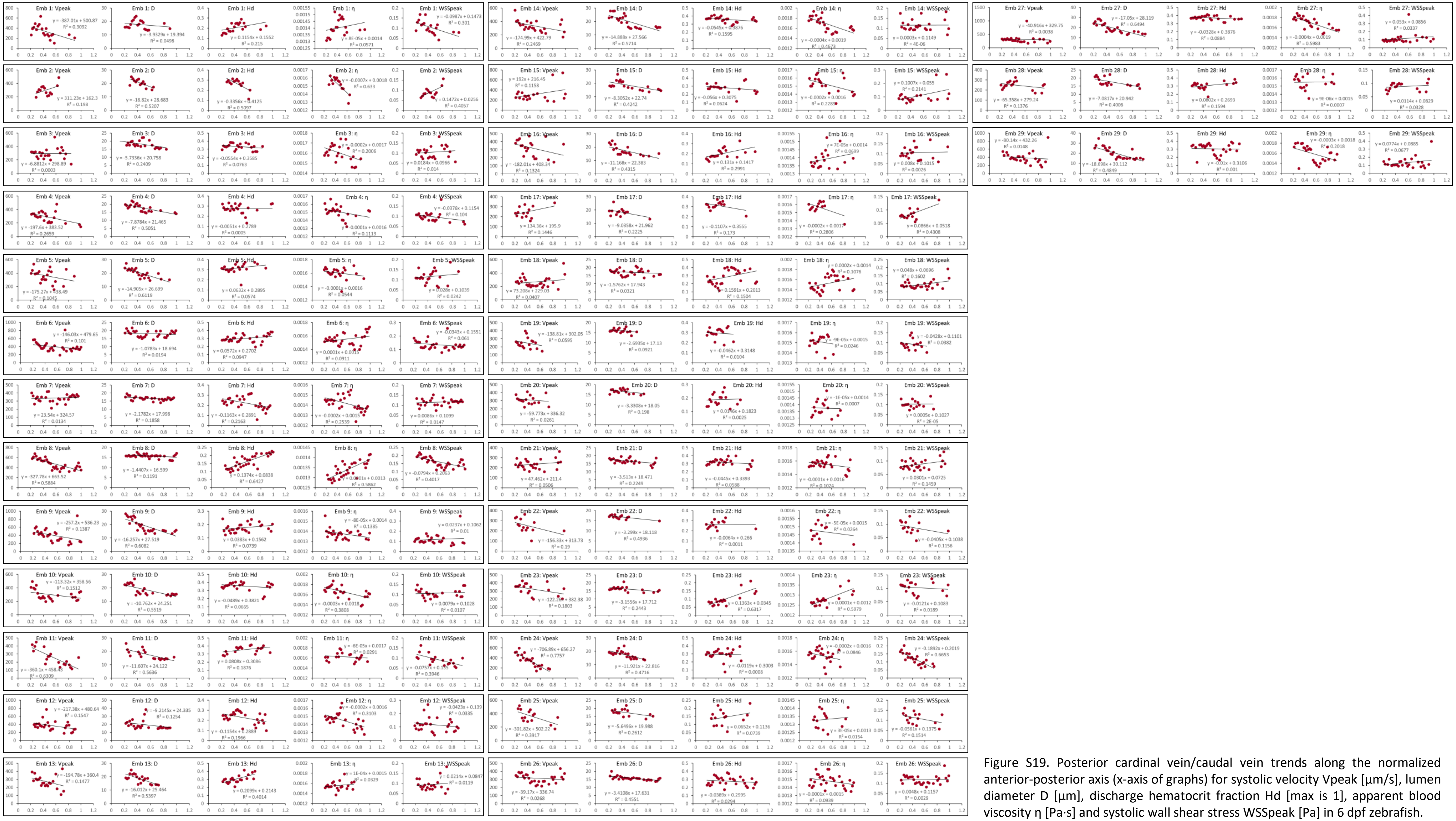

Figure S19. Posterior cardinal vein/caudal vein trends along the normalized anterior-posterior axis (x-axis of graphs) for systolic velocity Vpeak [μm/s], lumen diameter D [μm], discharge hematocrit fraction Hd [max is 1], apparent blood viscosity η [Pa·s] and systolic wall shear stress WSSpeak [Pa] in 6 dpf zebrafish.

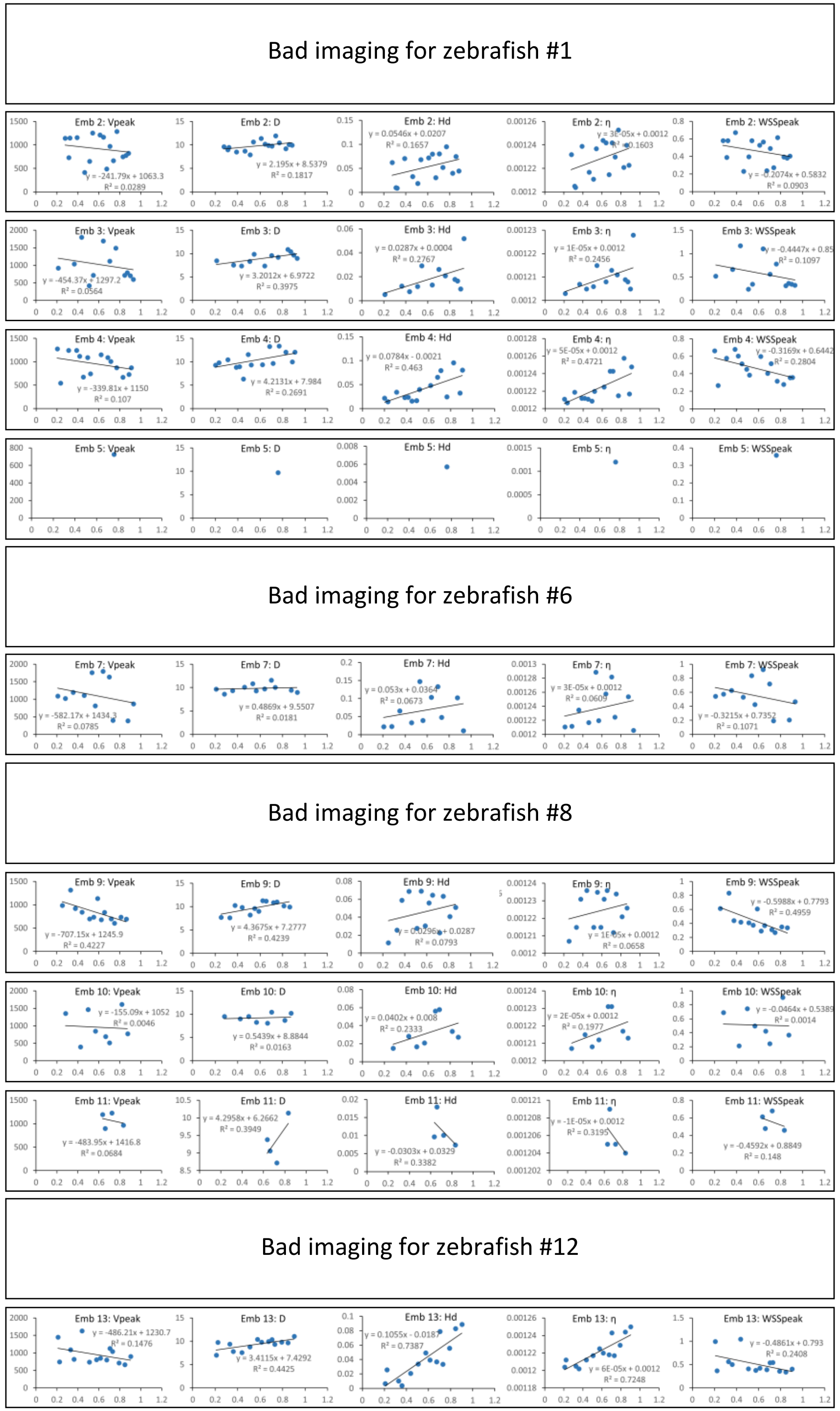

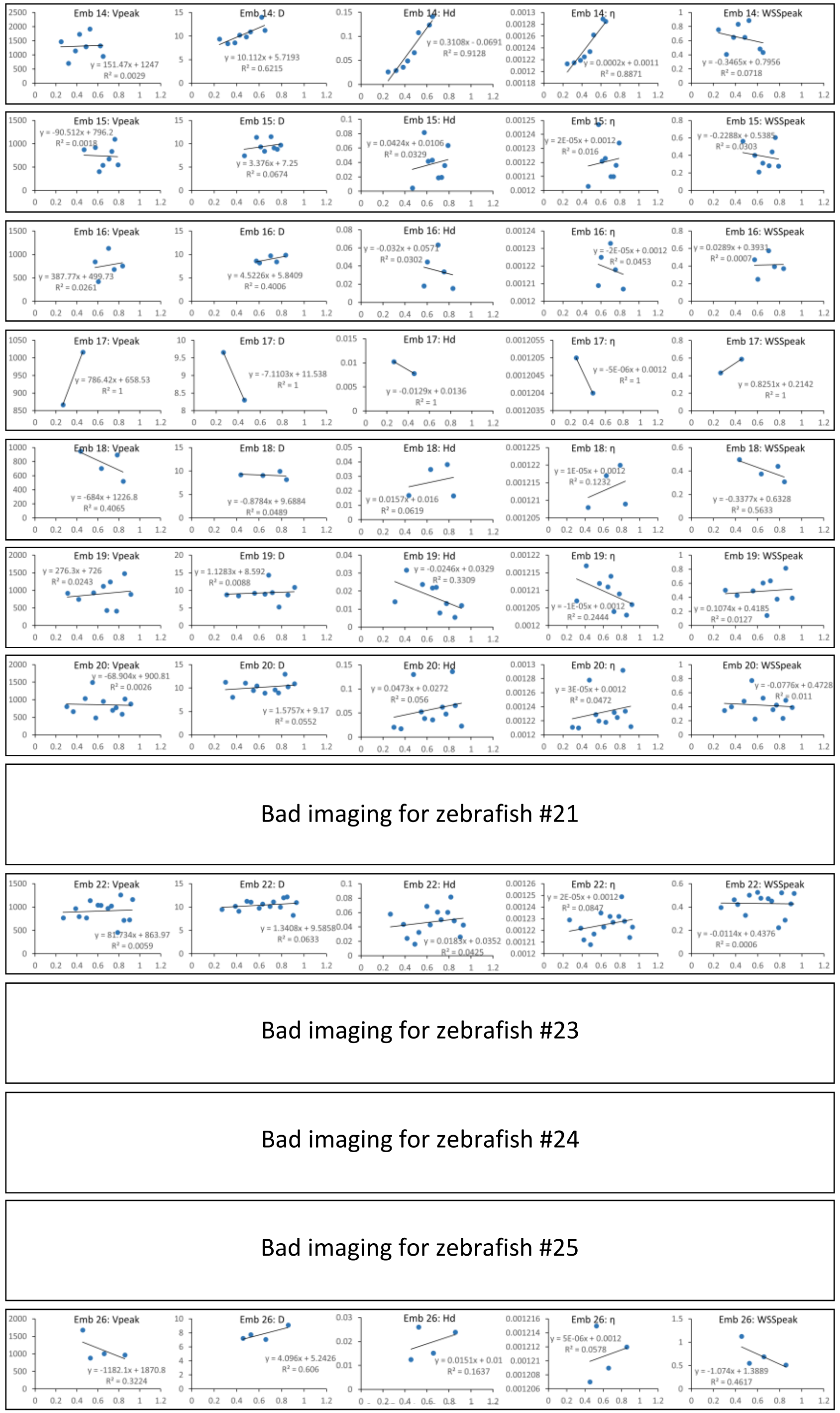

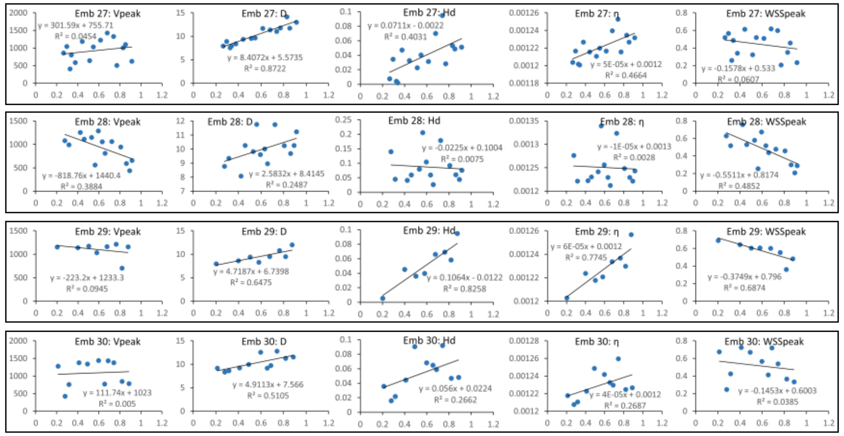

Figure S20. Arterial intersegmental vessel trends along the normalized anterior-posterior axis (x-axis of graphs) for systolic velocity Vpeak [μm/s], lumen diameter D [μm], discharge hematocrit fraction Hd [max is 1], apparent blood viscosity η [Pa·s] and systolic wall shear stress WSSpeak [Pa] in 2 dpf zebrafish.

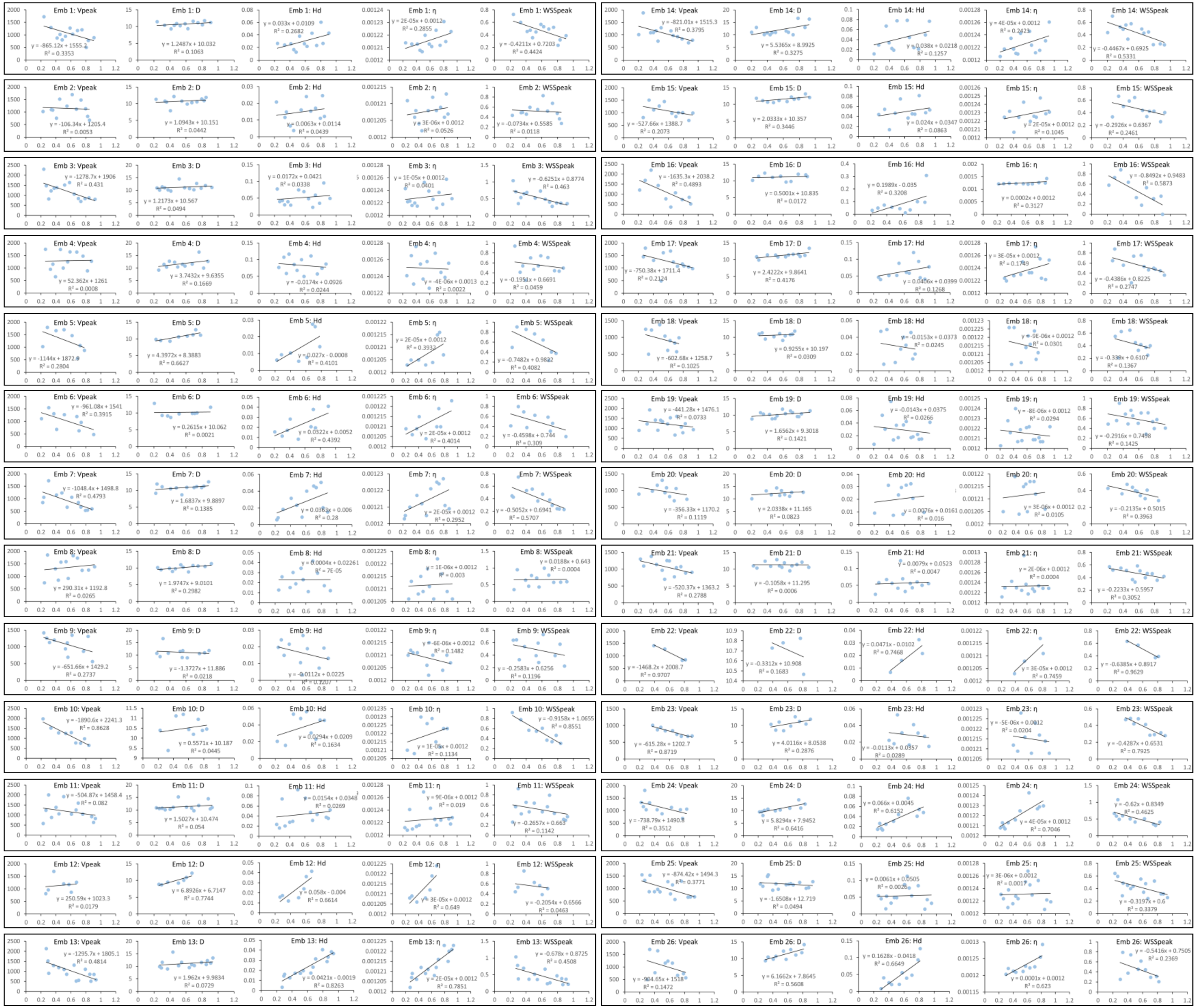

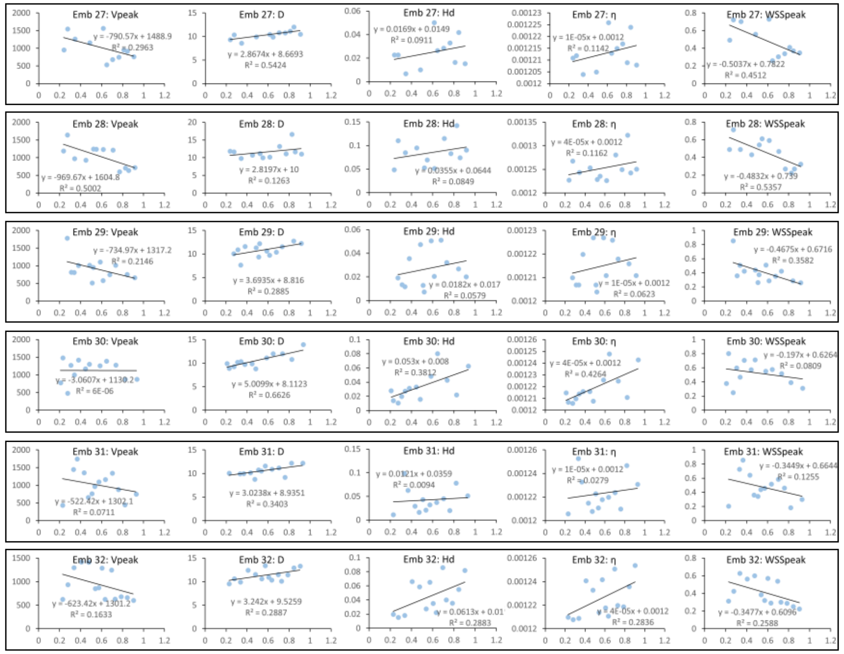

Figure S21. Arterial intersegmental vessel trends along the normalized anterior-posterior axis (x-axis of graphs) for systolic velocity Vpeak [μm/s], lumen diameter D [μm], discharge hematocrit fraction Hd [max is 1], apparent blood viscosity η [Pa·s] and systolic wall shear stress WSSpeak [Pa] in 3 dpf zebrafish.

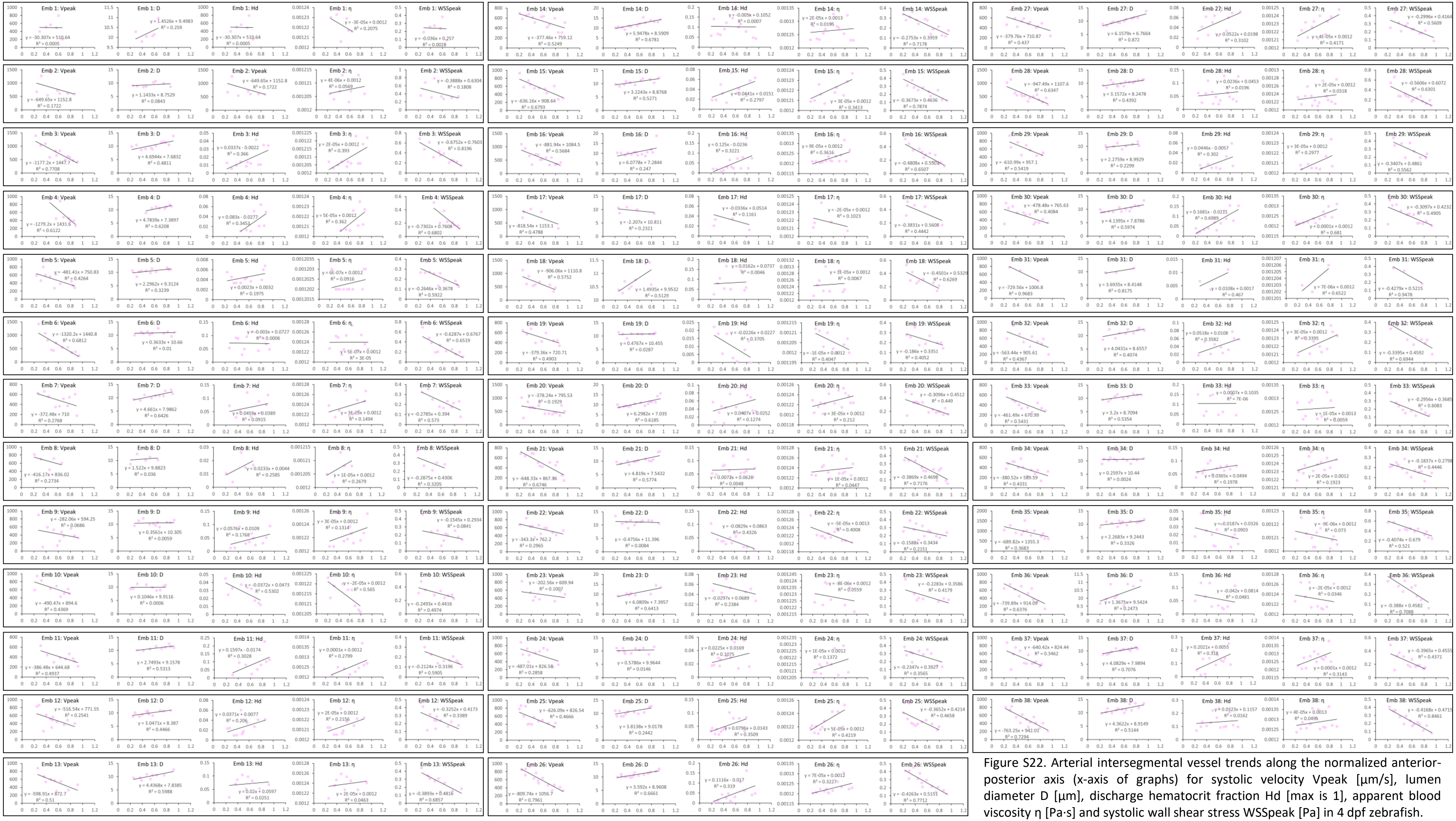

Figure S22. Arterial intersegmental vessel trends along the normalized anterior-posterior axis (x-axis of graphs) for systolic velocity Vpeak [µm/s], lumen diameter D [µm], discharge hematocrit fraction Hd [max is 1], apparent blood viscosity η [Pa·s] and systolic wall shear stress WSSpeak [Pa] in 4 dpf zebrafish.

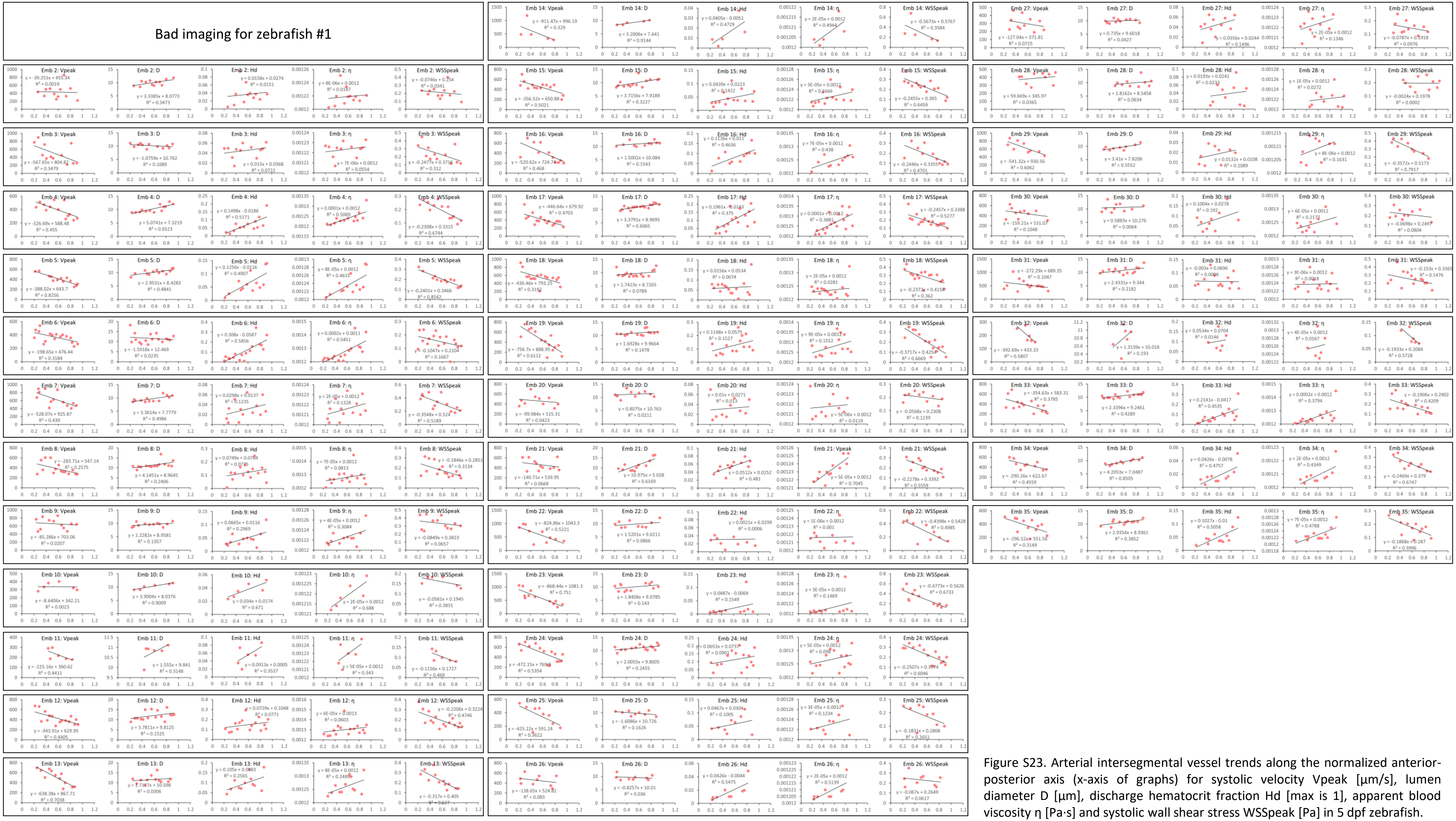

Figure S23. Arterial intersegmental vessel trends along the normalized anterior-posterior axis (x-axis of graphs) for systolic velocity Vpeak [μm/s], lumen diameter D [μm], discharge hematocrit fraction Hd [max is 1], apparent blood viscosity η [Pa·s] and systolic wall shear stress WSSpeak [Pa] in 5 dpf zebrafish.

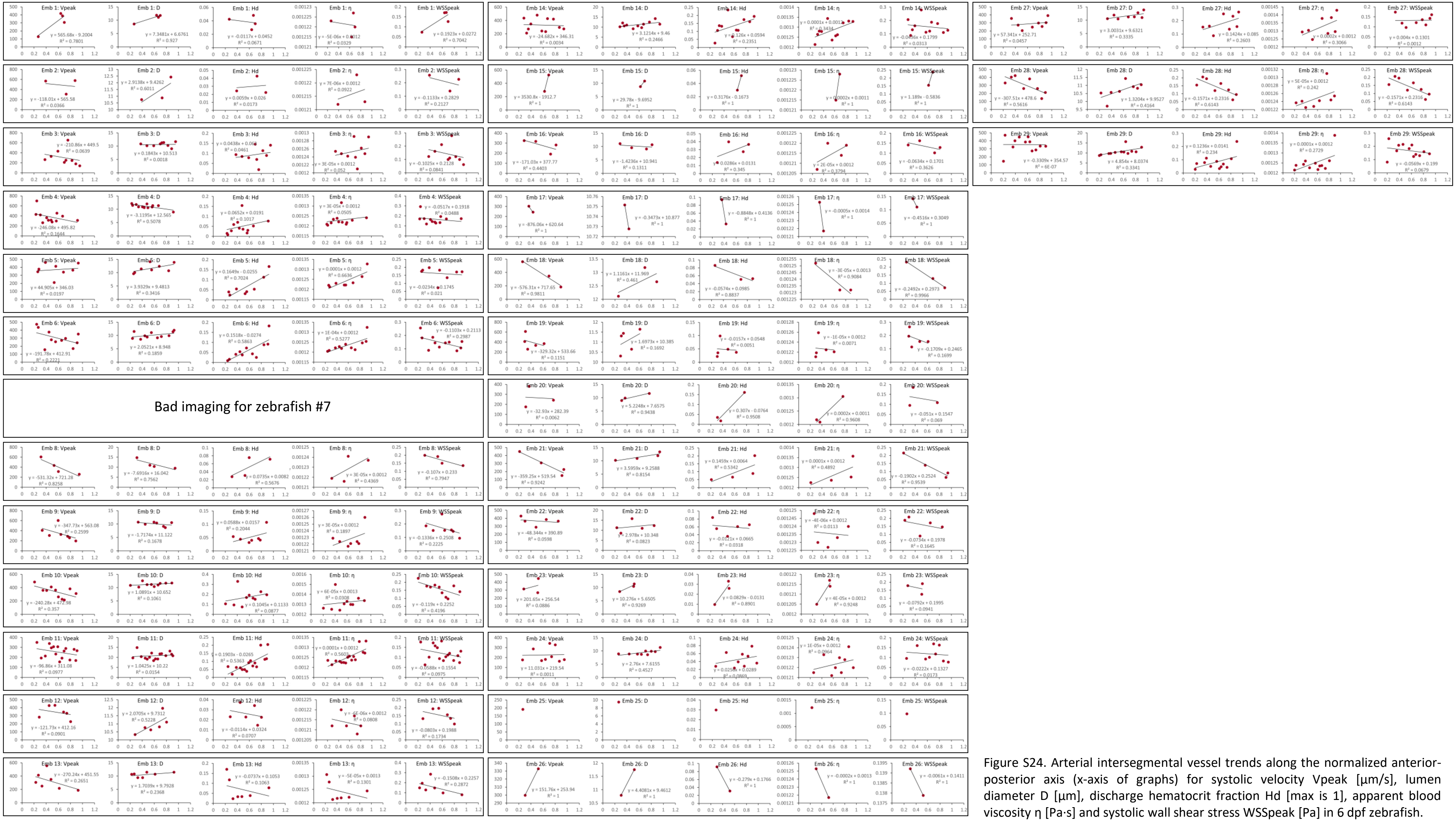

Figure S24. Arterial intersegmental vessel trends along the normalized anterior-posterior axis (x-axis of graphs) for systolic velocity Vpeak [μm/s], lumen diameter D [μm], discharge hematocrit fraction Hd [max is 1], apparent blood viscosity η [Pa·s] and systolic wall shear stress WSSpeak [Pa] in 6 dpf zebrafish.

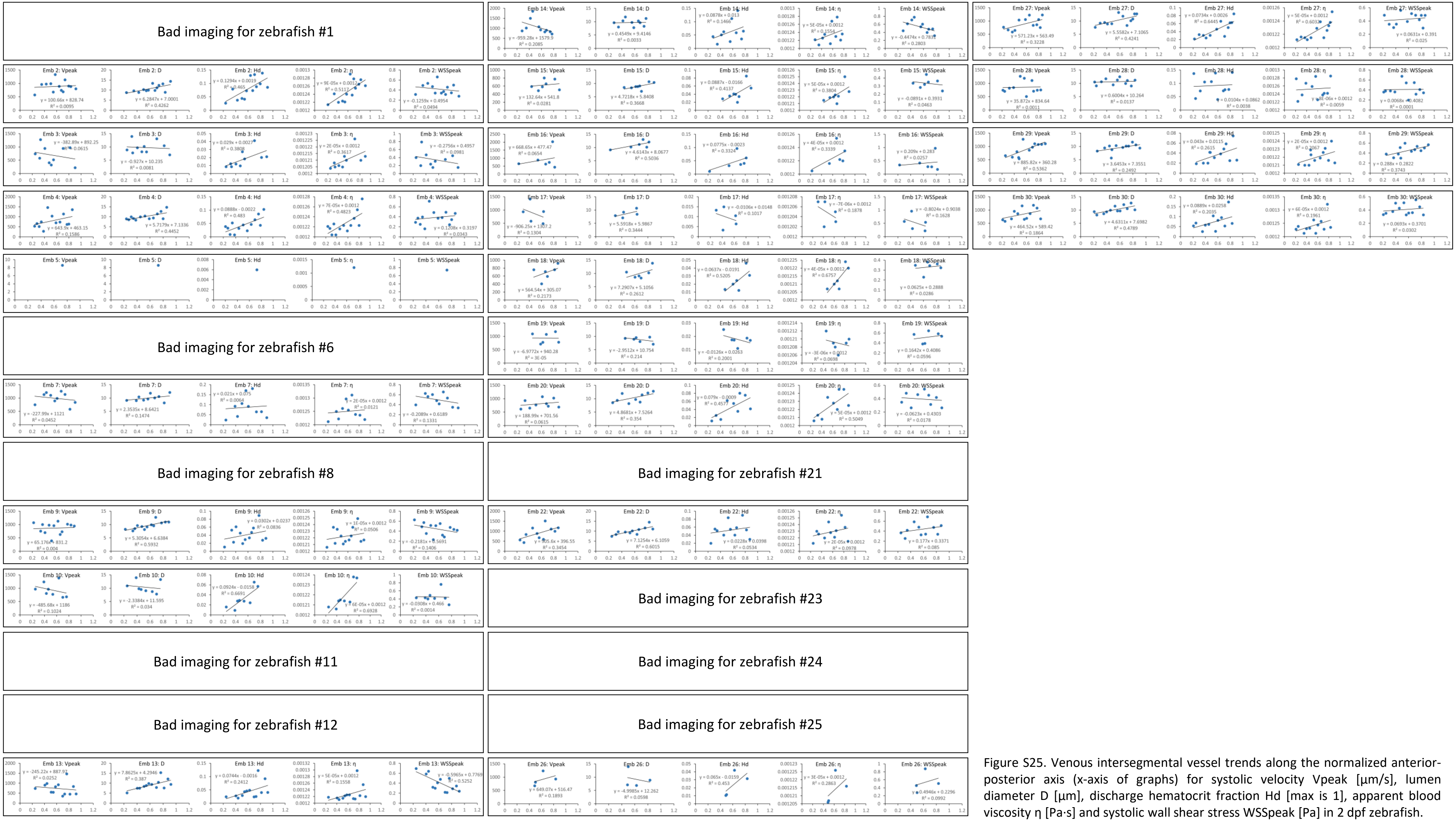

Figure S25. Venous intersegmental vessel trends along the normalized anterior-posterior axis (x-axis of graphs) for systolic velocity Vpeak [μm/s], lumen diameter D [μm], discharge hematocrit fraction Hd [max is 1], apparent blood viscosity η [Pa·s] and systolic wall shear stress WSSpeak [Pa] in 2 dpf zebrafish.

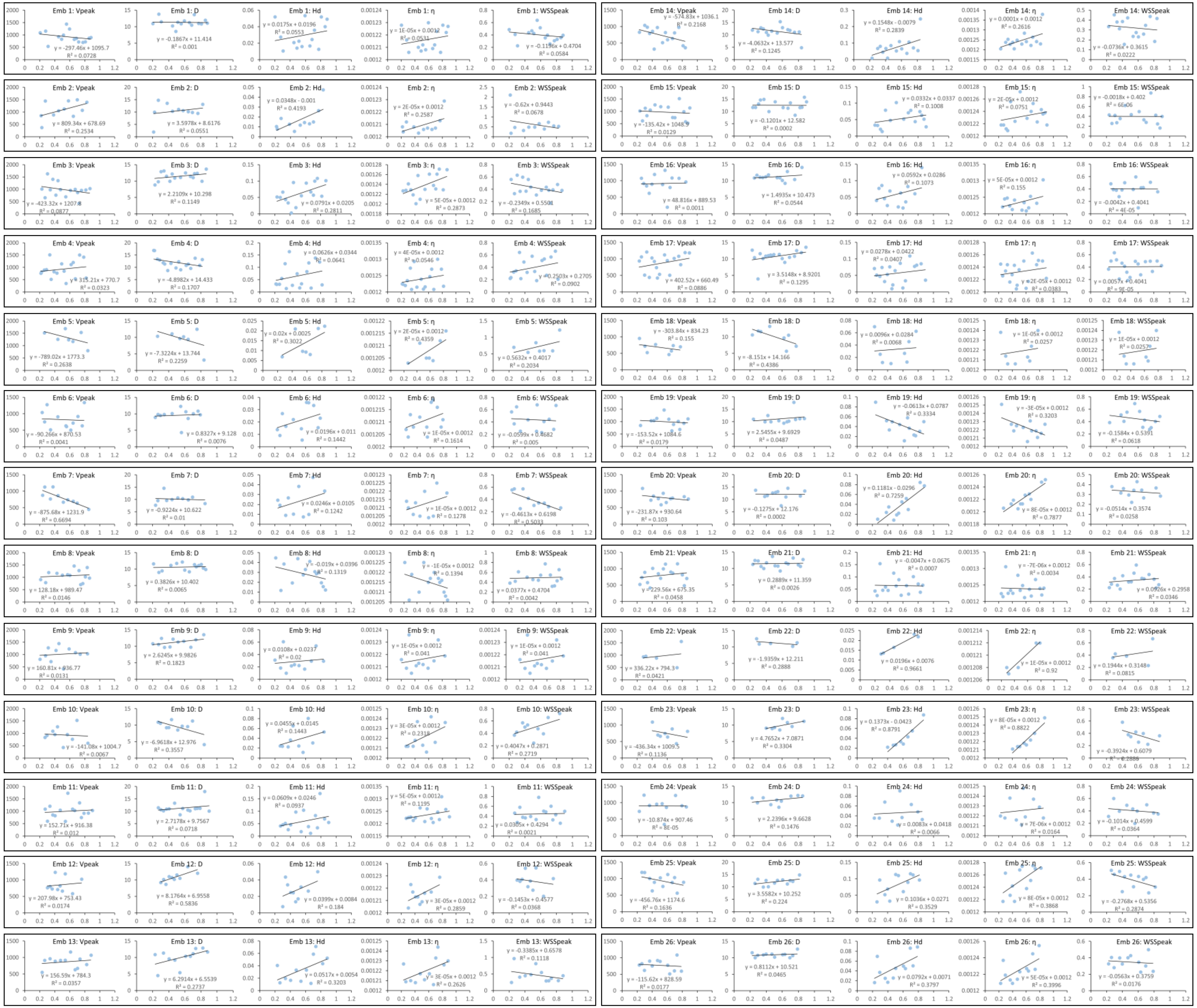

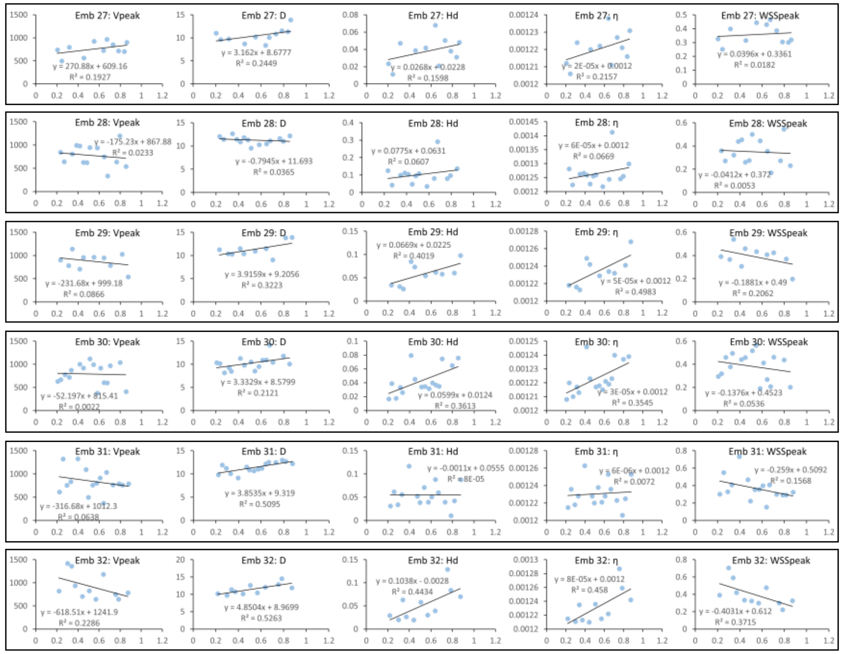

Figure S26. Venous intersegmental vessel trends along the normalized anterior-posterior axis (x-axis of graphs) for systolic velocity Vpeak [μm/s], lumen diameter D [μm], discharge hematocrit fraction Hd [max is 1], apparent blood viscosity η [Pa·s] and systolic wall shear stress WSSpeak [Pa] in 3 dpf zebrafish.

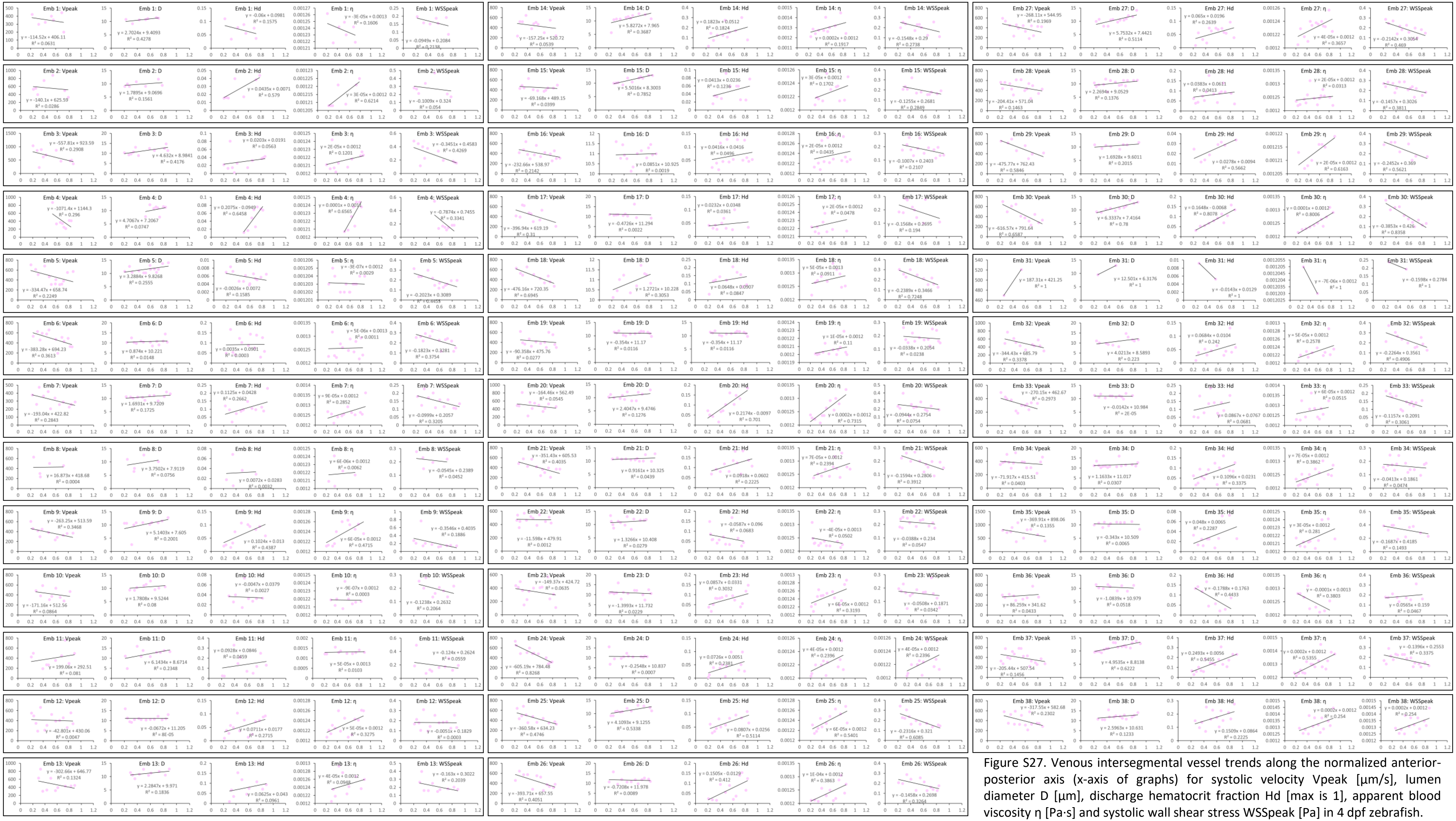

Figure S27. Venous intersegmental vessel trends along the normalized anterior-posterior axis (x-axis of graphs) for systolic velocity Vpeak [μm/s], lumen diameter D [μm], discharge hematocrit fraction Hd [max is 1], apparent blood viscosity η [Pa·s] and systolic wall shear stress WSSpeak [Pa] in 4 dpf zebrafish.

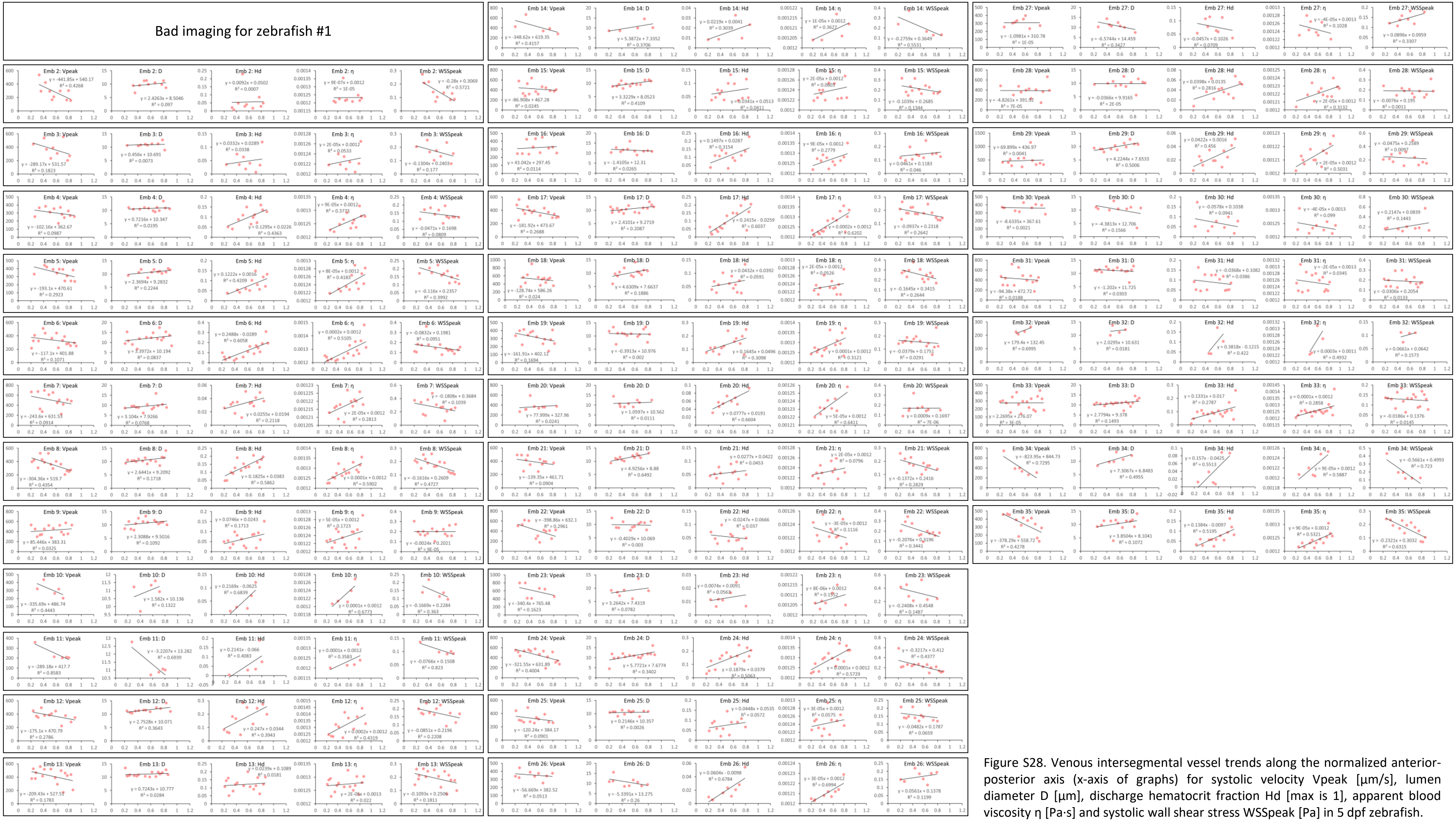

Figure S28. Venous intersegmental vessel trends along the normalized anterior-posterior axis (x-axis of graphs) for systolic velocity Vpeak [μm/s], lumen diameter D [μm], discharge hematocrit fraction Hd [max is 1], apparent blood viscosity η [Pa·s] and systolic wall shear stress WSSpeak [Pa] in 5 dpf zebrafish.

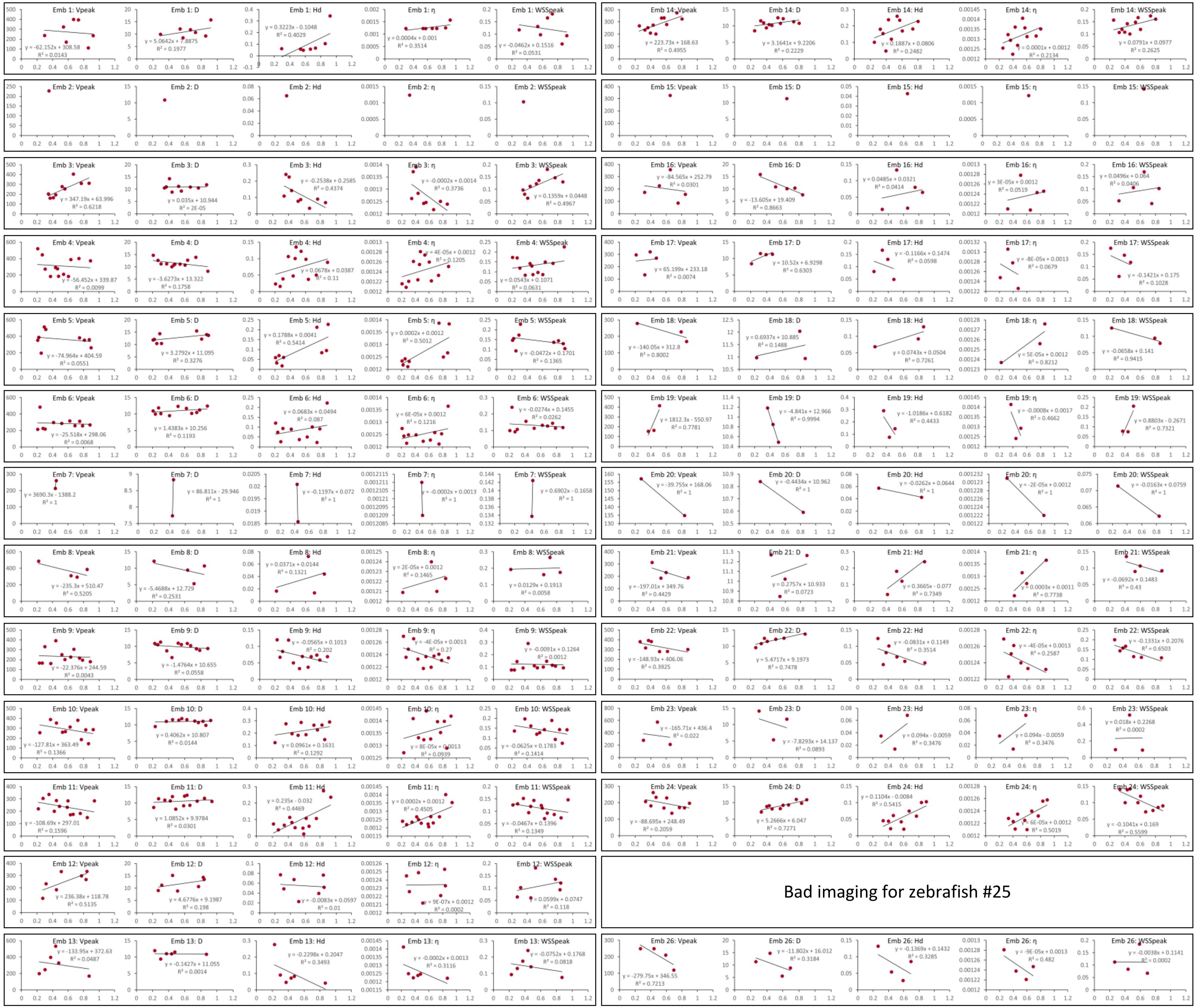

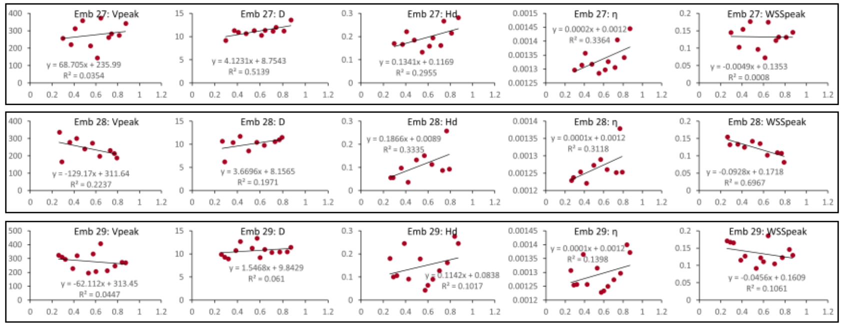

Figure S29. Venous intersegmental vessel trends along the normalized anterior-posterior axis (x-axis of graphs) for systolic velocity Vpeak [μm/s], lumen diameter D [μm], discharge hematocrit fraction Hd [max is 1], apparent blood viscosity η [Pa·s] and systolic wall shear stress WSSpeak [Pa] in 6 dpf zebrafish.

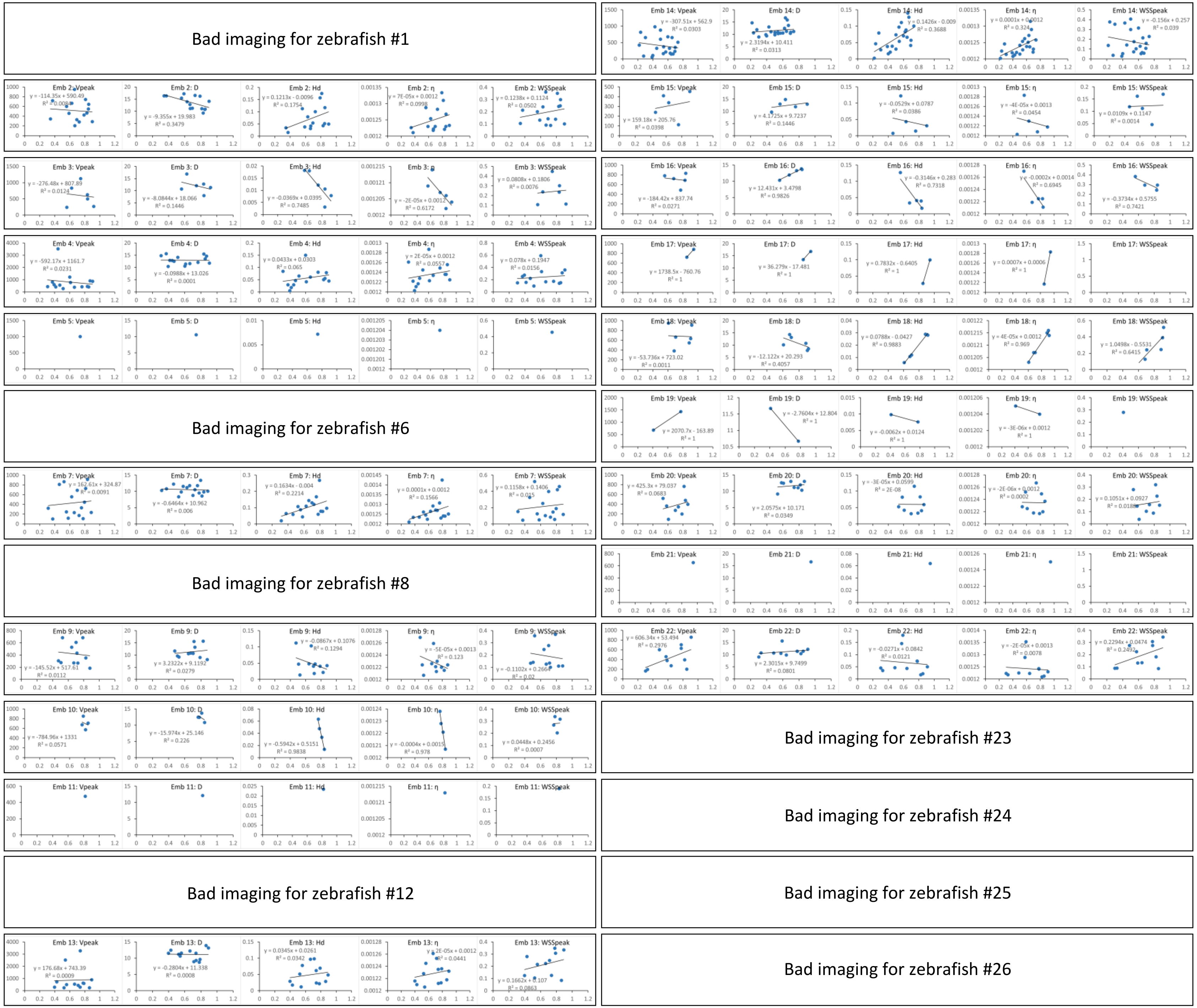

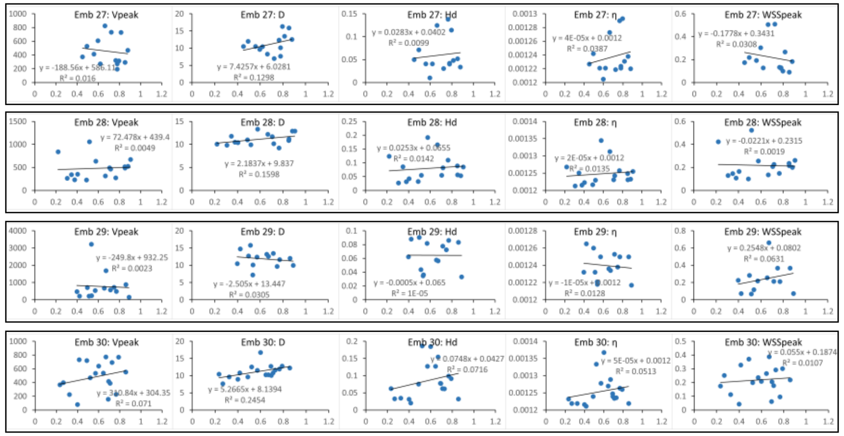

Figure S30. Dorsal longitudinal anastomotic vessel trends along the normalized anterior-posterior axis (x-axis of graphs) for systolic velocity Vpeak [μm/s], lumen diameter D [μm], discharge hematocrit fraction Hd [max is 1], apparent blood viscosity η [Pa·s] and systolic wall shear stress WSSpeak [Pa] in 2 dpf zebrafish.

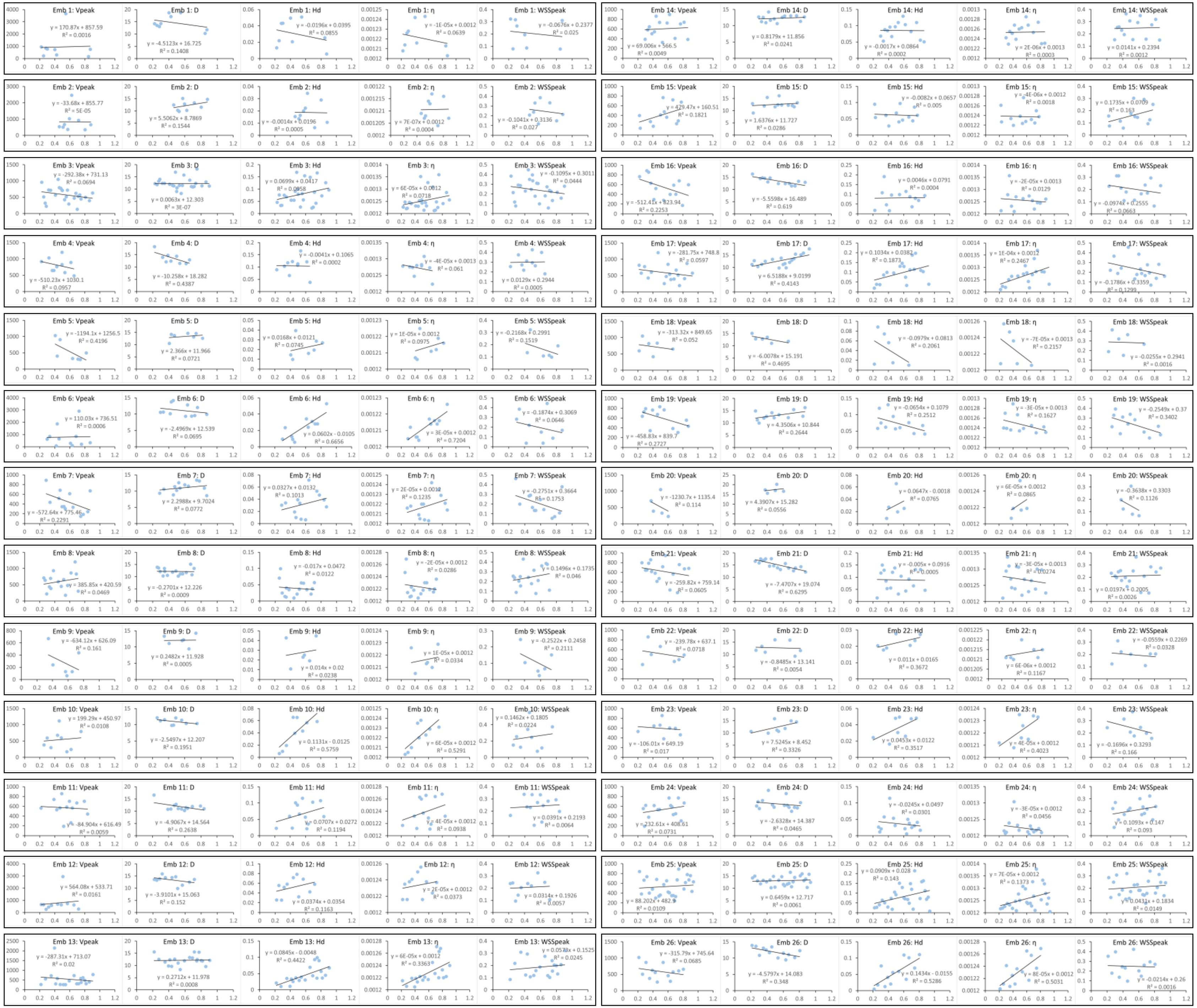

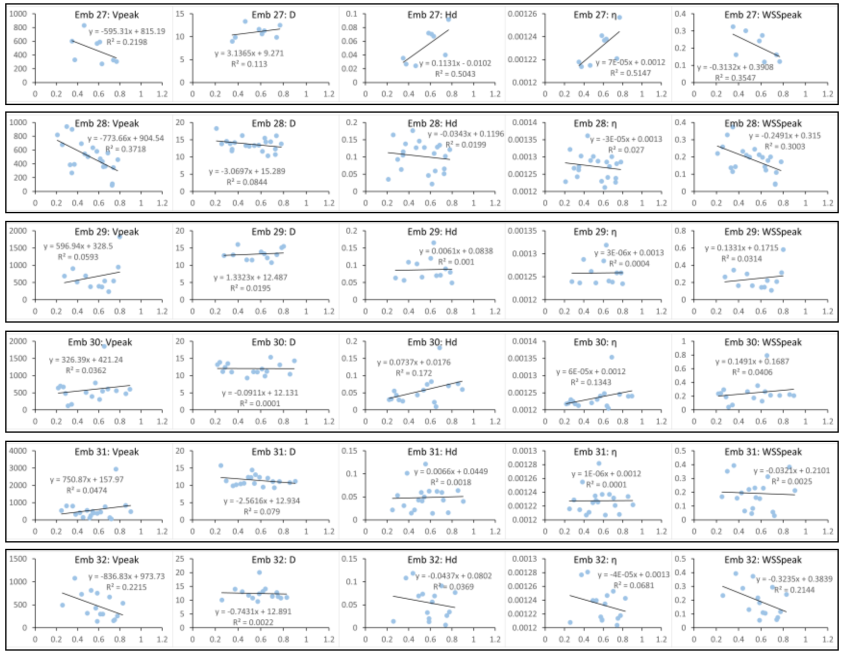

Figure S31. Dorsal longitudinal anastomotic vessel trends along the normalized anterior-posterior axis (x-axis of graphs) for systolic velocity Vpeak [µm/s], lumen diameter D [µm], discharge hematocrit fraction Hd [max is 1], apparent blood viscosity η [Pa·s] and systolic wall shear stress WSSpeak [Pa] in 3 dpf zebrafish.

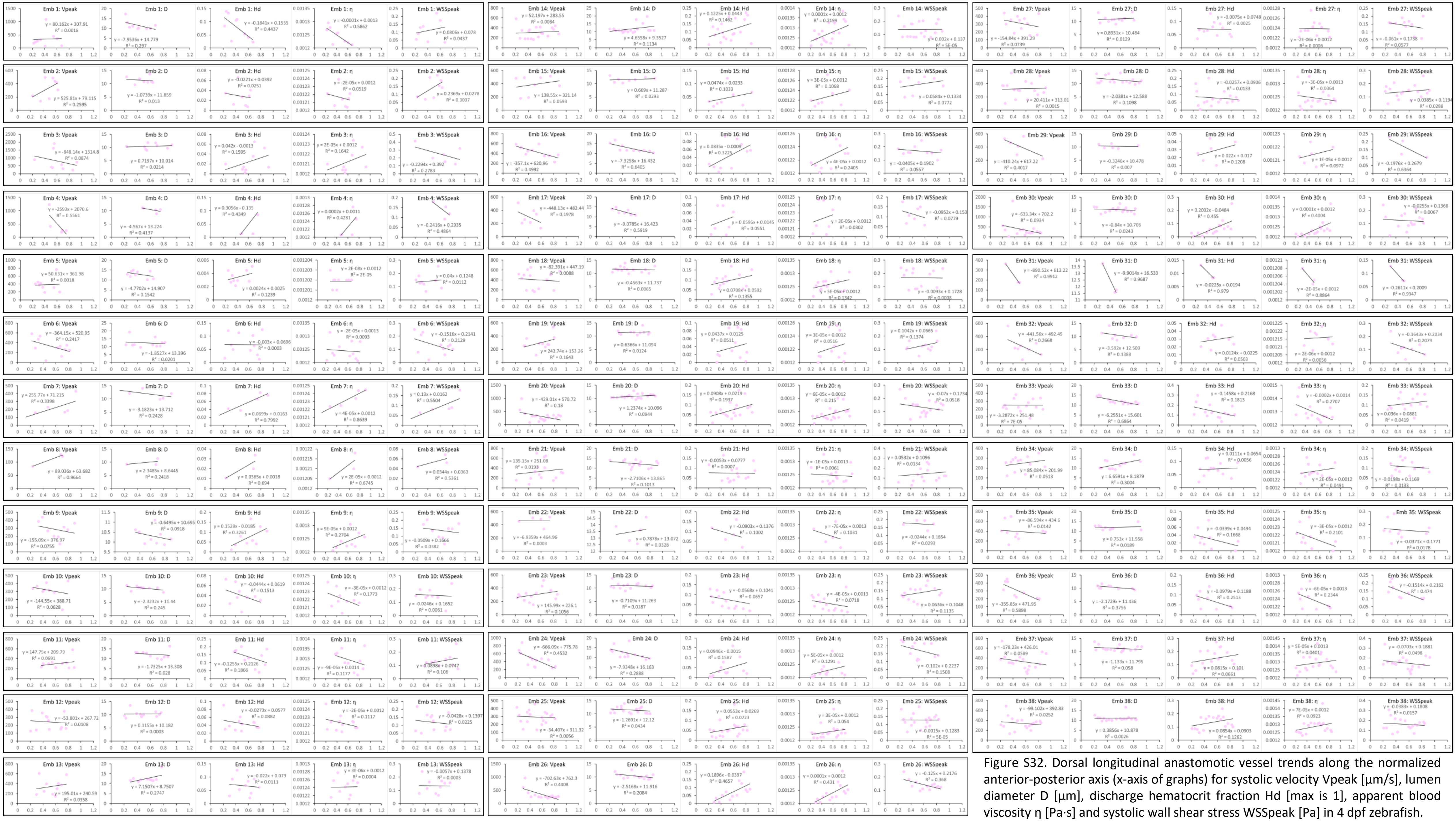

Figure S32. Dorsal longitudinal anastomotic vessel trends along the normalized anterior-posterior axis (x-axis of graphs) for systolic velocity Vpeak [μm/s], lumen diameter D [μm], discharge hematocrit fraction Hd [max is 1], apparent blood viscosity η [Pa·s] and systolic wall shear stress WSSpeak [Pa] in 4 dpf zebrafish.

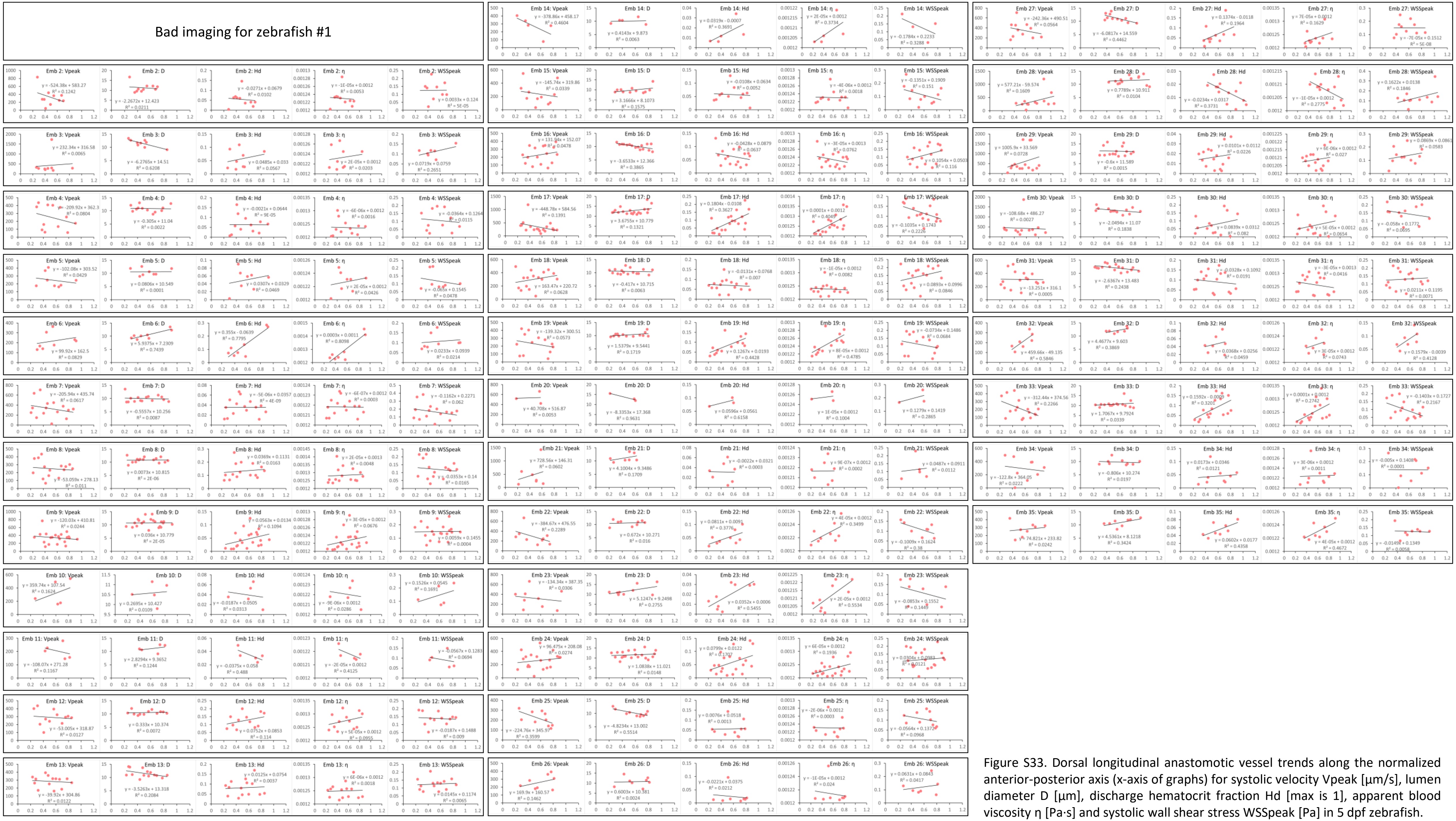

Figure S33. Dorsal longitudinal anastomotic vessel trends along the normalized anterior-posterior axis (x-axis of graphs) for systolic velocity Vpeak [μm/s], lumen diameter D [μm], discharge hematocrit fraction Hd [max is 1], apparent blood viscosity η [Pa·s] and systolic wall shear stress WSSpeak [Pa] in 5 dpf zebrafish.

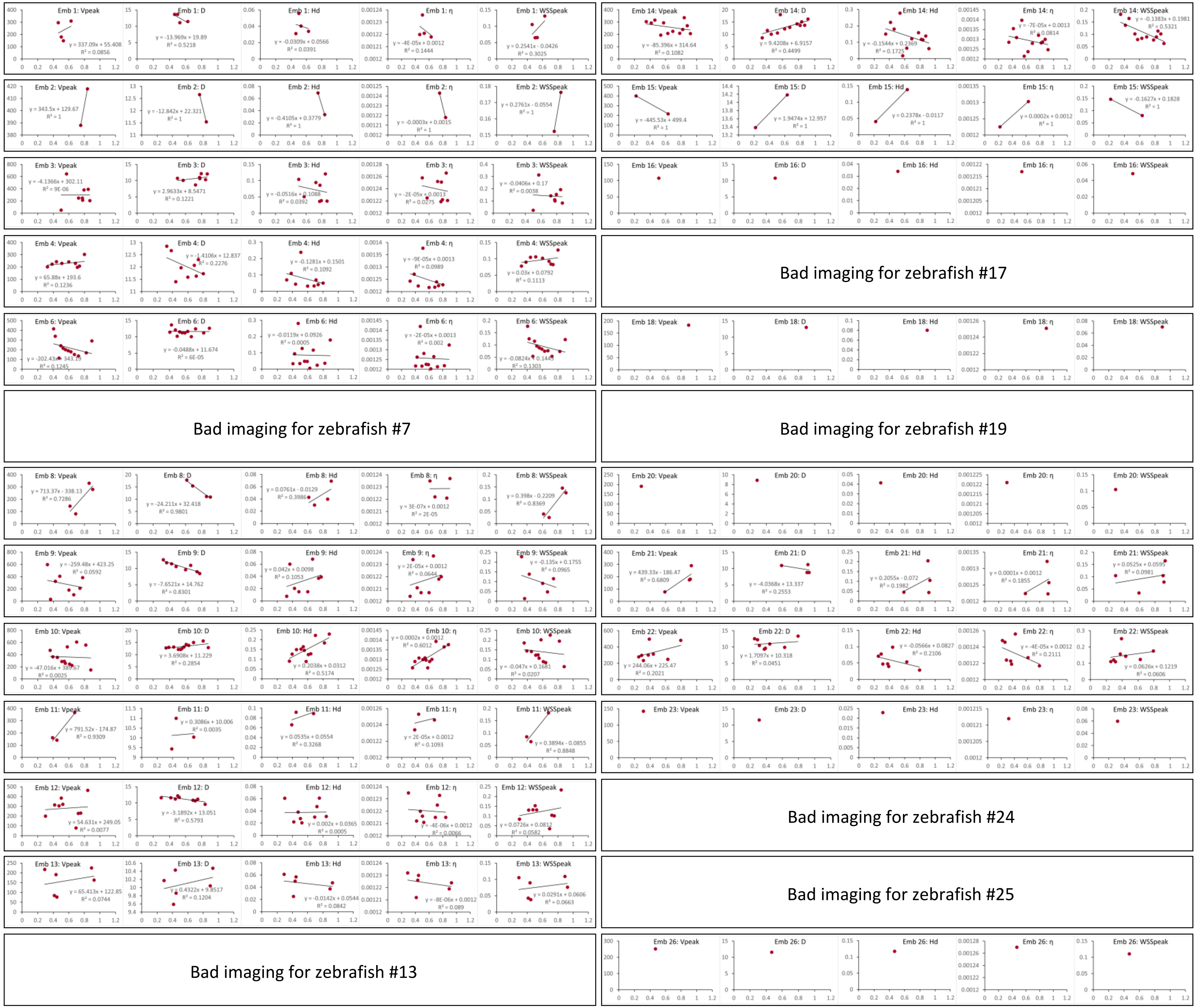

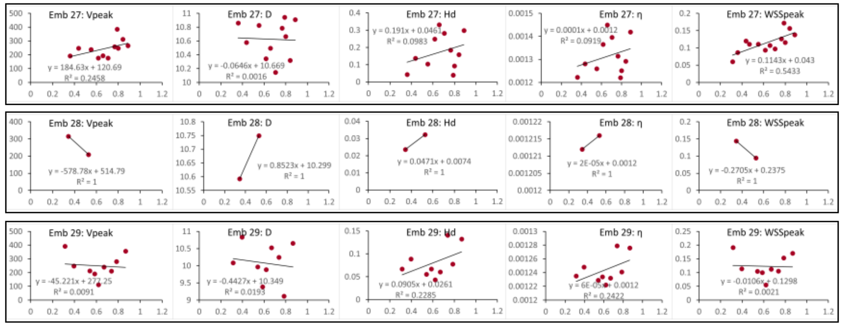

Figure S34. Dorsal longitudinal anastomotic vessel trends along the normalized anterior-posterior axis (x-axis of graphs) for systolic velocity Vpeak [μm/s], lumen diameter D [μm], discharge hematocrit fraction Hd [max is 1], apparent blood viscosity η [Pa·s] and systolic wall shear stress WSSpeak [Pa] in 6 dpf zebrafish.

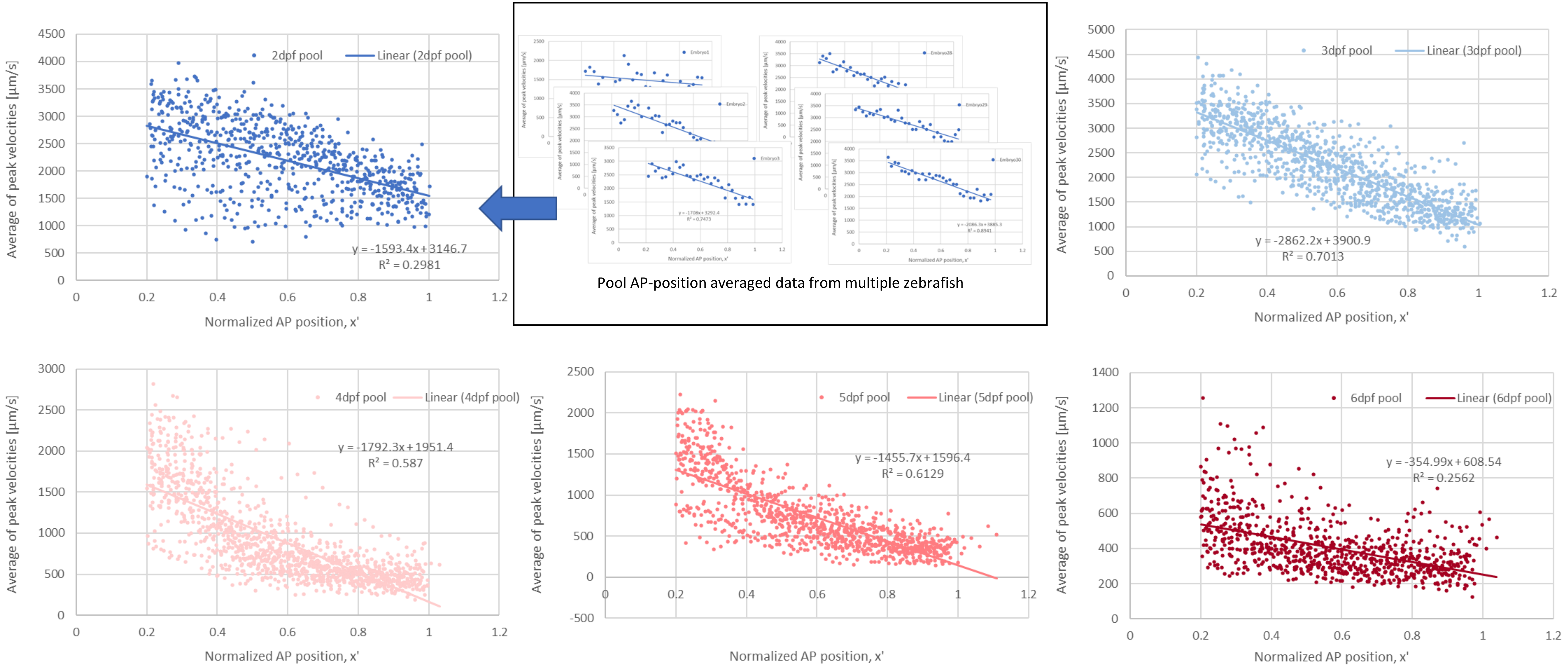

Figure S35. Linear regression fit to describe the anterior-posterior (AP) trend for analyzed AP-position averaged quantities in the DA/CA (see Fig. 2C of main article for definition of AP-position averaging) after data pooling from multiple zebrafish at same developmental day [dpf]. Trends for individual fish were shown in figures S10 – S34.

Supplementary table 1. Regression statistics for pooled DA/CA data

DA/CA		Peak velocity table									
dpf	n	Regression model for data from $\hat{x}$ > 0.2	R^2	S.E.R	p value	p<0.05?	val@$\hat{x}$ =0	gradient	val@$\hat{x}$=0.2	val@$\hat{x}$=1	%change btwn $\hat{x}$=0.2 and $\hat{x}$=1
2	704	vpeak = -1593.4 $\hat{x}$ + 3146.7	0.298109	543.9144	6.04E-56	YES	3146.7	-1593.4	2828.02	1553.3	-45.07
3	1026	vpeak = -2862.2$\hat{x}$ + 3900.9	0.701273	416.944	6.52E-271	YES	3900.9	-2862.2	3328.46	1038.7	-68.79
4	1213	vpeak = -1792.5$\hat{x}$ + 1951.6	0.586494	336.3054	1.80E-234	YES	1951.6	-1792.5	1593.1	159.1	-90.01
5	991	vpeak = -1455.7$\hat{x}$ + 1596.4	0.612908	265.2964	4.84E-206	YES	1596.4	-1455.7	1305.26	140.7	-89.22
6	802	vpeak = -354.99$\hat{x}$ + 608.54	0.256232	136.2516	2.09E-53	YES	608.54	-354.99	537.542	253.55	-52.83
		Lumen diameter table									
dpf	n	Regression model for data from $\hat{x}$ > 0.2	R^2	S.E.R	p value	p<0.05?	val@$\hat{x}$ =0	gradient	val@$\hat{x}$=0.2	val@$\hat{x}$=1	%change btwn $\hat{x}$=0.2 and $\hat{x}$=1
2	704	D = -1.3551$\hat{x}$ + 21.843	0.006095	3.849656	3.84E-02	YES	21.843	-1.3551	21.57198	20.4879	-5.03
3	1026	D = -2.216$\hat{x}$ + 19.861	0.033991	2.636741	2.69E-09	YES	19.861	-2.216	19.4178	17.645	-9.13
4	1213	D = -0.9859$\hat{x}$ + 13.868	0.010953	2.093331	2.61E-04	YES	13.868	-0.9859	13.67082	12.8821	-5.77
5	991	D = -1.8227$\hat{x}$ + 14.471	0.031952	2.300775	1.46E-08	YES	14.471	-1.8227	14.10646	12.6483	-10.34
6	802	D = -0.1544$\hat{x}$ + 13.518	0.000331	1.913473	6.07E-01	NO	13.518	-0.1544	13.48712	13.3636	-0.92
		Discharge hematocrit table									
dpf	n	Regression model for data from $\hat{x}$ > 0.2	R^2	S.E.R	p value	p<0.05?	val@$\hat{x}$ =0	gradient	val@$\hat{x}$=0.2	val@$\hat{x}$=1	%change btwn $\hat{x}$=0.2 and $\hat{x}$=1
2	704	Hd = -0.0034$\hat{x}$ + 0.1547	0.000104	0.073262	7.88E-01	NO	0.1547	-0.0034	0.15402	0.1513	-1.77
3	1026	Hd = 0.0651$\hat{x}$ + 0.1249	0.066263	0.054565	5.44E-17	YES	0.1249	0.0651	0.13792	0.19	37.76
4	1213	Hd = 0.0634$\hat{x}$ + 0.1332	0.027867	0.083718	4.94E-09	YES	0.1332	0.0634	0.14588	0.1966	34.77
5	991	Hd = 0.1174$\hat{x}$ + 0.1226	0.083936	0.088914	1.29E-20	YES	0.1226	0.1174	0.14608	0.24	64.29
6	802	Hd = 0.0896$\hat{x}$ + 0.1715	0.055419	0.083342	1.46E-11	YES	0.1715	0.0896	0.18942	0.2611	37.84
		Viscosity table									
dpf	n	Regression model for data from $\hat{x}$ > 0.2	R^2	S.E.R	p value	p<0.05?	val@$\hat{x}$ =0	gradient	val@$\hat{x}$=0.2	val@$\hat{x}$=1	%change btwn $\hat{x}$=0.2 and $\hat{x}$=1
2	704	η = -1.58E-05$\hat{x}$ + 1.38E-03	0.001134	0.000104	3.72E-01	NO	1.38E-03	-1.58E-05	0.00137684	0.0013642	-0.92
3	1026	η = 6.07E-05$\hat{x}$ + 1.33E-03	0.038898	6.73E-05	1.86E-10	YES	1.33E-03	6.07E-05	0.00134214	0.0013907	3.62
4	1213	η = 5.38E-05$\hat{x}$ + 1.30E-03	0.019747	8.47E-05	8.95E-07	YES	1.30E-03	5.38E-05	0.00131076	0.0013538	3.28
5	991	η = 1.12E-04$\hat{x}$ + 1.29E-03	0.062784	9.91E-05	1.19E-15	YES	1.29E-03	1.12E-04	0.0013124	0.001402	6.83
6	802	η = 9.19E-05$\hat{x}$ + 1.34E-03	0.044805	9.56E-05	1.41E-09	YES	1.34E-03	9.19E-05	0.00135838	0.0014319	5.41
		Peak WSS table									
dpf	n	Regression model for data from $\hat{x}$ > 0.2	R^2	S.E.R	p value	p<0.05?	val@$\hat{x}$ =0	gradient	val@$\hat{x}$=0.2	val@$\hat{x}$=1	%change btwn $\hat{x}$=0.2 and $\hat{x}$=1
2	704	WSSpeak = -0.3808$\hat{x}$ + 0.8122	0.210217	0.164215	6.92E-38	YES	0.8122	-0.3808	0.73604	0.4314	-41.39
3	1026	WSSpeak = -0.7658$\hat{x}$ + 1.1045	0.570762	0.148236	2.88E-190	YES	1.1045	-0.7658	0.95134	0.3387	-64.40
4	1213	WSSpeak = -0.6817$\hat{x}$ + 0.7604	0.57528	0.130872	1.98E-227	YES	0.7604	-0.6817	0.62406	0.0787	-87.39
5	991	WSSpeak = -0.5223$\hat{x}$ + 0.6062	0.53742	0.111128	9.41E-168	YES	0.6062	-0.5223	0.50174	0.0839	-83.28
6	802	WSSpeak = -0.1378$\hat{x}$ + 0.2468	0.229571	0.056879	2.89E-47	YES	0.2468	-0.1378	0.21924	0.109	-50.28
		Peak PSR table									
dpf	n	Regression model for data from $\hat{x}$ > 0.2	R^2	S.E.R	p value	p<0.05?	val@$\hat{x}$ =0	gradient	val@$\hat{x}$=0.2	val@$\hat{x}$=1	%change btwn $\hat{x}$=0.2 and $\hat{x}$=1
2	704	PSR = -33.646$\hat{x}$ +73.053	0.229095	13.73092	1.36E-41	YES	73.053	-33.646	66.3238	39.407	-40.58
3	1026	PSR = -73.148$\hat{x}$ + 103.25	0.593083	13.52351	3.8E-202	YES	103.25	-73.148	88.6204	30.102	-66.03
4	1213	PSR = -65.482$\hat{x}$ + 72.546	0.565048	12.83679	3.6E-221	YES	72.546	-65.482	59.4496	7.064	-88.12
5	991	PSR = -50.269$\hat{x}$ + 57.442	0.532425	10.80304	1.9E-165	YES	57.422	-50.269	47.3682	7.153	-84.90
6	802	PSR = -13.587$\hat{x}$ + 23.085	0.230765	5.588611	1.55E-47	YES	23.085	-13.587	20.3676	9.498	-53.37

Supplementary table 2. Regression statistics for pooled PCV/CV data

PCV/CV		Peak velocity table									
dpf	n	Regression model for data from $\hat{x}$ > 0.2	R^2	S.E.R	p value	p<0.05?	val@$\hat{x}$ =0	gradient	val@$\hat{x}$=0.2	val@$\hat{x}$=1	%change btwn $\hat{x}$=0.2 and $\hat{x}$=1
2	723	vpeak = -463.42$\hat{x}$ + 1593.2	0.088936	313.6569	2.57E-16	YES	1593.2	-462.42	1500.716	1130.78	-24.65
3	927	vpeak = -750.68$\hat{x}$ + 1581	0.281303	259.9359	2.22E-68	YES	1581	-750.68	1430.864	830.32	-41.97
4	886	vpeak = -423.74$\hat{x}$ + 762.8	0.210758	167.7048	2.15E-47	YES	762.8	-423.74	678.052	339.06	-49.99
5	920	vpeak = -430.13$\hat{x}$ + 678.74	0.307882	139.9299	2.08E-75	YES	678.74	-430.13	592.714	248.61	-58.06
6	647	vpeak = -85.081$\hat{x}$ + 366.82	0.027078	112.0534	2.60E-05	YES	366.82	-85.081	349.8038	281.739	-19.46
		Lumen diameter table									
dpf	n	Regression model for data from $\hat{x}$ > 0.2	R^2	S.E.R	p value	p<0.05?	val@$\hat{x}$ =0	gradient	val@$\hat{x}$=0.2	val@$\hat{x}$=1	%change btwn $\hat{x}$=0.2 and $\hat{x}$=1
2	723	D = -14.906$\hat{x}$ + 29.971	0.284685	4.996406	1.95E-54	YES	29.971	-14.906	26.9898	15.065	-44.18
3	927	D = -16.084$\hat{x}$ + 31.304	0.517185	3.366657	2.04E-148	YES	31.304	-16.084	28.0872	15.22	-45.81
4	886	D = -10.716$\hat{x}$ + 23.841	0.403564	2.664429	2.65E-101	YES	23.841	-10.716	21.6978	13.125	-39.51
5	920	D = -11.435$\hat{x}$ + 24.674	0.388789	3.110902	3.07E-100	YES	24.674	-11.435	22.387	13.239	-40.86
6	647	D = -7.5428$\hat{x}$ + 21.634	0.235937	2.982386	1.31E-39	YES	21.634	-7.5428	20.12544	14.0912	-29.98
		Discharge hematocrit table									
dpf	n	Regression model for data from $\hat{x}$ > 0.2	R^2	S.E.R	p value	p<0.05?	val@$\hat{x}$ =0	gradient	val@$\hat{x}$=0.2	val@$\hat{x}$=1	%change btwn $\hat{x}$=0.2 and $\hat{x}$=1
2	723	Hd = -0.0262$\hat{x}$ + 0.1775	0.004909	0.078918	5.97E-02	NO	0.1775	-0.0262	0.17226	0.1513	-12.17
3	927	Hd = 0.0164$\hat{x}$ + 0.1871	0.003818	0.057228	6.00E-02	NO	0.1871	0.0164	0.19038	0.2035	6.89
4	886	Hd = 0.0215$\hat{x}$ + 0.2011	0.002381	0.0899	1.47E-01	NO	0.2011	0.0215	0.2054	0.2226	8.37
5	920	Hd = 0.0371$\hat{x}$ + 0.2211	0.008286	0.088165	5.73E-03	YES	0.2211	0.0371	0.22852	0.2582	12.99
6	647	Hd = 0.0143$\hat{x}$ + 0.2633	0.001391	0.084083	3.43E-01	NO	0.2633	0.0143	0.26616	0.2776	4.30
		Viscosity table									
dpf	n	Regression model for data from $\hat{x}$ > 0.2	R^2	S.E.R	p value	p<0.05?	val@$\hat{x}$ =0	gradient	val@$\hat{x}$=0.2	val@$\hat{x}$=1	%change btwn $\hat{x}$=0.2 and $\hat{x}$=1
2	723	η = -1.51E-04$\hat{x}$ + 1.48E-03	0.064738	0.000121	3.80E-12	YES	1.48E-03	-1.51E-05	0.00147698	0.0014649	-0.82
3	927	η = -1.10E-04$\hat{x}$ + 1.49E-03	0.072456	8.51E-05	7.50E-17	YES	1.49E-03	1.10E-04	0.001512	0.0016	5.82
4	886	η = -7.95E-05$\hat{x}$ + 1.47E-03	0.016322	0.000126	1.37E-04	YES	1.47E-03	7.95E-05	0.0014859	0.0015495	4.28
5	920	η = -7.37E-05$\hat{x}$ + 1.51E-03	0.014458	0.000132	2.57E-04	YES	1.51E-03	7.37E-05	0.00152474	0.0015837	3.87
6	647	η = -8.60E-05$\hat{x}$ + 1.55E-03	0.018613	0.000137	5.02E-04	YES	1.55E-03	8.60E-05	0.0015672	0.001636	4.39
		Peak WSS table									
dpf	n	Regression model for data from $\hat{x}$ > 0.2	R^2	S.E.R	p value	p<0.05?	val@$\hat{x}$ =0	gradient	val@$\hat{x}$=0.2	val@$\hat{x}$=1	%change btwn $\hat{x}$=0.2 and $\hat{x}$=1
2	723	WSSpeak = 0.1325$\hat{x}$ + 0.2862	0.056433	0.114584	9.84E-11	YES	0.2862	0.1325	0.3127	0.4187	33.90
3	927	WSSpeak = 0.0056$\hat{x}$ + 0.2994	0.00026	0.074599	0.623717	NO	0.2994	0.0056	0.30052	0.305	1.49
4	886	WSSpeak = -0.0422$\hat{x}$ + 0.1911	0.02922	0.049705	3.08E-07	YES	0.1911	-0.0422	0.18266	0.1489	-18.48
5	920	WSSpeak = -0.059$\hat{x}$ + 0.1739	0.071475	0.046121	1.60E-16	YES	0.1739	-0.059	0.1621	0.1149	-29.12
6	647	WSSpeak = 0.011$\hat{x}$ + 0.1046	0.003848	0.0388	0.114961	NO	0.1046	0.011	0.1068	0.1156	8.24
		Peak PSR table									
dpf	n	Regression model for data from $\hat{x}$ > 0.2	R^2	S.E.R	p value	p<0.05?	val@$\hat{x}$ =0	gradient	val@$\hat{x}$=0.2	val@$\hat{x}$=1	%change btwn $\hat{x}$=0.2 and $\hat{x}$=1
2	704	PSR = 15.742$\hat{x}$ + 23.703	0.09175	10.4734	8.29E-17	YES	23.703	15.742	26.8514	39.445	46.90
3	1026	PSR = 2.5915$\hat{x}$ + 25.067	0.006563	6.907238	0.013614	YES	25.067	2.5915	25.5853	27.6585	8.10
4	1213	PSR = -3.1086$\hat{x}$ + 16.546	0.018214	4.667676	5.58E-05	YES	16.546	-3.1086	15.92428	13.4374	-15.62
5	991	PSR = -4.3764$\hat{x}$ + 14.519	0.051852	4.060505	2.76E-12	YES	14.519	-4.3764	13.64372	10.1426	-25.66
6	802	PSR = 1.3422$\hat{x}$ + 8.5814	0.007042	3.501919	0.032833	YES	8.5814	1.3422	8.84984	9.9236	12.13

Supplementary table 3. Regression statistics for pooled aISV data

aISVs		Peak velocity table									
dpf	n	Regression model for data from $\hat{x}$ > 0.2	R^2	S.E.R	p value	p<0.05?	val@$\hat{x}$ =0	gradient	val@$\hat{x}$=0.2	val@$\hat{x}$=1	%change btwn $\hat{x}$=0.2 and $\hat{x}$=1
2	208	vpeak = -326.16$\hat{x}$ + 1152.1	0.042866	313.643	2.69E-03	YES	1152.1	-326.16	1086.868	825.94	-24.01
3	366	vpeak = -717.75$\hat{x}$ + 1486.3	0.179367	321.8935	2.31E-17	YES	1486.3	-717.75	1342.75	768.55	-42.76
4	404	vpeak = -582.21$\hat{x}$ + 884.78	0.304842	186.3136	1.30E-33	YES	884.78	-582.21	768.338	302.57	-60.62
5	397	vpeak = -397.38$\hat{x}$ + 680.46	0.237434	154.4481	4.59E-25	YES	680.46	-397.38	600.984	283.08	-52.90
6	179	vpeak = -150.99$\hat{x}$ + 409.63	0.09259	103.7243	3.46E-05	YES	409.63	-150.99	379.432	258.64	-31.83
		Lumen diameter table									
dpf	n	Regression model for data from $\hat{x}$ > 0.2	R^2	S.E.R	p value	p<0.05?	val@$\hat{x}$ =0	gradient	val@$\hat{x}$=0.2	val@$\hat{x}$=1	%change btwn $\hat{x}$=0.2 and $\hat{x}$=1
2	208	D = 2.8039$\hat{x}$ + 8.0881	0.15773	1.318596	2.86E-09	YES	8.0881	2.8039	8.64888	10.892	25.94
3	366	D = 2.2416$\hat{x}$ + 9.7643	0.112188	1.322141	4.80E-11	YES	9.7643	2.2416	10.21262	12.0059	17.56
4	404	D = 3.2048$\hat{x}$ + 8.7946	0.278944	1.091908	2.12E-30	YES	8.7946	3.2048	9.43556	11.9994	27.17
5	397	D = 2.3458$\hat{x}$ + 9.175	0.114724	1.41325	4.11E-12	YES	9.175	2.3458	9.64416	11.5208	19.46
6	179	D = 1.4961$\hat{x}$ + 10.003	0.058423	1.317968	1.12E-03	YES	10.003	1.4961	10.30222	11.4991	11.62
		Discharge hematocrit table									
dpf	n	Regression model for data from $\hat{x}$ > 0.2	R^2	S.E.R	p value	p<0.05?	val@$\hat{x}$ =0	gradient	val@$\hat{x}$=0.2	val@$\hat{x}$=1	%change btwn $\hat{x}$=0.2 and $\hat{x}$=1
2	208	Hd = 0.0394$\hat{x}$ + 0.0217	0.056761	0.03265	5.30E-04	YES	0.0217	0.0394	0.02958	0.0611	106.56
3	366	Hd = 0.0274$\hat{x}$ + 0.0234	0.048239	0.025487	2.24E-05	YES	0.0234	0.0274	0.02888	0.0508	75.90
4	404	Hd = 0.0388$\hat{x}$ + 0.0321	0.034318	0.043607	1.81E-04	YES	0.0321	0.0388	0.03986	0.0709	77.87
5	397	Hd = 0.0868$\hat{x}$ + 0.0173	0.121625	0.050593	8.51E-13	YES	0.0173	0.0868	0.03466	0.1041	200.35
6	179	Hd = 0.0967$\hat{x}$ + 0.0239	0.12507	0.0561	1.20E-06	YES	0.0239	0.0967	0.04324	0.1206	178.91
		Viscosity table									
dpf	n	Regression model for data from $\hat{x}$ > 0.2	R^2	S.E.R	p value	p<0.05?	val@$\hat{x}$ =0	gradient	val@$\hat{x}$=0.2	val@$\hat{x}$=1	%change btwn $\hat{x}$=0.2 and $\hat{x}$=1
2	208	η = 2.36E-05$\hat{x}$ + 1.21E-03	0.052622	2.04E-05	8.59E-04	YES	1.21E-03	2.36E-05	0.00121472	0.0012336	1.55
3	366	η = 1.88E-05$\hat{x}$ + 1.21E-03	0.053177	1.66E-05	8.32E-06	YES	1.21E-03	1.88E-05	0.00121376	0.0012288	1.24
4	404	η = 2.90E-05$\hat{x}$ + 1.21E-03	0.045034	2.83E-05	1.70E-05	YES	1.21E-03	2.90E-05	0.0012158	0.001239	1.91
5	397	η = 6.11E-05$\hat{x}$ + 1.20E-03	0.113995	3.69E-05	4.85E-12	YES	1.20E-03	6.11E-05	0.00121222	0.0012611	4.03
6	179	η = 7.08E-05$\hat{x}$ + 1.21E-03	0.115975	4.29E-05	3.09E-06	YES	1.21E-03	7.08E-05	0.00122416	0.0012808	4.63
		Peak WSS table									
dpf	n	Regression model for data from $\hat{x}$ > 0.2	R^2	S.E.R	p value	p<0.05?	val@$\hat{x}$ =0	gradient	val@$\hat{x}$=0.2	val@$\hat{x}$=1	%change btwn $\hat{x}$=0.2 and $\hat{x}$=1
2	208	WSSpeak = -0.2953$\hat{x}$ + 0.6663	0.108803	0.172007	1.14E-06	YES	0.6663	-0.2953	0.60724	0.371	-38.90
3	366	WSSpeak = -0.4055$\hat{x}$ + 0.7164	0.23457	0.153592	6.35E-23	YES	0.7164	-0.4055	0.6353	0.3109	-51.06
4	404	WSSpeak = -0.3411$\hat{x}$ + 0.4565	0.383097	0.091716	4.36E-44	YES	0.4565	-0.3411	0.38828	0.1154	-70.28
5	397	WSSpeak = -0.2296$\hat{x}$ + 0.3505	0.269992	0.081886	7.80E-29	YES	0.3505	-0.2296	0.30458	0.1209	-60.31
6	179	WSSpeak = -0.0813$\hat{x}$ + 0.1958	0.134707	0.045204	4.34E-07	YES	0.1958	-0.0813	0.17954	0.1145	-36.23
		Peak PSR table									
dpf	n	Regression model for data from $\hat{x}$ > 0.2	R^2	S.E.R	p value	p<0.05?	val@$\hat{x}$ =0	gradient	val@$\hat{x}$=0.2	val@$\hat{x}$=1	%change btwn $\hat{x}$=0.2 and $\hat{x}$=1
2	208	PSR = -31.099$\hat{x}$ + 68.598	0.114675	17.58456	5.64E-07	YES	68.598	-31.099	62.3782	37.499	-39.88
3	366	PSR = -81.317$\hat{x}$ + 142.94	0.239724	15.73153	1.84E-23	YES	142.94	-81.317	126.6766	61.623	-51.35
4	404	PSR = -130.88$\hat{x}$ + 173.15	0.381005	9.516098	8.64E-44	YES	173.15	-130.88	146.974	42.27	-71.24
5	397	PSR = -96.568$\hat{x}$ + 151.34	0.278329	8.441778	7.95E-30	YES	151.34	-96.568	132.0264	54.772	-58.51
6	179	PSR = -8.9239$\hat{x}$ + 20.089	0.153369	4.601028	5.93E-08	YES	20.089	-8.9239	18.30422	11.1651	-39.00

Supplementary table 4. Regression statistics for pooled vISV data

vISVs		Peak velocity table									
dpf	n	Regression model for data from $\hat{x} > 0.2$	R^2	S.E.R	p value	p<0.05?	val@$\hat{x}$ =0	gradient	val@$\hat{x}$=0.2	val@$\hat{x}$=1	%change btwn $\hat{x}$=0.2 and $\hat{x}$=1
2	192	vpeak = 167.99$\hat{x}$ + 760.46	0.013583	278.6944	1.07E-01	NO	760.46	167.99	794.058	928.45	16.92
3	393	vpeak = -43.391$\hat{x}$ + 914.56	0.001029	267.6843	5.26E-01	NO	914.56	-43.391	905.8818	871.169	-3.83
4	424	vpeak = -254.72$\hat{x}$ + 574.44	0.124801	137.9997	6.58E-14	YES	574.44	-254.72	523.496	319.72	-38.93
5	384	vpeak = -171.16$\hat{x}$ + 473.51	0.072635	124.4515	8.16E-08	YES	473.51	-171.16	439.278	302.35	-31.17
6	195	vpeak = -51.783$\hat{x}$ + 297.29	0.015081	87.49231	8.72E-02	NO	297.29	-51.783	286.9334	245.507	-14.44
		Lumen diameter table									
dpf	n	Regression model for data from $\hat{x} > 0.2$	R^2	S.E.R	p value	p<0.05?	val@$\hat{x}$ =0	gradient	val@$\hat{x}$=0.2	val@$\hat{x}$=1	%change btwn $\hat{x}$=0.2 and $\hat{x}$=1
2	192	D = 3.5558$\hat{x}$ + 7.8884	0.153962	1.622663	1.81E-08	YES	7.8884	3.5558	8.59956	11.4442	33.08
3	393	D = 1.121$\hat{x}$ + 10.427	0.014178	1.850723	1.82E-02	YES	10.427	1.121	10.6512	11.548	8.42
4	424	D = 2.252$\hat{x}$ + 9.7711	0.091056	1.455606	2.24E-10	YES	9.7711	2.252	10.2215	12.0231	17.63
5	384	D = 1.6307$\hat{x}$ + 9.7844	0.0362	1.712183	1.76E-04	YES	9.7844	1.6307	10.11054	11.4151	12.90
6	195	D = 0.8474$\hat{x}$ + 10.376	0.010286	1.737965	1.58E-01	NO	10.376	0.8474	10.54548	11.2234	6.43
		Discharge hematocrit table									
dpf	n	Regression model for data from $\hat{x} > 0.2$	R^2	S.E.R	p value	p<0.05?	val@$\hat{x}$ =0	gradient	val@$\hat{x}$=0.2	val@$\hat{x}$=1	%change btwn $\hat{x}$=0.2 and $\hat{x}$=1
2	192	Hd = 0.0518$\hat{x}$ + 0.0196	0.082761	0.033597	5.21E-05	YES	0.0196	0.0518	0.02996	0.0714	138.32
3	393	Hd = 0.0412$\hat{x}$ + 0.026	0.054629	0.033964	2.82E-06	YES	0.026	0.0412	0.03424	0.0672	96.26
4	424	Hd = 0.0743$\hat{x}$ + 0.0352	0.068941	0.05583	4.08E-08	YES	0.0352	0.0743	0.05006	0.1095	118.74
5	384	Hd = 0.0954$\hat{x}$ + 0.0286	0.109964	0.055248	2.62E-11	YES	0.0286	0.0954	0.04768	0.124	160.07
6	195	Hd = 0.0865$\hat{x}$ + 0.0617	0.057335	0.073309	7.48E-04	YES	0.0617	0.0865	0.079	0.1482	87.59
		Viscosity table									
dpf	n	Regression model for data from $\hat{x} > 0.2$	R^2	S.E.R	p value	p<0.05?	val@$\hat{x}$ =0	gradient	val@$\hat{x}$=0.2	val@$\hat{x}$=1	%change btwn $\hat{x}$=0.2 and $\hat{x}$=1
2	192	η = 3.44E-05$\hat{x}$ + 1.21E-03	0.091187	2.11E-05	2.07E-05	YES	1.21E-03	3.44E-05	0.00121688	0.0012444	2.26
3	393	η = 2.85E-05$\hat{x}$ + 1.21E-03	0.057265	2.29E-05	1.60E-06	YES	1.21E-03	2.85E-05	0.0012157	0.0012385	1.88
4	424	η = 5.51E-05$\hat{x}$ + 1.22E-03	0.066192	4.23E-05	7.76E-08	YES	1.22E-03	5.51E-05	0.00123102	0.0012751	3.58
5	384	η = 7.09E-05$\hat{x}$ + 1.21E-03	0.115592	3.99E-05	7.61E-12	YES	1.21E-03	7.09E-05	0.00122418	0.0012809	4.63
6	195	η = 7.49E-05$\hat{x}$ + 1.23E-03	0.066841	5.85E-05	2.63E-04	YES	1.23E-03	7.49E-05	0.00124498	0.0013049	4.81
		Peak WSS table									
dpf	n	Regression model for data from $\hat{x} > 0.2$	R^2	S.E.R	p value	p<0.05?	val@$\hat{x}$ =0	gradient	val@$\hat{x}$=0.2	val@$\hat{x}$=1	%change btwn $\hat{x}$=0.2 and $\hat{x}$=1
2	192	WSSpeak = -0.0591$\hat{x}$ + 0.4646	0.006285	0.14458	0.274354	NO	0.4646	-0.0591	0.45278	0.4055	-10.44
3	393	WSSpeak = -0.0675$\hat{x}$ + 0.4466	0.006608	0.163802	0.107617	NO	0.4466	-0.0675	0.4331	0.3791	-12.47
4	424	WSSpeak = -0.1586$\hat{x}$ + 0.2877	0.170444	0.07156	7.00E-19	YES	0.2877	-0.1586	0.25598	0.1291	-49.57
5	384	WSSpeak = -0.1027$\hat{x}$ + 0.2397	0.07519	0.073284	4.74E-08	YES	0.2397	-0.1027	0.21916	0.137	-37.49
6	195	WSSpeak = -0.0275$\hat{x}$ + 0.1434	0.013744	0.048686	0.102641	NO	0.1434	-0.0275	0.1379	0.1159	-15.95
		Peak PSR table									
dpf	n	Regression model for data from $\hat{x} > 0.2$	R^2	S.E.R	p value	p<0.05?	val@$\hat{x}$ =0	gradient	val@$\hat{x}$=0.2	val@$\hat{x}$=1	%change btwn $\hat{x}$=0.2 and $\hat{x}$=1
2	192	PSR = -7.17$\hat{x}$ + 47.963	0.008862	14.76175	0.194028	NO	47.963	-7.17	46.529	40.793	-12.33
3	393	PSR = -7.6955$\hat{x}$ + 45.939	0.008109	16.85233	0.074576	NO	45.939	-7.6955	44.3999	38.2435	-13.87
4	424	PSR = -16.879$\hat{x}$ + 29.467	0.179376	7.385866	6.95E-20	YES	29.467	-16.879	26.0912	12.588	-51.75
5	384	PSR = -11.151$\hat{x}$ + 24.548	0.08265	7.559699	9.65E-09	YES	24.548	-11.151	22.3178	13.397	-39.97
6	195	PSR = -3.4564$\hat{x}$ + 14.567	0.020686	4.972176	0.044862	YES	14.567	-3.4564	13.87572	11.1106	-19.93

Supplementary table 5. Regression statistics for pooled DLAV data

DLAV		Peak velocity table									
dpf	n	Regression model for data from $\hat{x}$ > 0.2	R^2	S.E.R	p value	p<0.05?	val@$\hat{x}$ =0	gradient	val@$\hat{x}$=0.2	val@$\hat{x}$=1	%change btwn $\hat{x}$=0.2 and $\hat{x}$=1
2	197	vpeak = 197.32$\hat{x}$ + 365.9	0.018872	253.7169	0.054227	NO	365.9	197.32	405.364	563.22	38.94
3	408	vpeak = -189.75$\hat{x}$ + 649.64	0.021001	243.6935	0.003349	YES	649.64	-189.75	611.69	459.89	-24.82
4	409	vpeak = -82.75$\hat{x}$ + 361.89	0.014032	137.1874	0.016543	YES	361.89	-82.75	345.34	279.14	-19.17
5	366	vpeak = -37.239$\hat{x}$ + 293.28	0.003421	123.8012	0.264377	NO	293.28	-37.239	285.8322	256.041	-10.42
6	128	vpeak = -2.756$\hat{x}$ + 260.39	2.13E-05	113.1047	0.958759	NO	260.39	-2.756	259.8388	257.634	-0.85
		Lumen diameter table									
dpf	n	Regression model for data from $\hat{x}$ > 0.2	R^2	S.E.R	p value	p<0.05?	val@$\hat{x}$ =0	gradient	val@$\hat{x}$=0.2	val@$\hat{x}$=1	%change btwn $\hat{x}$=0.2 and $\hat{x}$=1
2	197	D = 0.7463$\hat{x}$ + 11.22	0.004031	2.091918	0.375454	NO	11.22	0.7463	11.36926	11.9663	5.25
3	408	D = -1.0082$\hat{x}$ + 13.244	0.008374	2.063734	0.064801	NO	13.244	-1.0082	13.04236	12.2358	-6.18
4	409	D = -1.1859$\hat{x}$ + 12.05	0.018707	1.698762	0.005595	YES	12.05	-1.1859	11.81282	10.8641	-8.03
5	366	D = 0.1374$\hat{x}$ + 10.867	0.000279	1.602076	0.750203	NO	10.867	0.1374	10.89448	11.0044	1.01
6	128	D = 0.671$\hat{x}$ + 11.153	0.005319	1.738183	0.413304	NO	11.153	0.671	11.2872	11.824	4.76
		Discharge hematocrit table									
dpf	n	Regression model for data from $\hat{x}$ > 0.2	R^2	S.E.R	p value	p<0.05?	val@$\hat{x}$ =0	gradient	val@$\hat{x}$=0.2	val@$\hat{x}$=1	%change btwn $\hat{x}$=0.2 and $\hat{x}$=1
2	197	Hd = 0.0164$\hat{x}$ + 0.054	0.004384	0.044144	0.355268	NO	0.054	0.0164	0.05728	0.0704	22.91
3	408	Hd = 0.0314$\hat{x}$ + 0.0452	0.020382	0.040955	0.003856	YES	0.0452	0.0314	0.05148	0.0766	48.80
4	409	Hd = 0.0413$\hat{x}$ + 0.0509	0.022111	0.05429	0.002572	YES	0.0509	0.0413	0.05916	0.0922	55.85
5	366	Hd = 0.0334$\hat{x}$ + 0.0474	0.01772	0.048413	0.010793	YES	0.0474	0.0334	0.05408	0.0808	49.41
6	128	Hd = 0.0566$\hat{x}$ + 0.054	0.024292	0.067923	0.07895	NO	0.054	0.0566	0.06532	0.1106	69.32
		Viscosity table									
dpf	n	Regression model for data from $\hat{x}$ > 0.2	R^2	S.E.R	p value	p<0.05?	val@$\hat{x}$ =0	gradient	val@$\hat{x}$=0.2	val@$\hat{x}$=1	%change btwn $\hat{x}$=0.2 and $\hat{x}$=1
2	197	η = 1.29E-05$\hat{x}$ + 1.23E-03	0.004921	3.27E-05	0.327308	NO	1.23E-03	1.29E-05	0.00123258	0.0012429	0.84
3	408	η = 2.00E-05$\hat{x}$ + 1.23E-03	0.013355	3.23E-05	0.019547	YES	1.23E-03	2.00E-05	0.001234	0.00125	1.30
4	409	η = 2.28E-05$\hat{x}$ + 1.23E-03	0.012786	3.96E-05	0.022188	YES	1.23E-03	2.28E-05	0.00123456	0.0012528	1.48
5	366	η = 2.44E-05$\hat{x}$ + 1.23E-03	0.019453	3.37E-05	0.007534	YES	1.23E-03	2.44E-05	0.00123488	0.0012544	1.58
6	128	η = 4.37E-05$\hat{x}$ + 1.23E-03	0.025233	5.14E-05	0.073303	NO	1.23E-03	4.37E-05	0.00123874	0.0012737	2.82
		Peak WSS table									
dpf	n	Regression model for data from $\hat{x}$ > 0.2	R^2	S.E.R	p value	p<0.05?	val@$\hat{x}$ =0	gradient	val@$\hat{x}$=0.2	val@$\hat{x}$=1	%change btwn $\hat{x}$=0.2 and $\hat{x}$=1
2	197	WSSpeak = 0.0803$\hat{x}$ + 0.1633	0.014253	0.119138	0.094724	NO	0.1633	0.0803	0.17936	0.2436	35.82
3	408	WSSpeak = -0.0565$\hat{x}$ + 0.2479	0.011151	0.10009	0.03297	YES	0.2479	-0.0565	0.2366	0.1914	-19.10
4	409	WSSpeak = -0.0197$\hat{x}$ + 0.1496	0.004391	0.058587	0.181039	NO	0.1496	-0.0197	0.14566	0.1299	-10.82
5	366	WSSpeak = -0.0161$\hat{x}$ + 0.1341	0.002843	0.058864	0.309008	NO	0.1341	-0.0161	0.13088	0.118	-9.84
6	128	WSSpeak = 0.000484$\hat{x}$ + 0.1134	3.51E-06	0.048923	0.983256	NO	0.1134	0.000484	0.1134968	0.113884	0.34
		Peak PSR table									
dpf	n	Regression model for data from $\hat{x}$ > 0.2	R^2	S.E.R	p value	p<0.05?	val@$\hat{x}$ =0	gradient	val@$\hat{x}$=0.2	val@$\hat{x}$=1	%change btwn $\hat{x}$=0.2 and $\hat{x}$=1
2	197	PSR = 8.0332$\hat{x}$ + 16.525	0.013947	12.04512	0.098365	NO	16.525	8.0332	18.13164	24.5582	35.44
3	408	PSR = -5.984$\hat{x}$ + 25.14	0.012198	10.12948	0.025691	YES	25.14	-5.984	23.9432	19.156	-19.99
4	409	PSR = -2.3254$\hat{x}$ + 15.248	0.005857	5.991669	0.122268	NO	15.248	-2.3254	14.78292	12.9226	-12.58
5	366	PSR = -1.8249$\hat{x}$ + 13.666	0.003464	6.029095	0.261404	NO	13.666	-1.8249	13.30102	11.8411	-10.98
6	128	PSR = -0.3819$\hat{x}$ + 11.545	0.000217	4.912894	0.868981	NO	11.545	-0.3819	11.46862	11.1631	-2.66

Legend for supplemental videos

Video 1: Representative RBC flow in zebrafish from the high throughput experiment at 2 dpf. Shown in the video is zebrafish #28 from the 2 dpf population group with flow recorded at 180 fps for 1000 frames. The video is played back at 180 fps.

Video 2: Representative RBC flow in zebrafish from the high throughput experiment at 3 dpf. Shown in the video is zebrafish #30 from the 3 dpf population group with flow recorded at 180 fps for 1000 frames. The video is played back at 180 fps.

Video 3: Representative RBC flow in zebrafish from the high throughput experiment at 4 dpf. Shown in the video is zebrafish #37 from the 4 dpf population group with flow recorded at 120 fps for 1000 frames. The video is played back at 120 fps.

Video 4: Representative RBC flow in zebrafish from the high throughput experiment at 5 dpf. Shown in the video is zebrafish #33 from the 5 dpf population group with flow recorded at 100 fps for 1000 frames. The video is played back at 100 fps.

Video 5: Representative RBC flow in zebrafish from the high throughput experiment at 6 dpf. Shown in the video is zebrafish #29 from the 6 dpf population group with flow recorded at 100 fps for 1000 frames. The video is played back at 100 fps.

Video 6: RBC flow in zebrafish at 2 dpf imaged at 80x magnification for the validation experiments. Shown in the video is zebrafish C1 that received control morpholino treatment resulting in normal hematocrit levels. The blood flow was recorded at 100 fps for 2000 frames. The video is played back at 100 fps.

Video 7: RBC flow in zebrafish at 2 dpf imaged at 80x magnification for the validation experiments. Shown in the video is zebrafish C2 that received control morpholino treatment resulting in normal hematocrit levels. The blood flow was recorded at 100 fps for 2000 frames. The video is played back at 100 fps.

Video 8: RBC flow in zebrafish at 2 dpf imaged at 80x magnification for the validation experiments. Shown in the video is zebrafish M1 that received gata1 morpholino treatment resulting in moderately reduced hematocrit levels. The blood flow was recorded at 100 fps for 2000 frames. The video is played back at 100 fps.

Video 9: RBC flow in zebrafish at 2 dpf imaged at 80x magnification for the validation experiments. Shown in the video is zebrafish M2 that received gata1 morpholino treatment resulting in moderately reduced hematocrit levels. The blood flow was recorded at 100 fps for 2000 frames. The video is played back at 100 fps.

Video 10: RBC flow in zebrafish at 2 dpf imaged at 80x magnification for the validation experiments. Shown in the video is zebrafish M3 that received gata1 morpholino treatment resulting in moderately reduced hematocrit levels. The blood flow was recorded at 100 fps for 2000 frames. The video is played back at 100 fps.

Video 11: RBC flow in zebrafish at 2 dpf imaged at 80x magnification for the validation experiments. Shown in the video is zebrafish M4 that received gata1 morpholino treatment resulting in greatly reduced hematocrit levels. The blood flow was recorded at 100 fps for 2000 frames. The video is played back at 100 fps.

Video12: RBC flow in zebrafish at 2 dpf imaged at 80x magnification for the validation experiments. Shown in the video is zebrafish M5 that received gata1 morpholino treatment resulting in greatly reduced hematocrit levels. The blood flow was recorded at 100 fps for 2000 frames. The video is played back at 100 fps.

SV13: Video montage showing the spatial distribution map of hemodynamic and morphological data in all zebrafish obtained from the high throughput experiment after automated spatial bin averaging
